# A Cas12a ortholog with stringent PAM recognition followed by low off-target editing rates for genome editing

**DOI:** 10.1186/s13059-020-01989-2

**Published:** 2020-03-25

**Authors:** Peng Chen, Jin Zhou, Yibin Wan, Huan Liu, Yongzheng Li, Zhaoxin Liu, Hongjian Wang, Jun Lei, Kai Zhao, Yiliang Zhang, Yan Wang, Xinghua Zhang, Lei Yin

**Affiliations:** 1 State Key Laboratory of Virology, Hubei Key Laboratory of Cell Homeostasis, Department of Biochemistry and Molecular Biology, College of Life Sciences, Wuhan University, Wuhan, China; 2 Department of Clinical Oncology, Renmin Hospital of Wuhan University, Wuhan, 430060 China; 3 Hubei Key Laboratory of Cell Homeostasis, College of Life Sciences, Wuhan University, Wuhan, 430072 Hubei People’s Republic of China; 4 College of Life Sciences, the Institute for Advanced Studies, State Key Laboratory of Virology, Hubei Key Laboratory of Cell Homeostasis, Wuhan University, Wuhan, 430072 China

**Keywords:** CRISPR, Cas12a, Gene editing, PAM stringency, Off-targeting

## Abstract

**Background:**

AsCas12a and LbCas12a nucleases are reported to be promising tools for genome engineering with protospacer adjacent motif (PAM) TTTV as the optimal. However, the C-containing PAM (CTTV, TCTV, TTCV, etc.) recognition by Cas12a might induce extra off-target edits at these non-canonical PAM sites.

**Results:**

Here, we identify a novel Cas12a nuclease CeCas12a from *Coprococcus eutactus*, which is a programmable nuclease with genome-editing efficiencies comparable to AsCas12a and LbCas12a in human cells. Moreover, CeCas12a is revealed to be more stringent for PAM recognition in vitro and in vivo followed by very low off-target editing rates in cells. Notably, CeCas12a renders less off-target edits located at C-containing PAM at multiple sites compared to LbCas12a and AsCas12a, as assessed by targeted sequencing methods.

**Conclusions:**

Our study shows that CeCas12a nuclease is active in human cells and the stringency of PAM recognition could be an important factor shaping off-target editing in gene editing. Thus, CeCas12a provides a promising candidate with distinctive characteristics for research and therapeutic applications.

## Background

Clustered regularly interspaced short palindromic repeats (CRISPR)/CRISPR-associated nucleases (Cas), which are essential for bacterial adaptive immunity, have been exploited to develop potent tools for genome manipulation in cells and organisms and have widespread applications in research, medicine, and biotechnology [1–10]. Besides the most commonly used *Streptococcus pyogenes* Cas9 (SpCas9), a series of Cas9 orthologs from different organisms, such as *Staphylococcus aureus* (Sa), *Streptococcus thermophilus* (St), and *Neisseria meningitidis* (Nm), have been exploited [11–13]. However, SpCas9 presents unsurpassed activity in gene editing and genome manipulation.

Recently, CRISPR-Cas12a/Cpf1 was reported to be a highly specific programmable nuclease with high efficiency comparable to Cas9 [14–16]. Several different features make Cas12a an important expansion of CRISPR-based genome-editing tools [14]. First, Cas12a needs only a single crRNA processed to be mature by itself without the requirement of a trans-activating RNA (tracrRNA) and double-stranded (ds) RNA-specific ribonuclease RNase III, which are indispensable for maturation of Cas9 crRNA. Second, Cas12a requires thymine-rich protospacer adjacent motif (PAM) sequence at the 5′ end of the protospacer, different from the guanine-rich PAM sequences at the 3′ end of the target DNA for Cas9 systems. Third, after cleavage of double-stranded DNAs, Cas12a generates a staggered double-strand break resulting in 4 or 5 nt 5′-overhangs distal to the PAM site; however, Cas9 creates blunt ends within the PAM-proximal target site. Due to the features discussed above, Cas12a can be an alternative even better tool for genome editing in some situations. Moreover, it was suggested that Cas12a is highly specific in human cells with low off-target rates from the study of two types of Cas12a (*Acidaminococcus* sp. *BV3L6*, AsCas12a, and *Lachnospiraceae bacterium ND 2006*, LbCas12a) [17, 18].

These distinct features of Cas12a provide good potential for the development of genomic editing tools. However, some challenges may still hinder the application of CRISPR-Cas systems, such as how some certain factors shape off-targeting and how to reduce that. We noticed off-target effects at non-canonical PAM region from the previous study of Feng Zhang and his colleagues which adopted engineered Cas12a variants to alter PAM specificities for increasing genome targeting range [19]. From this clue, a novel Cas12a nuclease from *Coprococcus eutactus* (CeCas12a) was identified to be more restrictive in the selection of PAM sequences in vitro and in vivo than AsCas12a and LbCas12a, followed by the lower off-targeting in cells at the PAM region, and the off-targeting of CeCas12a at other regions was also lower in general. Besides, the efficiency of CeCas12a in editing human cells was comparable to well-accepted AsCas12a and LbCas12a. All these suggested CeCas12a could be a good expansion of accurate CRISPR-based genome-editing tools and stringency for PAM recognition might be an important factor shaping off-targeting which could lead us to find more useful accurate genome-editing tools.

## Results

### Cas12a orthologs with different stringencies for recognizing canonical TTTV and non-canonical C-containing PAMs in vitro

**Fig. 1:**
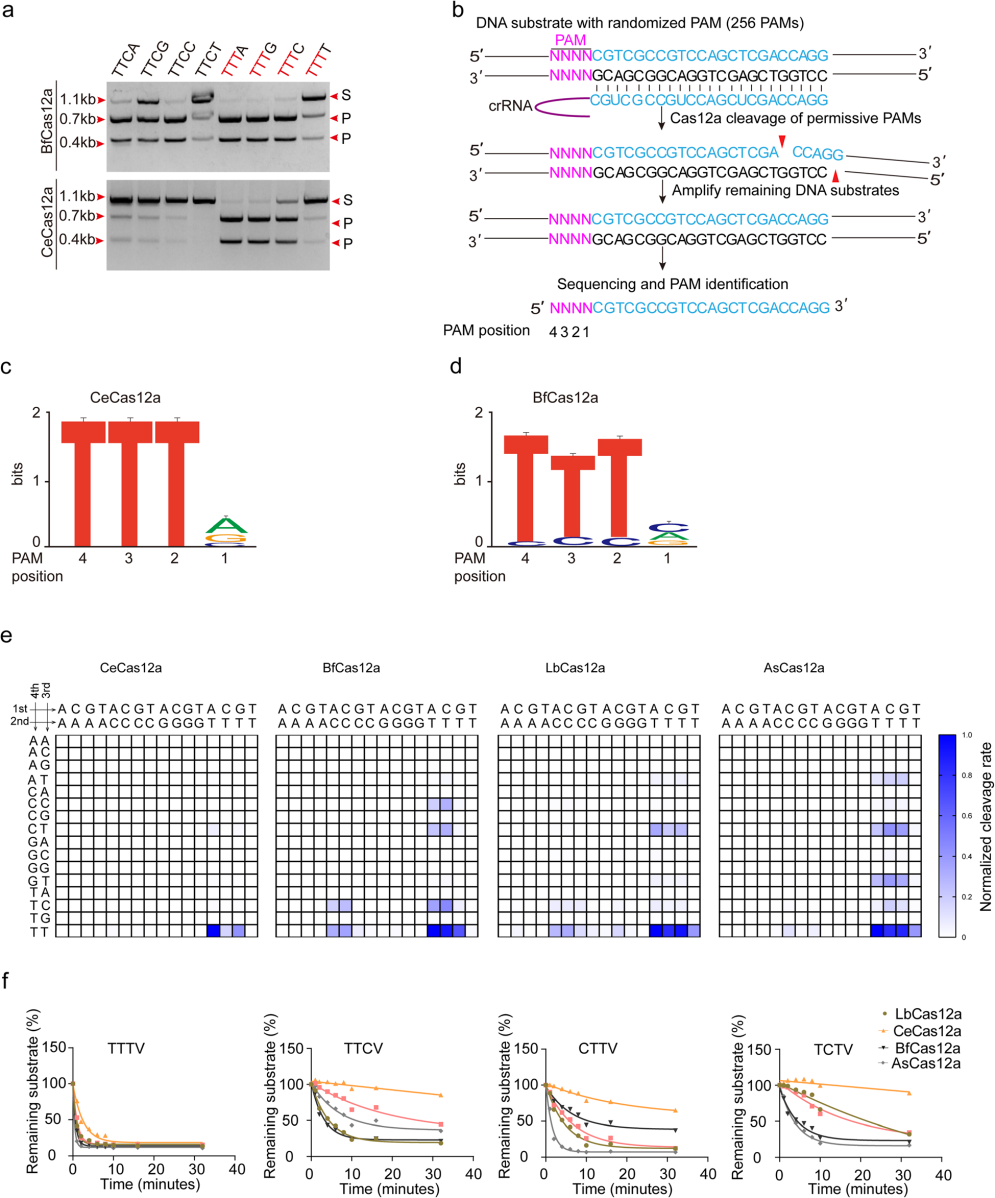
DNA cleavage of Cas12a orthologs in vitro. **a** DNA cleavage activities of BfCas12a and Cas12a in vitro. The Cas12a-crRNAcomplex (100 nM) was incubated at 37 °C for 10 min with DNA substrates (see also Additional file 1: Figure S4). **b** Schematic of in vitro cleavage assay used to identify PAM sequence and determine global PAM specificity. **c** Web logo for the CeCas12a PAM. **d** Web logo for the BfCas12a PAM. **e** Normalized cleavage rates for all 4-base PAMs for CeCas12a, BfCas12a, LbCas12a, and AsCas12a. The intensity of color represented the activity of Cas12a nuclease. **f** Quantification of time-course in vitro cleavage reactions of Cas12a orthologs on linearized dsDNA substrates. Those were conducted at nine time points, respectively. Curves were fit using the one phase exponential decay equation

Cas12a (Cpf1) nucleases such as AsCas12a, LbCas12a, and FnCas12a have been used as gene-editing tools in biological researches with the requirement of recognition of specific PAM sequences, and non-canonical PAM regions were also recognized in some extent [14, 15, 20]. From previous others’ data, we noticed off-target effects could happen at non-canonical PAM regions [19, 21]. To further investigate the stringency for recognizing non-canonical PAMs and the related off-target effects at non-canonical PAMs region by different Cas12a orthologs, 12 Cas12a nucleases were selected including four Cas12a nucleases (CeCas12a, PrCas12a from *Prevotella ruminicola strain BPI-34*, CsbCas12a from *Candidatus Saccharibacteria bacterium*, BhCas12 from *Butyrivibrio hungatei strain MB2003*) which have not previously been reported, 3 Cas12a nucleases (AsCas12a, LbCas12a, FnCas12a) with activity for genome manipulation, and another 5 reported Cas12a nucleases (SsCas12a from *Smithella* sp. *SC_K08D17*, Lb3Cas12a from *Lachnospiraceae bacterium MC2017*, BpCas12a from *Bytyrivibrio*, PdCas12a from *Prevotella disens*, BfCas12a from *Butyrivibrio fibrisolvens MD2001*) which have not been employed for genome editing on endogenous sites in human cells (Additional file 1: Figure S1A) [14, 22]. Their crRNA arrays were identified from their genomic loci (Additional file 2: Table S1). Two hundred fifty-six kinds of linear dsDNA substrates were synthesized consisting of 4 randomized nucleotides upstream of the protospacer with overlap PCR (Additional file 1: Figure S3A). Then, the Cas12a nucleases expressed in *Escherichia coli* cells were purified (Additional file 1: Figure S2A, B) and incubated with in vitro-transcribed crRNAs and dsDNA substrates for in vitro DNA cleavage assays (Additional file 1: Figure S3B). Except for AsCas12a, LbCas12a, and FnCas12a, which had been reported to be with DNA cleavage activity in vitro and in vivo, only CeCas12a and BfCas12a were able to cleave dsDNA in vitro and were chosen for further investigations (Additional file 1: Figure S4, 5, 6, 7). CeCas12a and BfCas12a could recognize 5′-TTTN PAM to cleave the linear dsDNA substrates (Fig. 1a; Additional file 1: Figure S4A, B), which is similar to AsCas12a and LbCas12a [14]. The DNA cleavage activities of LbCas12a and AsCas12a have been investigated both in vitro [14, 23] and in vivo [17, 18, 24]. Similarly, with the purpose to check the characters of these Cas12a orthologs for genome editing, we measured dsDNA cleavage activities of all these Cas12a orthologs in vitro using linear dsDNA substrates with 23-nt target sequences and the TTTA PAM (Additional file 1: Figure S8A). CeCas12a and BfCas12a present robust DNA cleavage activity in vitro (Additional file 1: Figure S8A). To further determine the PAM of Ce and Bf, we adapted an in vitro PAM identification assay. We incubated Cas12a RNP complex and 150-bp dsDNA substrates containing 4 randomized nucleotides upstream of the same target spacer. By amplifying and deep sequencing the remaining DNA substrates and comparing with the negative control, we found that CeCas12a and BfCas12a also recognize rich T PAMs (Fig. 1b, d). We also detected whether the fourth nucleotide of the PAM could influence the cleavage activities of Cas12a orthologs; CeCas12a and BfCas12a nucleases showed similar cleavage activities. Both efficiently cleaved the TTTA, TTTG, and TTTC sites, but slightly cleaved the TTTT site (Fig. 1a). Although Cas12a orthologs recognized the TTTV as the optimal PAM, AsCas12a and Lb Cas12a also recognized C-containing PAMs, such as CTTV, TCTV, and TTCV (V is A, G, or C) [23, 25].

Both CeCas12a and BfCas12a recognized the TTTV PAM and C-containing PAMs (Fig. 1a, c, d; Additional file 1: Figure S4A, B). However, for CeCas12a, the suboptimal C-containing PAM [25] substrates were slightly cleaved (such as CTTV and TTCV PAMs) or even barely cleaved (such as TCTV and CCTV PAMs) while the optimal PAM TTTV substrates were cleaved completely (Additional file 1: Figure S4A). To assess the PAM preference of CeCas12a and BfCas12a and compare them with two widely used Cas12a nucleases (AsCas12a, LbCas12a), we systematically studied reactions in parallel for Cas12a nucleases by incubating for a different amount of time for each candidate to assess cleavage kinetics (Fig. 1e, f and Additional file 3). Normalized cleavage rate reaches maximum at TTTV PAM (Fig. 1e), and it supports TTTV as the optimal PAM for CeCas12a, BfCas12a, AsCas12a, and LbCas12a. Certain PAMs could also be recognized by Cas12a nucleases as suboptimal candidates (Fig. 1e). In detail, AsCas12a, LbCas12a, and BfCas12a cleaved other dsDNA substrates including TCTV, TTCV, and CTTV at detectable rates (Fig. 1e, f). CeCas12a had the comparable cleavage rate to AsCas12a and LbCas12a toward TTTV PAM (Fig. 1e, f). However, CeCas12a had a lower cleavage rate at CTTV and nearly undetectable rates at TCTV and TTCV, compared to AsCas12a and LbCas12a at those PAMs (Fig. 1e, f). Their cleavage activities in vitro were also systematically measured at the same target site with 7 different C-containing PAMs (TCTA, TTCA, TCCA, CTTA, CTCA, CCTA, and CCCA) (Additional file 1: Figure S8B). Five Cas12a nucleases show different cleavage activities at the same PAM site. For the optimal TTTA site, all Cas12a nucleases present robust DNA cleavage activities and nearly all substrates were cleaved (Additional file 1: Figure S8B). Although C-containing PAM substrates could be cleaved by all five Cas12a nucleases, the efficacies were lower than TTTA PAM substrate (Additional file 1: Figure S8B). Especially for CeCas12a, there were few cleavages at C-containing PAM sites except the CTTA PAM site (Additional file 1: Figure S8B). To further confirm their specificities or stringency for different PAMs, we incubated each Cas12a RNP and dsDNA substrates containing TTTV, TCTV, TTCV, and CTTV (V = A, G, and C) in parallel at nine different amount of time (0 min, 1 min, 2 min, 4 min, 6 min, 8 min, 10 min, 16 min, 32 min), by deep sequencing the remaining DNA substrates and comparing them with the negative control, in order to assess cleavage kinetics (Additional file 1: Figure S8C). As expected, all Cas12a nucleases were most active at TTTV (V = A, G, and C) and Ce presented little activities at those C-containing PAMs.

All these results showed that AsCas12a and LbCas12a recognized TTTV and non-canonical C-containing PAMs in vitro, which were consistent with previous studies [23, 25]. Furthermore, another two novel Cas12a nucleases (BfCas12a and CeCas12a) were identified with comparable cleavage efficiencies to AsCas12a, LbCas12a, and FnCas12a in vitro. Among them, CeCas12a was the least active at TCTA, TTCA, and CTTA PAMs, even barely active with some PAMs such as TCCA, CTCA, and CCTA (Fig. 1e, f; Additional file 1: Figure S8B, C). CeCas12a turned out to be more specific on PAM recognition in vitro, which drove us to further investigate whether this could shape off-targeting in cells.

### Gene editing with CeCas12a and BfCas12a in human cells

**Fig. 2:**
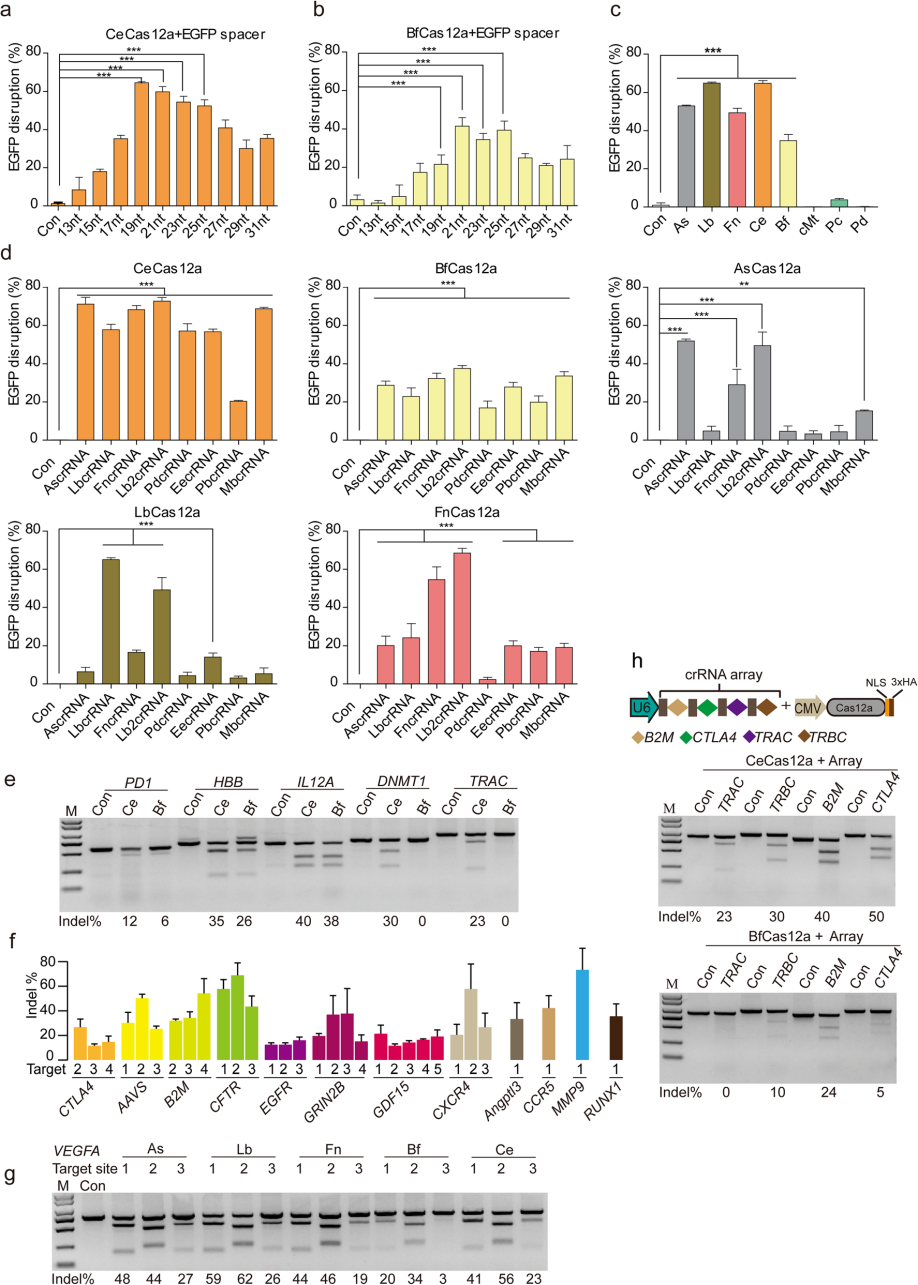
CeCas12a and BfCas12a mediate gene editing in human cells. **a** Efficiencies of EGFP disruption in human cells mediated by CeCas12a and crRNAs bearing variable-length complementarity regions for the target site of EGFP in human cells. Error bars indicate standard errors of means (s.e.m.), *n* = 3. ***P < 0.001 (Mann-Whitney). **b** Efficiencies of EGFP disruption mediated by BfCas12a and crRNAs bearing variable-length complementarity regions for the target site of EGFP in human cells. Error bars represent s.e.m., *n* = 3. ***P < 0.001 (Mann-Whitney). **c** Efficiencies of EGFP disruption in human cells mediated by Cas12a orthologs and crRNA bearing 23-nt length complementarity regions for the target site of EGFP in human cells. Error bars represent s.e.m., *n* = 3. ***P < 0.001 (Mann-Whitney). **d** The suitability of Cas12a with crRNAs containing different loop regions from Cas12a orthologs. Data are shown as mean ± s.e.m. (*n* = 3); **P < 0.01, ***P < 0.001 (Mann-Whitney). **e** Efficiencies of targeted indel mutations introduced at five different human endogenous gene targets by CeCas12a and BfCas12a. Indel frequencies of *PD1*, *HBB*, *IL12A*, *DNMT1*, and *TRAC* were measured by T7E1 assay. **f** Summaries of the activities of CeCas12a at TTTV PAMs at a diverse panel of target sites in HEK293T cells. For indel percentages, each column represents the mean of *n* = 3 transfected cell cultures. Indel frequencies were analyzed by T7E1 assay. **g** Comparison of CeCas12a and Cas12a orthologs’ gene-editing efficiencies. Three target sites are from *VEGFA* loci. Indel frequencies analyzed by T7E1 assay. **h** Cas12a-mediated multiplex gene editing in human cells. Schematic of CRISPR array construct with the U6 direct repeat spacer cassette containing *B2M*, *CTLA4*, *TRAC*, and *TRBC* protospacer sequences (top). Indel frequencies were analyzed by T7E1 assay after transfection with CRISPR array and Cas12a plasmids (bottom)

Cas12a can be programmed to induce DNA double-strand breaks (DSBs) at specific genomic loci in vivo [14, 15, 17, 18, 20, 25, 26]. To test if CeCas12a and BfCas12a could be harnessed for gene editing in mammalian cells, we optimized the codons of two genes and attached a C-terminal and an N-terminal nuclear localization signals (NLS) for optimal expression and nuclear targeting in human cells. They were then cloned into mammalian expression vector pcDNA3.1. In order to monitor the activity of CeCas12a and BfCas12a, a series of crRNAs with different lengths of spacers (13 nt, 15 nt, 17 nt, 19 nt, 21 nt, 23 nt, 25 nt, 27 nt, 29 nt, 31 nt) targeting the same site of EGFP were cloned into pU6-Fn-crRNA vector (Addgene number #78958) which was driven by a U6 promoter. To detect cleavage efficiency of CeCas12a and BfCas12a nucleases, we adopted an EGFP disruption assay [27–29] that was based on generations of frameshift indel mutations, thus leading to the loss of fluorescence. Significant EGFP disruption was observed for crRNAs with 19–25 nt spacer sequences, which are commonly used for Cas12a, and over 60% EGFP disruption efficiency for CeCas12a (Fig. 2a) and 40% for BfCas12a were achieved (Fig. 2b). To provide appropriate controls, we also identified two inactivated RuvC nuclease domain mutants for CeCas12a (D880A, E975A) and BfCas12a (D834A, E925A) nucleases, based on the sequence similarity of these nucleases [14, 30] (Fig. 2a, b; Additional file 1: Figure S9A, B, C, D). To further confirm the in vivo activity, the cleavage assay was refined by using 23 nt spacer crRNA targeting the same site of EGFP with six Cas12a nucleases (AsCas12a, LbCas12a, FnCas12a, PcCas12a, PdCas12a, and cMtCas12a) which had been reported before [14]. The results also showed that CeCas12a and BfCas12a displayed robust EGFP disruption activities when pairing an EGFP-targeted crRNA (Fig. 2c; Additional file 1: Figure S10).

Previous studies suggested that the crRNA carrying nucleotide substitutions in the direct repeats had variable effects on the cleavage activity of Cas12a nucleases [15, 31]. To investigate the effects of mutations in the loop region, we employed 8 crRNA loop regions (AsCas12a, 5-UCUU-3; LbCas12a, 5-UAAGU-3; FnCas12a/BfCas12a/CeCas12a, 5-UGUU-3; PbCas12a/PeCas12a/LiCas12a, 5-UUUU-3; Lb2Cas12a/PmCas12a/PcCas12a, 5-UAUU; MbCas12a, 5-UGUUU-3; PdCas12a, 5-UUCG-3; EeCas12a, 5-UUU-3) from 14 Cas12a orthologs and combined these crRNA members with different Cas12a nucleases (Additional file 1: Figure S1B). The EGFP disruption ratio analysis by FACS revealed that the highest cleavage efficiency was achieved by the Cas12a nuclease with its cognate crRNA loop region in most cases (Fig. 2d). Furthermore, all five Cas12a nucleases can utilize crRNA spacer with Lb2crRNA loop region to cleave target DNA which is consistent with the previous studies of As/Lb/Fn Cas12a nucleases [15, 18, 32] (Fig. 2d). Among them, CeCas12a could nearly accommodate all different crRNA loop regions to achieve impressive cleavage efficiencies (Fig. 2d).

We further explored the capability of CeCas12a and BfCas12a to cleave different genomic loci (*HBB*, *DNMT1*, *IL12A*, *TRAC*, and *PD1*) in human cells and quantified gene-editing efficiencies (Fig. 2e). The results of the T7E1 assay showed that CeCas12a and BfCas12a could generate DNA double-strand breaks in human 293T cells. Deep sequencing results further confirmed the ability of CeCas12a and BfCas12a to introduce insertions or deletions (indels) at target sites in mammalian genomes (Additional file 1: Figure S11). In some cases, BfCas12a almost lost the potential for gene editing. In contrast, CeCas12a could perform pretty well in all 5 genomic targets (Fig. 2e). Furthermore, we detected another 31 endogenous target sites located on *CTLA4*, *B2M*, *AAVS*, *CFTR*, *EGFR*, *GRIN2B*, *GDF15*, *CXCR4*, *Angptl3*, *CCR5*, *MMP9*, and *RUNX1*, and the mean frequencies of indels range from 8 to 65% (Fig. 2f; Additional file 1: Figure S12). For evaluating the gene-editing potential of CeCas12a and BfCas12a along with well-accepted As/Lb/Fn Cas12a nucleases, three target sites of *VEGFA* were checked. All three sites could be successfully cleaved in human cells with these five Cas12a nucleases (Fig. 2g). The maximum cleavage efficiency of CeCas12a was about 56% at site 2, while BfCas12a presented the modest cleavage activity at *VEGFA* site 1 and site 2 (Fig. 2g).

Cas12a alone was able to process pre-crRNA and generate mature crRNA [14, 33]. So it required only a Pol III promoter to drive transcription and maturation of multiple crRNAs. Thus, we detected whether CeCas12a and BfCas12a retained the activities of multiplex gene editing by using a single crRNA array that contains 4 crRNAs targeting 4 human genes (*TRAC*, *TRBC*, *B2M*, *CTLA4*). The T7E1 assay showed that CeCas12a achieved impressive cleavage efficiency with about 50% at *CTLA4*, 40% at *B2M*, 30% at *TRBC*, and 23% at *TRAC*. In contrast, BfCas12a also achieved about 24% efficiency at B2M, 10% at *TRBC*, and 5% at *CTLA4*, but no detectable efficiency at *TRAC* (Fig. 2h).

Taken together, these results further showed that CeCas12a and BfCas12a could be used to introduce insertions or deletions (indels) at target sites in human cells and could also be used for multiplex gene editing.

### Cas12a orthologs with different stringencies for recognizing canonical TTTV and non-canonical C-containing PAMs in human cells

**Fig. 3:**
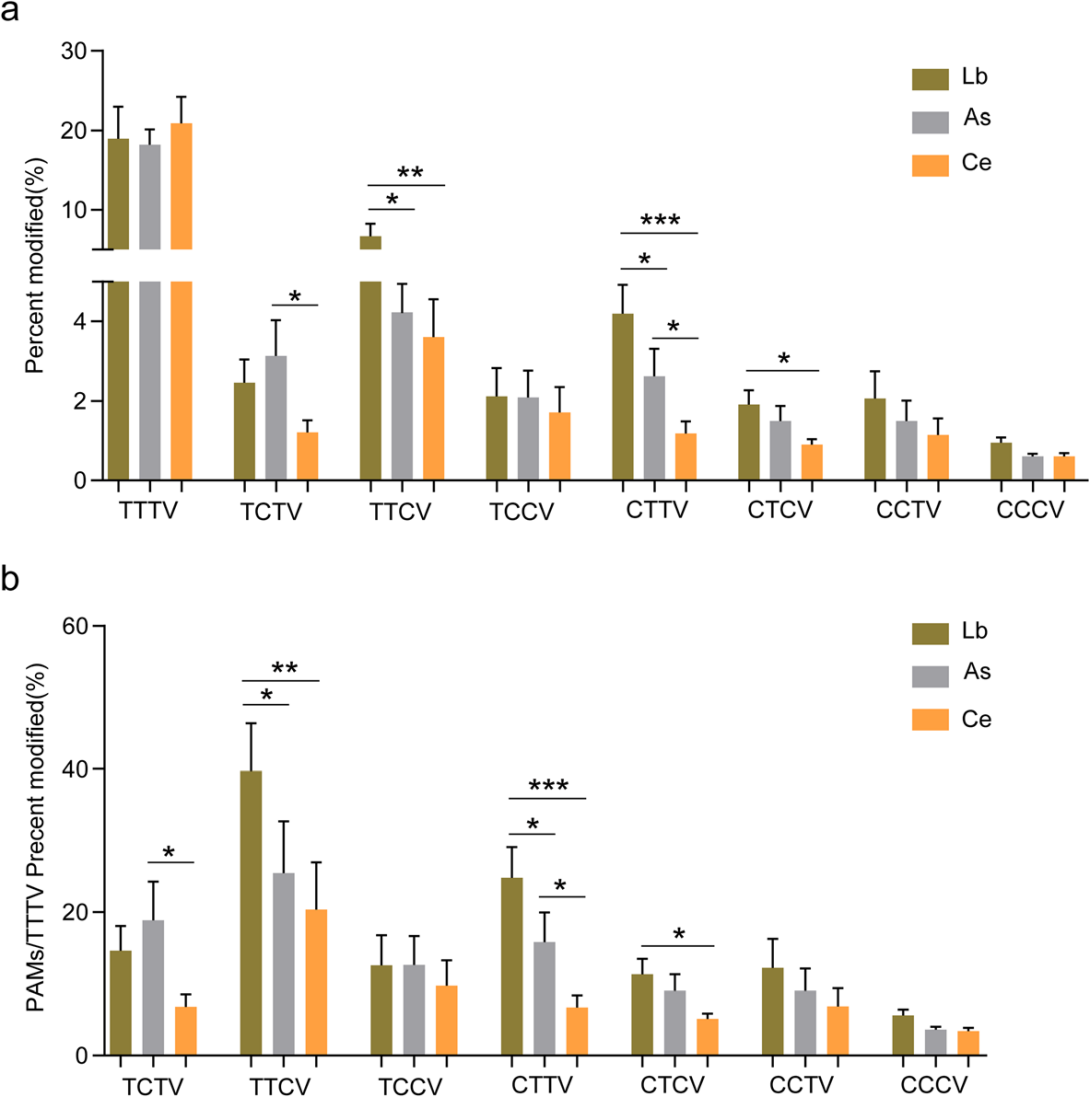
The stringency of CeCas12a, LbCas12a, and AsCas12a for recognizing canonical TTTV and non-canonical C-containing PAMs in human cells. **a** In vivo cleavage activities of Lb, As, and Ce toward different PAMs. Indel frequencies for 82 endogenous target sites with the different PAMs were measured in mammalian cells by deep sequencing. Percent modified indicates the percentage of reads containing indels compared with the wild-type sequence. **b** Stringency for recognizing canonical TTTV and non-canonical C-containing PAMs. The relative percent modified frequency was determined by the ratio of C-containing PAM to TTTV PAM. Data are shown as mean ± s.e.m. (*n* = 3); *P < 0.05, **P < 0.01, ***P < 0.001 (Mann-Whitney)

For further checking C-containing PAM tolerance by Cas12a in human cells, As, Lb, and Ce Cas12a nucleases were investigated toward 82 endogenous target sites, with either TTTV, CTTV, TCTV, TTCV, CCTV, TCCV, or CCCV as the potential PAM (Fig. 3a). Target deep sequencing results showed that AsCas12a, LbCas12a, and Ce Cas12a orthologs efficiently modified all 9 target sites with the TTTV PAM (Fig. 3a). When substituting thymidine to cytidine at PAM position − 2, − 3, and − 4, Ce was in a very low activity at those C-containing PAMs (Fig. 3a), and the cleavage ratio of TTCV to TTTV achieved about 40% for LbCas12a and 25% for AsCas12a and the cleavage ratio of CTTV to TTTV also achieved about 23% for LbCas12a and 18% for AsCas12a (Fig. 3b). In contrast, the CeCas12a cleavage ratios of TTCV to TTTV and CTTV to TTTV were lower (about 20% and 8%) (Fig. 3b). These results demonstrated that CeCas12a presents a more rigorous recognition of TTTV PAM in human cells. Thus, this rigorous recognition might have the potential to hinder off-targeting at the C-containing PAM sites.

### Off-targeting was obstructed at C-containing PAM sites of CeCas12a in human cells

**Fig. 4:**
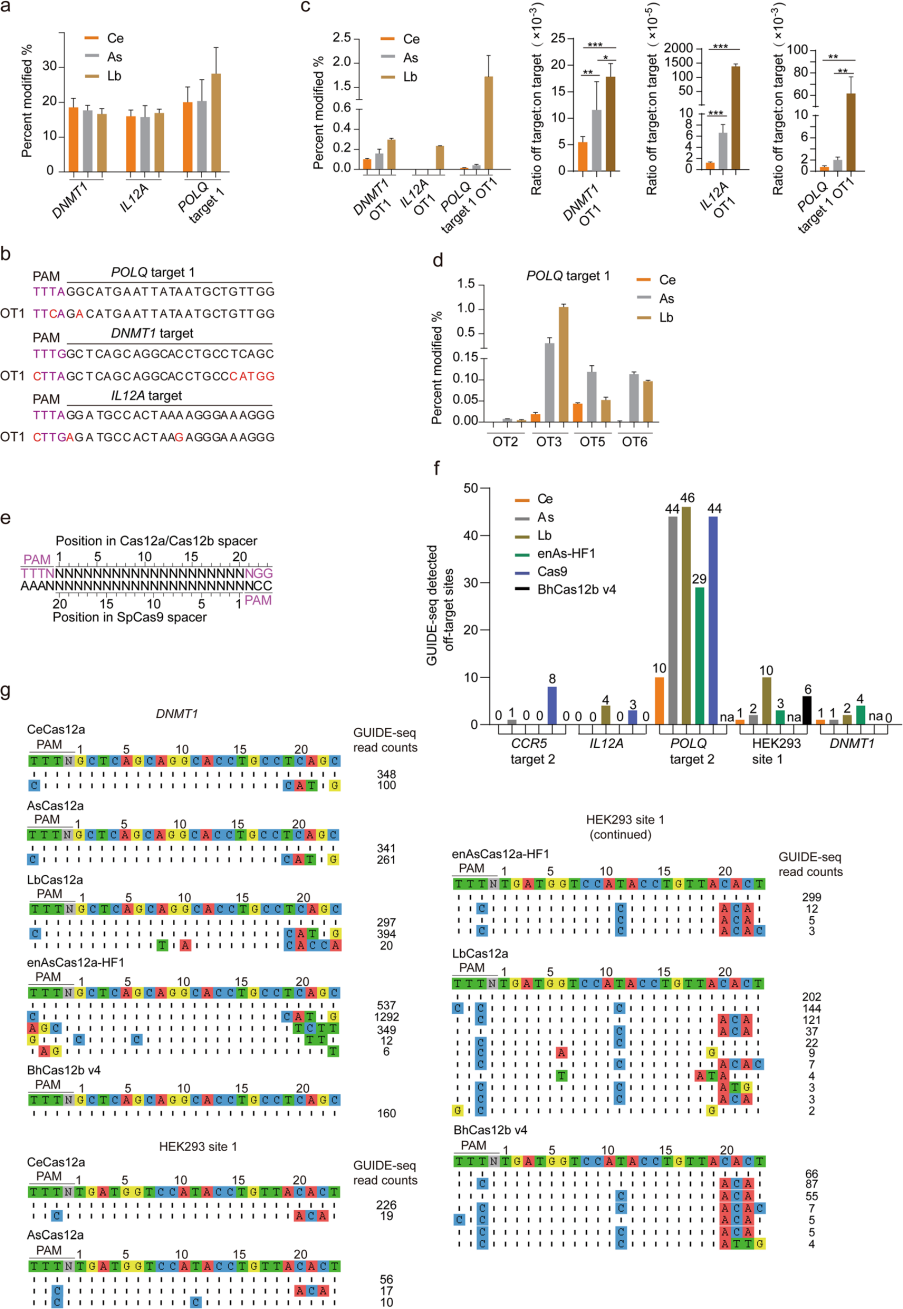
Assessment off-target effects of CRISPR-Cas nucleases. **a** Mean percent modification by Lb, As, and Ce at *POLQ*, *DNMT1*, and *IL12A* on-target sites. Percent modifications were determined by deep sequencing. Bars show mean ± s.e.m. for *n* = 3 transfected cell cultures. Indel mutation frequencies were indicated by the percentage of reads containing indels compared with the wild-type sequence reads. **b** Illustration of off-target sites at PAM sequence. Mismatches at PAM sequence and spacer are red. **c** Mean percent modification at off-target site 1 (OT1) (left), and specificity ratios of LbCas12a, AsCas12a, and CeCas12a with the *POLQ*, *DNMT1*, and *IL12A* crRNAs, plotted as the ratio of OT1 site activity to on-target activity (right). Data are shown as mean ± s.e.m. (*n* = 3). *P < 0.05, **P < 0.01, ***P < 0.001 (Mann-Whitney). **d** Mean percent modification by Lb, As, and Ce at off-target sites when targeting *POLQ* target 1. OT2, OT3, OT5, and OT6 were predicted by Cas-OFFinder, and mismatches are in spacer region (Additional file 1: Figure S13). **e** Matched target sites for Cas12a, Cas12b, and SpCas9 that share a common protospacer sequence. **f** Histograms illustrating the number of GUIDE-seq detected off-target sites for CeCas12a, AsCas12a, LbCas12a, enAsCas12a-HF1, spCas9, and BhCas12b v4. na, not assessed. **g** Off-target sites for CeCas12a, AsCas12a, LbCas12a, enAsCas12a-HF1, and BhCas12b v4 with DNMT1 and HEK293 site1 crRNAs, determined using GUIDE-seq in HEK293 cells. Mismatched positions are highlighted in color, and GUIDE-seq read counts are shown to the right of the on- or off-target sequences

AsCas12a and LbCas12a have been reported to introduce gene editing with high specificity in human cells [17, 18]. To further investigate the fidelity of CeCas12a, *DNMT1*, *POLQ*, *IL12A*, *HBB*, and *B2M* were selected as targets. Forty-one predicted off-target sites with C-containing PAMs and TTTN PAMs were both selected by using Cas-OFFinder [34] (Additional file 1: Figure S13A, B). After co-transfecting Cas12a plasmids and U6 crRNA fragments for 48 h, the target sites and off-target sites were amplified and went through targeted deep sequencing (Additional file 1: Figure S13A, B). The results confirmed that Cas12a nucleases were highly specific on genomic editing with only 8 sites of *DNMT1*, *POLQ*, and *IL12A* showing low off-target effects (Fig. 4a, c, d; Additional file 1: Figure S13A, B). And CeCas12a showed very low off-target rates as less than 0.05% in most of cases while maintaining the comparable cleavage efficiency to AsCas12a and LbCas12a (Fig. 4a, d; Additional file 1: Figure S13A, B). In detail, 4/8 predicted off-target sites (OT1, OT3, OT5, OT6) of *POLQ* were detected for all three Cas12a nucleases (Fig. 4c, d; Additional file 1: Figure S13A, B). For *DNMT1* and *IL12A*, off-target events also happened for all three Cas12a nucleases (Fig. 4c; Additional file 1: Figure S13A, B). Furthermore, 3 off-target sites (*POLQ* OT1, *DNMT1* OT1, *IL12A* OT1) turned out to have C-containing PAMs, which was TTCA, CTTA, and CTTG, respectively (Fig. 4b; Additional file 1: Figure S13), and CeCas12a had lower indel efficiencies comparable to AsCas12a and LbCas12a (Fig. 4c). For these three C-containing PAM off-target sites of AsCas12a, LbCas12a, and Ce Cas12a orthologs, the off-targeting efficiencies had big differences. The ratio of the off-target (OT1) to on-target exhibited significant differences, and CeCas12a presented the lower ratio, which was also consistent with the in vitro data that more tight stringency for recognizing non-canonical C-containing PAMs was revealed for CeCas12a (Fig. 4a–c; Additional file 1: Figure S14). For other off-target sites that mismatches happened in the spacer sequence region, CeCas12a was also revealed to be with less or similar off-target rates (Fig. 4d). To assess global off-target effects of CeCas12a and compare the genome-wide specificities of CRISPR-Cas nucleases including AsCas12a, enAsCas12a-HF1 [35], LbCas12a, spCas9, and BhCas12b v4 [36], we identified five endogenous target sites that contained overlapping target sites (except for HEK293 site 1 and *DNMT1*) for these CRISPR-Cas nucleases (Fig. 4e). And the genome-wide unbiased identification of double-stranded breaks (DSBs) enabled by sequencing (GUIDE-seq) method was used [37]. For two of five target sites evaluated (*CCR5* target 2, *IL12A*), no off-target activities were detected for CeCas12a (Fig. 4f). For other three target sites evaluated (*POLQ* target 2, *HEK293* site 1, and *DNMT1*), CeCas12a exhibited the similar or reduced number of off-target sites. And off-targeting that happened at PAM sequence was also detected by GUIDE-seq (Fig. 4f, g; Additional file 1: Figure S15) for all tested CRISPR-Cas nucleases, which is constant with the result of targeted deep sequencing.

Taken together, these results indicated the good characters of CeCas12a on the improvement of specific gene editing. Thus, CeCas12a could be another novel prominent Cas12a nuclease with improvements on off-targeting and comparable high cleavage efficiency to AsCas12a and LbCas12a in human cells. Also, the stringency or tolerance on non-canonical PAM sequences might be an important factor when considering off-targeting.

## Discussion

Cas12a was suggested to have low off-target rates in general with high cleave efficiency in mammalian cells and was considered as another kind of promising genomic editing tools. In this work, we report the identification of two novel Cas12a nucleases for genome editing in mammalian cells. One of them, named CeCas12a, was revealed to be more restrictive in vitro on PAM sequences than AsCas12a and LbCas12a. Certain permissions of other amino acids in the TTTV PAM sequence were somehow allowed for AsCas12a and LbCas12a. However, CeCas12a is much more restrictive on this permission. This feature provided the potential of decreasing off-targeting at the PAM region and attracted our interests.

The newly identified Cas12a nucleases were also tested for the efficiency of genome-editing ability in mammalian cells. Comparing to AsCas12a and LbCas12a, CeCas12a was identified to have comparable efficiency in editing multiple gene sites in 293T cells and BfCas12a only had comparable efficiency in limited target sites. The restriction on PAM sequences of CeCas12a was also confirmed in 293T cells. Cas12a nucleases such as AsCas12a and LbCas12a have very low off-target rates in general, and in many predicted off-target sites, off-targeting did not show up [17–19, 21]. It could be challenging to find other CRISPR-Cas proteins to have even lower off-target rates than the reported AsCas12a and LbCas12a. However, encouraged by the fact that CeCas12a has a more restricted PAM sequence requirement in vitro, we tested the off-targeting of CeCas12a in human 293T cells. From many off-target sites, several sites showed off-target effects in the PAM region and CeCas12a displayed lower off-target rates than other tested Cas12a. Thus, instead of increasing the scope of PAM to cover more potential target site, another direction of narrowing the scope of PAM for the sake of low off-targeting was indicated. And PAM stringency is not likely to limit the utility of CeCas12a for there is at least one TTTN PAM sequence per 25-bp nucleotides in human genome (Additional file 2: Table S2).

For other off-target sites where off-targeting only happened in the spacer sequence region, CeCas12a also shows similar or reduced off-targeting than other tested CRISPR-Cas nucleases in general. This could be due to the natural properties of CeCas12a, and more studies in the future could help us to understand off-targeting more and shape off-targeting more efficiently. Furthermore, CeCas12a was also confirmed to have the ability to truncate the pre-crRNA to the mature crRNA by itself and performing multigene editing in mammalian cells. Thus, CeCas12a could be another very useful tool in genome editing and disease therapy in the future.

Off-targeting is one of the important issues to hinder the clinical use of genome editing [37–39]. However, off-targeting is complicated and could happen at both the PAM region and the spacer region [19, 21]. For off-targeting at PAM region, the case of CeCas12a suggested that the more specific recognition of PAM sequences in vitro could be followed by the more specific PAM recognition in vivo. Thus, more usable genome-editing tools with low off-target rates could be developed by a novel means of restricting the PAM motif and could be discovered through the methodology of in vitro tested or screened easily at first.

Multiple other different ways have been developed to lower off-targeting by modifying the protein, the crRNA, the delivery, and etc. [40–43]. However, different orthologs of CRISPR-Cas proteins with lower off-target rates are less identified and CeCas12a could be such a good example, which points out that the in vitro property could be a very important guideline to find more useful gene-editing tools. Meanwhile, other ways such as modifying the protein, the crRNA, or the delivery might be easily combined with the newly identified CRISPR-Cas proteins such as CeCas12a. In the future, the combination of different ways to enable higher specificity of CRISPR-Cas nucleases for genome editing could be of great help for reaching the criteria of clinical usage.

## Conclusions

In conclusion, we have shown that the stringency of PAM recognition could be an important factor shaping off-targeting in gene editing which is demonstrated by a novel promising Cas12a ortholog. Two Cas12a orthologs, CeCas12a and BfCas12a, were identified to be active in human cells and able to perform multigene editing. Comparing to well-accepted AsCas12a and LbCas12a, CeCas12a was identified to have comparable efficiency in editing multiple gene sites and BfCas12a only had comparable efficiency in limited target sites. CeCas12a was shown to be more stringent for PAM recognition in vitro and in vivo than AsCas12a and LbCas12a followed by the lower off-targeting at the PAM region, and the off-targeting at other regions was also lower in general. Thus, CeCas12a represents a promising candidate due to its distinctive characteristics, and aiming at the stringency of PAM recognition could be an important clue to find more accurate gene-editing tools in future.

## Methods

### Plasmids for Cas12a or crRNA expression

A list and partial sequences of plasmids used in this study can be found in Table S4. AsCas12a, LbCas12a, and FnCas12a human expression plasmids were purchased from the non-profit plasmid repository Addgene (Addgene plasmids #69982, #69988, and #69976, respectively). CeCas12a and BfCas12a genes were synthesized and cloned into pcDNA3.1(+) for expression in human cells. The open reading frames of the nucleases were amplified by PCR (Phanta MAX Super-Fidelity DNA Polymerase, Vazyme) from their relevant human expression plasmids and inserted into the NcoI and XhoI sites of pET28a(+) for *E*. *coli* expression. Oligonucleotide duplexes corresponding to Cas12a orthologs and spacer sequences were PCR amplified and ligated into pU6-Fn-crRNA plasmids for U6 promoter-driven expression of crRNAs. The sequences of crRNA used in this study are also listed in Additional file 2: Table S3.

### Production of crRNAs

All crRNAs used in in vitro cleavage experiments were synthesized using the T7 High Yield RNA Transcription kit (Vazyme, TR101-02). ssDNA oligos (Additional file 2: Table S3) corresponding to the reverse complement of the target RNA sequence and a short T7 priming sequence were synthesized from TSING KE and annealed to obtain the template of the transcription reaction. T7 transcription was performed for 16 h, and then RNA was purified using the SanPrep Column PCR Product Purification Kit (Sangon Biotech).

### Purification of Cas12a proteins

The *E*. *coli* expression plasmids of the nucleases were transformed in *Escherichia coli* Rosseta cells. Protein expressions were inducted with 0.3 mM IPTG, 16 h. Cells were resuspended in lysis buffer (10 mM Tris-HCl (pH 8.0), 5 mM MgCl_2_, 200 mM NaCl, 5 mM imidazole, 0.1% Triton-100) and disrupted by Hydraulic Breaker. Cell debris and insoluble particles were removed by centrifugation at 12,000*g* at 4 °C. The lysate was filtered through 0.22-μm filters and applied to a nickel column, washed, and then eluted with a gradient of imidazole. Fractions containing protein of the expected size were pooled and applied onto a HiTrap Heparin HP column equilibrated with buffer L (10 mM Tris-HCl (pH 8.0), 5 mM MgCl_2_, 1 mM DTT, 200 mM NaCl). The protein was eluted with a linear gradient of 0–100% buffer H (10 mM Tris-HCl (pH 8.0), 5 mM MgCl_2_, 1 mM DTT, 1 M NaCl). The protein peaks were dialyzed with buffer L and concentrated. Then, the proteins can be either used directly for biochemical assays or frozen at − 80 °C.

### In vitro cleavage assay

Two hundred fifty-six kinds of linear dsDNA substrates were synthesized consisting of 4 randomized nucleotides upstream of the protospacer with overlap PCR. Cleavage in vitro was performed with purified proteins and corresponding crRNAs at 37 °C in cleavage buffer (20 mM HEPES-NaOH (pH 7.5), 100 mM KCl, 2 mM MgCl_2_, 1 mM DTT, and 5% glycerol) for 15 min. The cleavage reaction used 100 nM of synthesized crRNA and 200 ng of target DNA. Reactions were terminated with Protease K at 58 °C for 30 min and were run on 2% agarose gels.

### In vitro PAM identification assay

PAM library included 256 kinds of linear dsDNA substrates consisting of 4 randomized nucleotides upstream of 23 nt protospacer, and dsDNA substrates were mixed with each other at the same concentration. Each in vitro cleavage reaction consisted of 100 ng PAM library, 100 nM Cas12a RNP complex, 1 μL 10× CutSmart buffer (NEB), and water for a total volume of 10 μL. Each cleavage reaction was incubated at 37 °C and quenched at 0 min, 1 min, 2 min, 4 min, 6 min, 8 min, 10 min, 16 min, and 32 min. Reactions were purified with magnetic beads (VAHTS DNA Clean Beads, Vazyme), and 10 ng purified dsDNA was amplified with PCR over 20 total cycles using custom primers containing Illumina adaptors. The unmodified library amplicon was used as a negative control to determine initial PAM representation in the libraries. Purified PCR products were quantified followed by sequencing with a 75-cycle NextSeq kit (Illumina), and PAM regions were extracted, counted, and normalized to total reads for each sample.

### Analysis of PAM cleavage kinetics

Sequencing reads were filtered by Phred quality (≥ 30 for all of 256 kinds of PAM bases) and were analyzed using a custom Python script to estimate cleavage rates on each PAM for each of nuclease [35]. For each cleavage reaction, we calculated the depletion ratio (normalized read count in cleavage reaction)/(normalization read count in negative control) and then divided by the median depletion ratio of all VRRT sequences, which were not cleaved by Cas12a nucleases [19]. Each PAM (256 total) deletion ratio for each Cas12a nuclease over time fit to non-linear least squares to an exponential decay model (*y*(*t*) = *Ae*^−*kt*^, where *y*(*t*) is the normalized PAM count, *t* is the time (minutes), *A* is a constant, and *k* is the rate constant).

### Cell culture and transfection

HEK293T cells were cultured at 37 °C with 5% CO_2_ in DMEM supplemented with 10% heat-inactivated fetal bovine serum and 1% penicillin/streptomycin (all cell culture products were obtained from Gibco). Cells were seeded 1 day prior to transfection in 24- or 96-well plates at a density of approximately 1.2 × 105 cells per 24-well or 2.4 × 104 cells per 96-well and transfected at 50–80% confluency using Hieff Trans Liposomal Transfection Reagent (CAT:40802ES03, Yeasen, Shanghai). For indel analysis or EGFP disruption analysis in HEK293T cells, a total of 500 ng of Cas12a plasmid plus 200 ng crRNA plasmid was delivered per 24-well or 100 ng Cas12a plus 50 ng crRNA plasmid per 96-well.

### EGFP disruption assay

Human 293T cells were transfected with Cas12a expression plasmid, EGFP expression plasmid, and crRNA expression plasmid, or an U6 promoter-driven empty plasmid for the substitution of crRNA expression plasmid as a negative control. Forty-eight hours post-transfection, cells were analyzed on the CytoFLEX (Beckman Coulter). For each sample, transfections and flow cytometry measurements were performed in triplicate.

### T7E1 assays

Cells were collected after 48 h post-transfection for genomic DNA extraction using Animal Tissue Direct PCR Kit (CAT:10180ES70, Yeasen, Shanghai). The genomic region flanking the Cas12a targeting site of each gene was PCR amplified, and products were purified using SanPrep Column PCR Product Purification Kits (Sangon Biotech) following the manufacturer’s protocol. A total of ~ 200 ng purified PCR amplicons were denatured, reannealed, and digested with T7E1 (Vazyme). The reaction mixtures were run on 2% agarose gels after incubation for 20 min at 37 °C. The gels were imaged with ChemiDocTM XRS+ and analyzed according to strip intensities. Indel percentage was determined by the formula: 100 × (1 − sqrt(*b* + *c*)*/*(*a* + *b* + *c*)), where *a* is the integrated intensity of the undigested PCR product and *b* and *c* are the integrated intensities of the cleavage product.

### Targeted deep sequencing assays

Off-targets were predicted using the online website Cas-OFFinder (http://www.rgenome.net/cas-offinder/), and the relevant primers were designed. Each predicted off-target fragment is approximately 250 bp. These primers were used for PCR amplification amplified using corresponding genomic DNAs as templates. The purified PCR products were quantified by Infinite 200 (TECAN). Each 300 ng predicted off-target fragment was mixed and sent to Novogene for deep sequencing, and off-target analysis was performed according to the sequencing results. All of the above experiments were performed in three biological replicates.

### GUIDE-seq

GUIDE-seq experiments were performed as previously described [37]. Briefly, 100 pmol of the double-stranded oligodeoxynucleotide (dsODN) GUIDE-seq tag was transfected into 293T cells with CRISPR-Cas nuclease protein and guide RNA expression plasmids. On-target modification and GUIDE-seq tag integration percentages were assessed by using T7E1 assays (as described above) and restriction-fragment length polymorphisms (RFLP) assays, respectively. GUIDE-seq libraries were sequenced using an Illumina sequencer, and data was analyzed using guideseq v1.1 as described previously [37, 44].

### Statistics

Statistical significance was calculated using Mann-Whitney tests using GraphPad Prism version 8.0. The error bars in all figures show standard error of the mean (*n* = 3). *P* values are reported using GraphPad style: not significant (ns), *P* > 0.05; *, *P* < 0.05; **, *P* < 0.01; ***, *P* < 0.001.

## Supplementary information

**Additional file 1: Figure S1.** Diagram of Cas12a loci. **Figure S2.** Expression of Cas12a orthologs in *E. coli* cells. **Figure S3.** Substrates synthesis and cleavage assay. **Figure S4.** Extended gel image of Fig. 1a. **Figure S5.** DNA cleavage activity of BhCas12a in vitro. **Figure S6.** DNA cleavage activity of CsbCas12a in vitro. **Figure S7.** DNA cleavage activity of PrCas12a in vitro. **Figure S8.** Quantification of time-course in vitro cleavage activities of Cas12a orthologs. **Figure S9.** Multiple sequence alignment of Cas12a RuvC domains. **Figure S10.** Efficiencies of EGFP disruption mediated by As, Lb and Fn. **Figure S11.** Mutations induced by CeCas12a and BfCas12a. **Figure S12.** Gel images of Fig. 2f. Assessment of gene editing efficiencies with CeCas12a. **Figure S13.** Off-target effects of Cas12a-mediated gene editing in human cells. **Figure S14.** Off-target effects of Cas12a-mediated gene editing at C-containing PAM sites in human cells. **Figure S15.** Specificity analysis of matched CRISPR-Cas nuclease targets.

**Additional file 2: Table S1.** crRNA array locus of different Cas12a orthologs. **Table S2:** TTTN PAM distribution. **Table S3.** List of sequences used in study. **Table S4.** Oligonucleotides (oligos) for Cas12a gene synthesis.

**Additional file 3.** Sequencing data for PAM identification and PAM cleavage kinetics analysis.

**Additional file 4.** Review history.

## Data Availability

The sequences of expression cassettes used in this paper are listed in Additional file 2. Raw sequencing reads are available at the Gene Expression Omnibus under accession GSE146420 [45]. All materials described in this study are freely available upon request.
