# Supplementary material for: A Cas12a ortholog with stringent PAM recognition followed by low off-target editing rates for genome editing
Source: Genome Biol. 2020 Mar 25;21:78. doi: 10.1186/s13059-020-01989-2 (PMC7093978; doi:10.1186/s13059-020-01989-2)

**Additional file 1: Figure S1.** **Diagram of Cas12a** **loci.** **(A)** Schematic of Cas12a orthologs. **(B)** Schematic representation of eight crRNA direct repeat structures. The difference among these eight Cas12a family members is shown in shadow. Among of them, Ce, Bf, Fn/Pb, Pe, Li/Lb2, Pc share the same direct repeat (DR).


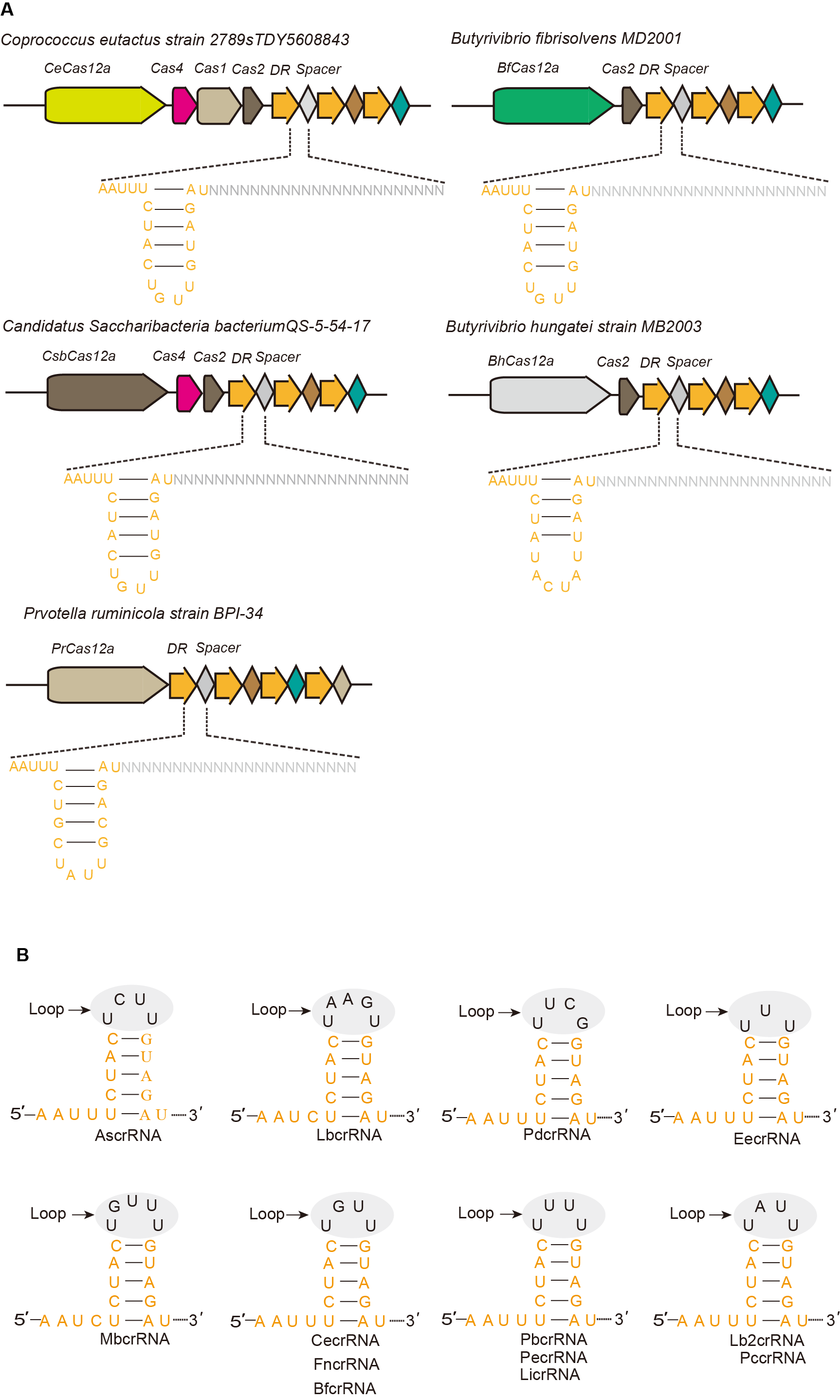


**Additional file 1: Figure S2.** Substrates synthesis and cleavage assay. **(A)** Schematic diagram of substrates synthesis with over-lap PCR. **(B)** Cleavage assay with Cas12a-crRNA complex in vitro.

**
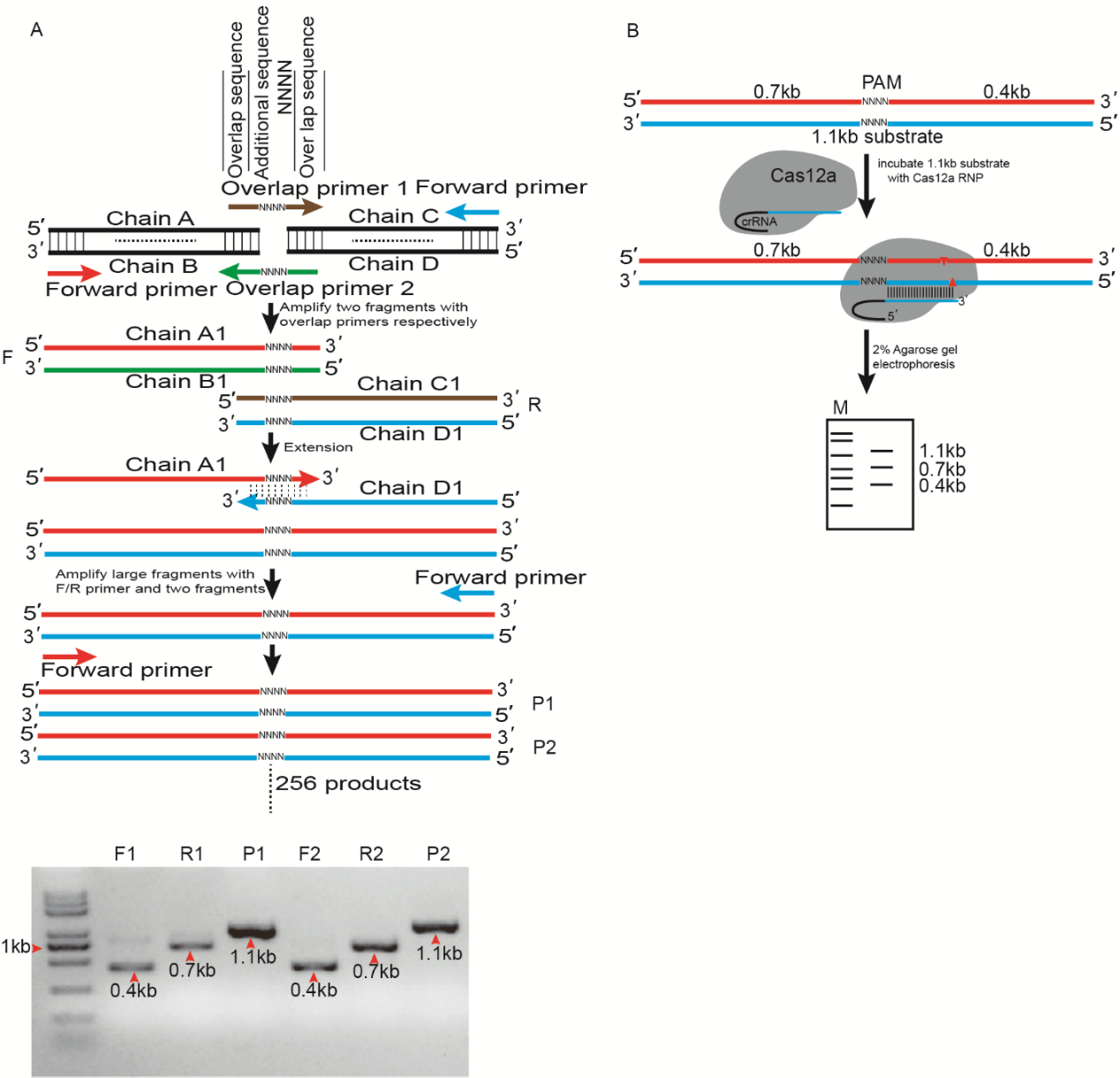
**

**Additional file 1: Figure S3. Expression of Cas12a orthologs in E. coli cells.**

**
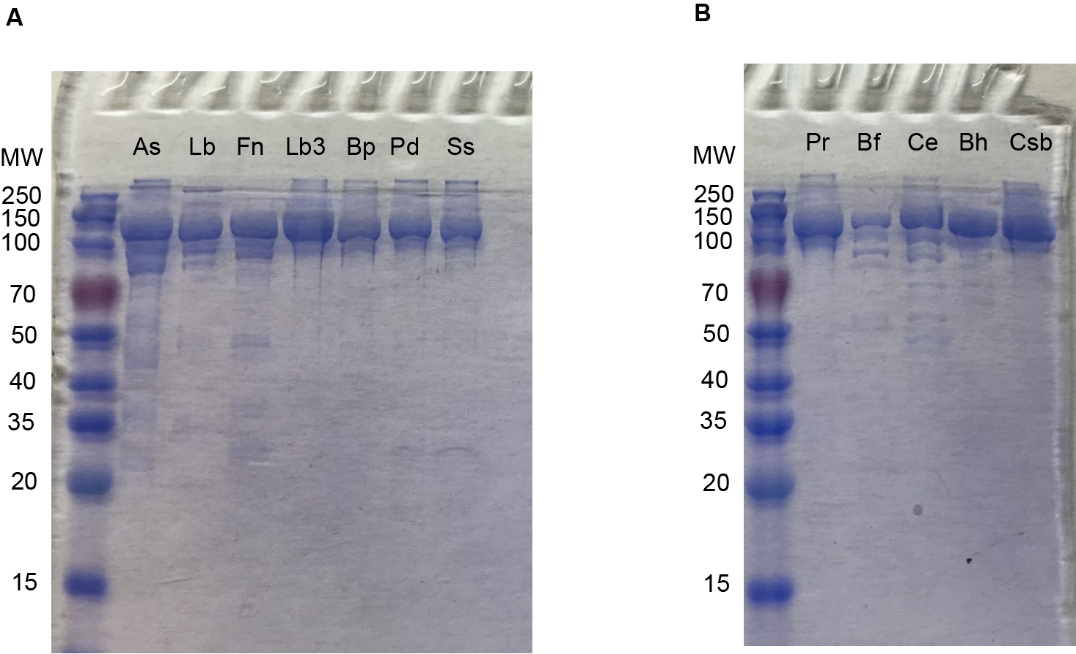
**

**Additional file 1: Figure S4.** Extended gel image of figure 1A. DNA cleavage activity of CeCas12a **(A)** and BfCas12a **(B)** in vitro. The Cas12a-crRNA complex (100 nM) was incubated at 37℃ for 10 min with DNA substrates consisting of 4 randomized nucleotides downstream of the same protospacer.

**
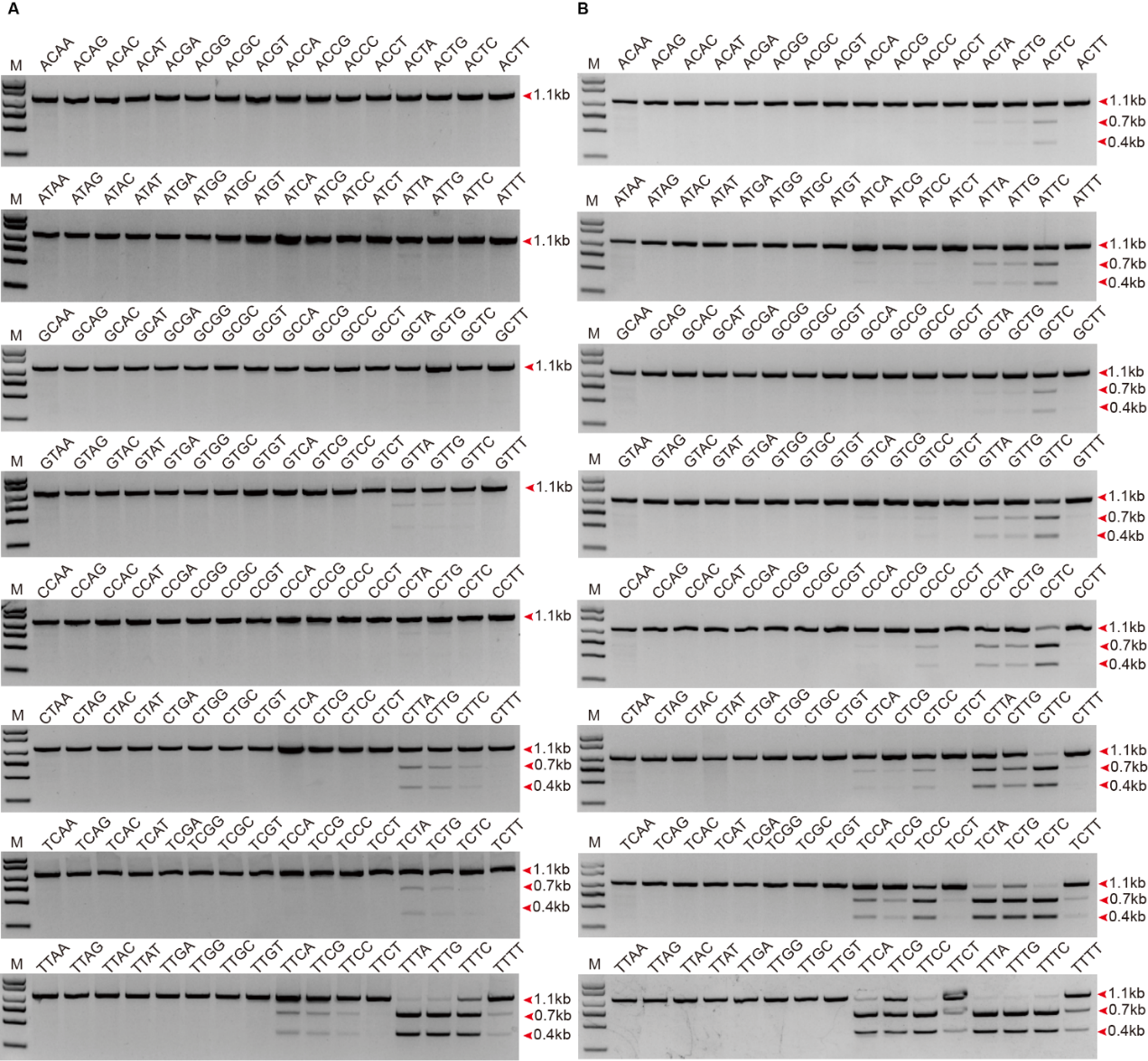
**

**Additional file 1: Figure S5.** DNA cleavage activity of BhCas12a in vitro. The Cas12a-crRNA complex (100 nM) was incubated at 37℃ for 10 min with DNA substrates consisting of 4 randomized nucleotides downstream of the same protospacer.

**
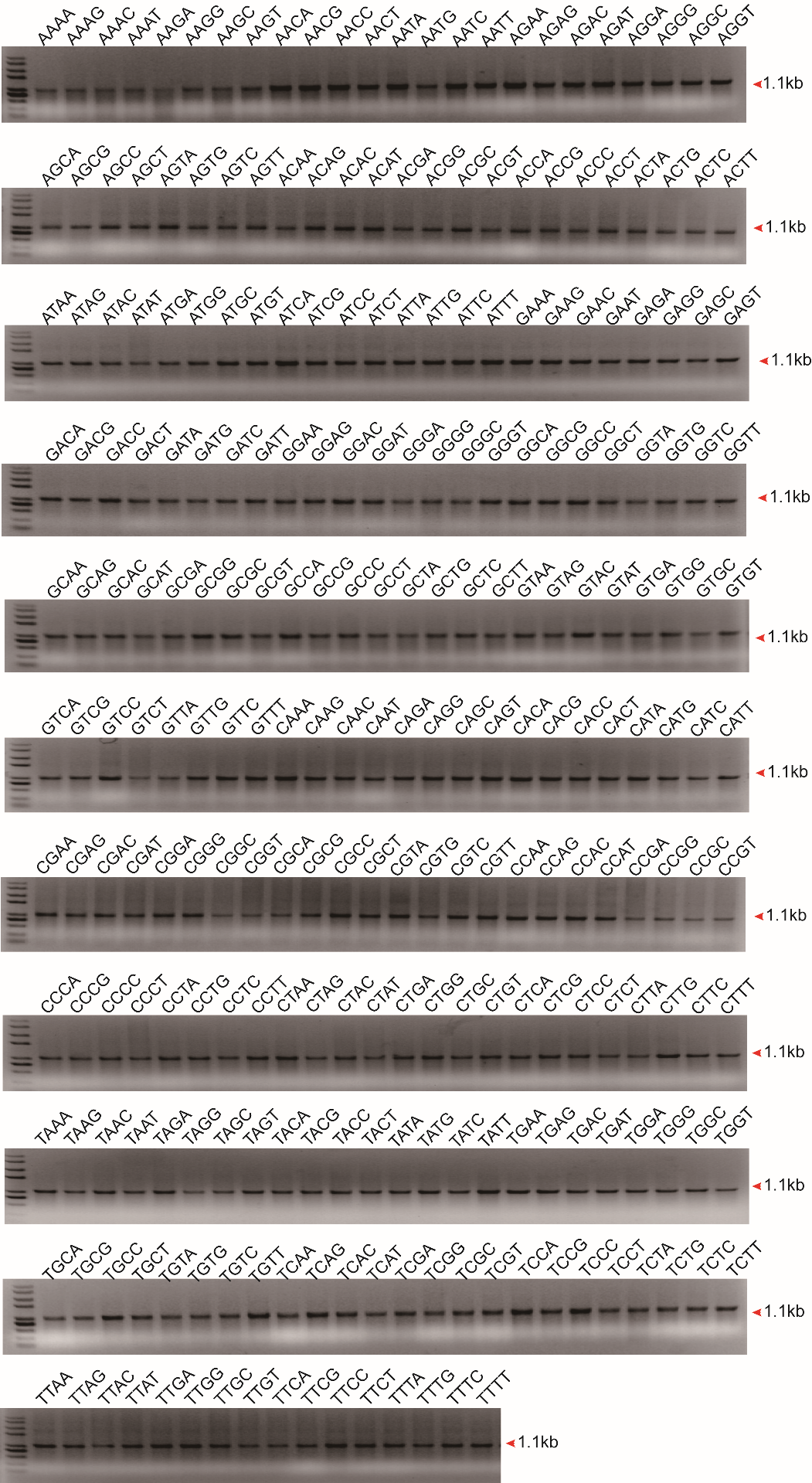
**

**Additional file 1: Figure S6.** DNA cleavage activity of CsbCas12a in vitro. The Cas12a-crRNA complex (100 nM) was incubated at 37℃ for 10 min with DNA substrates consisting of 4 randomized nucleotides downstream of the same protospacer.

**
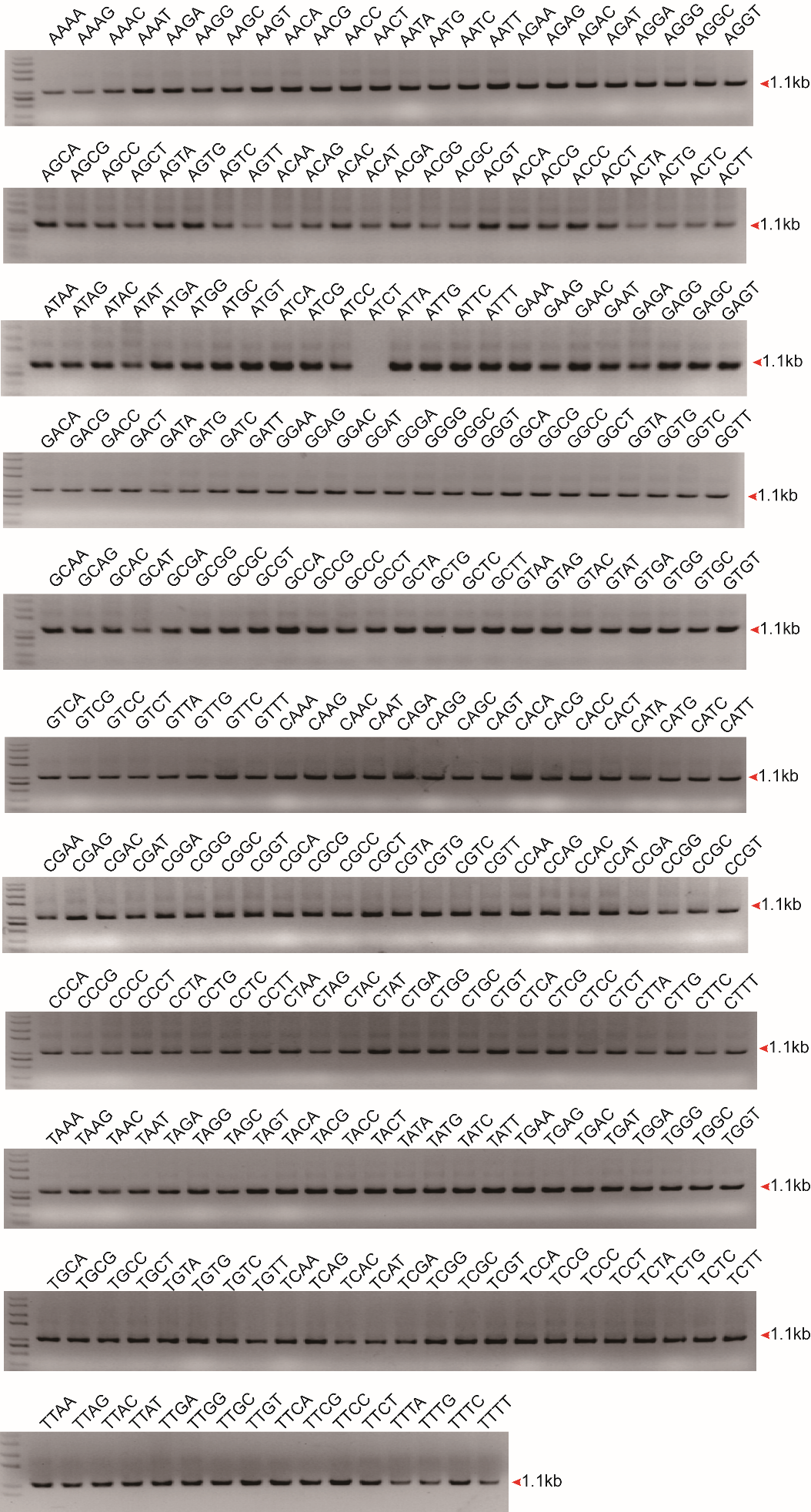
**

**Additional file 1: Figure S7.** DNA cleavage activity of PrCas12a in vitro. The Cas12a-crRNA complex (100 nM) was incubated at 37℃ for 10 min with DNA substrates consisting of 4 randomized nucleotides downstream of the same protospacer.

**
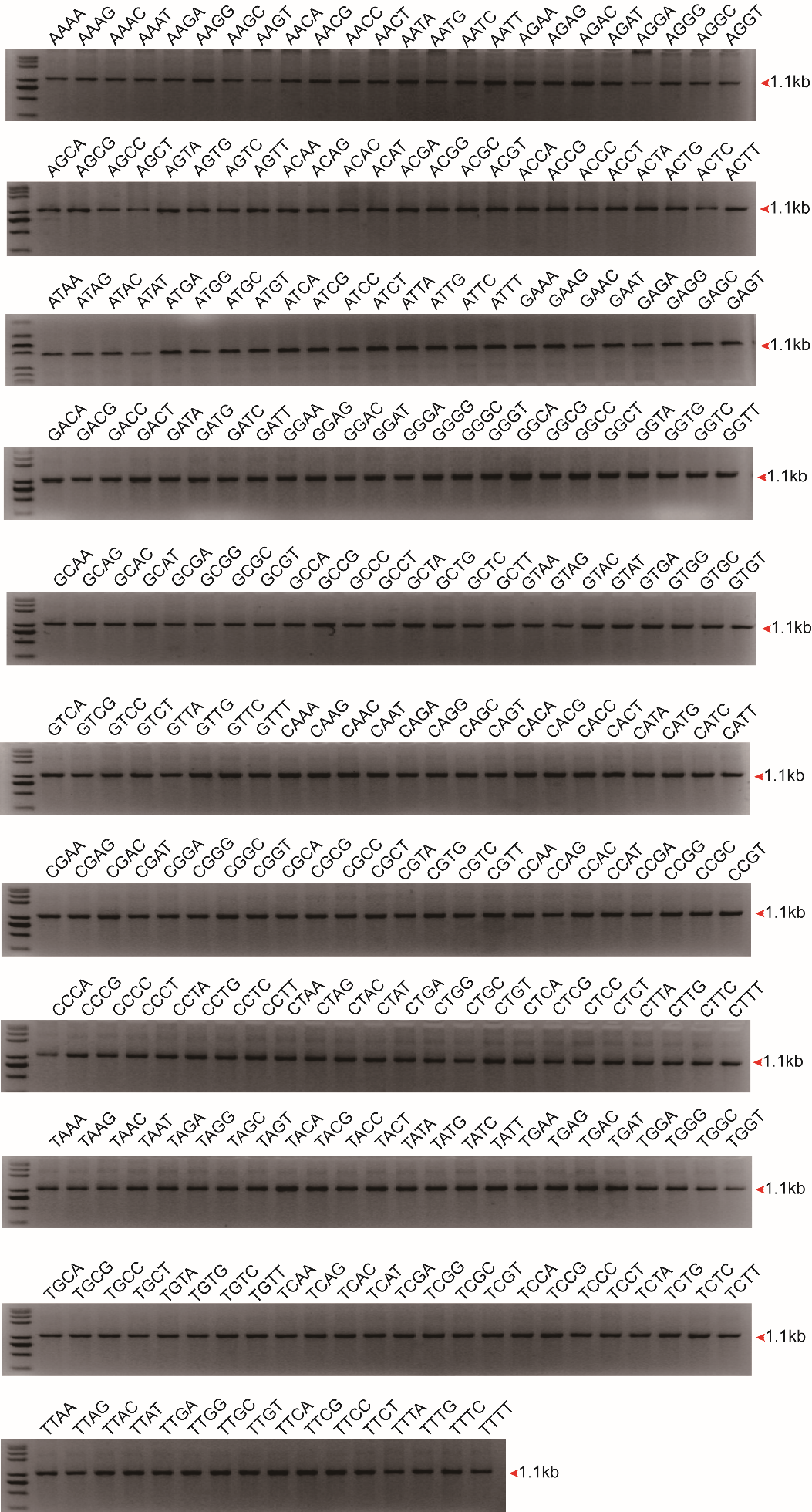
**

**Additional file 1: Figure S8.** Quantification of time-course in vitro cleavage activities of Cas12a orthologs. **(A)** Cas12a orthologs cleavage activities in vitro. The Cas12a-crRNA complex (100 nM) was incubated at 37℃ for 10 min with 300 ng DNA substrates. (**B**) The preferences of Cas12a orthologs toward non-canonical C-containing PAMs in vitro. The Cas12a-crRNA complex (100 nM) was incubated at 37°C for 10 min with 300 ng DNA substrates with the different PAMs (TTTA, TCTA, TTCA, TCCA, CTTA, CTCA, CCTA, CCCA), respectively.


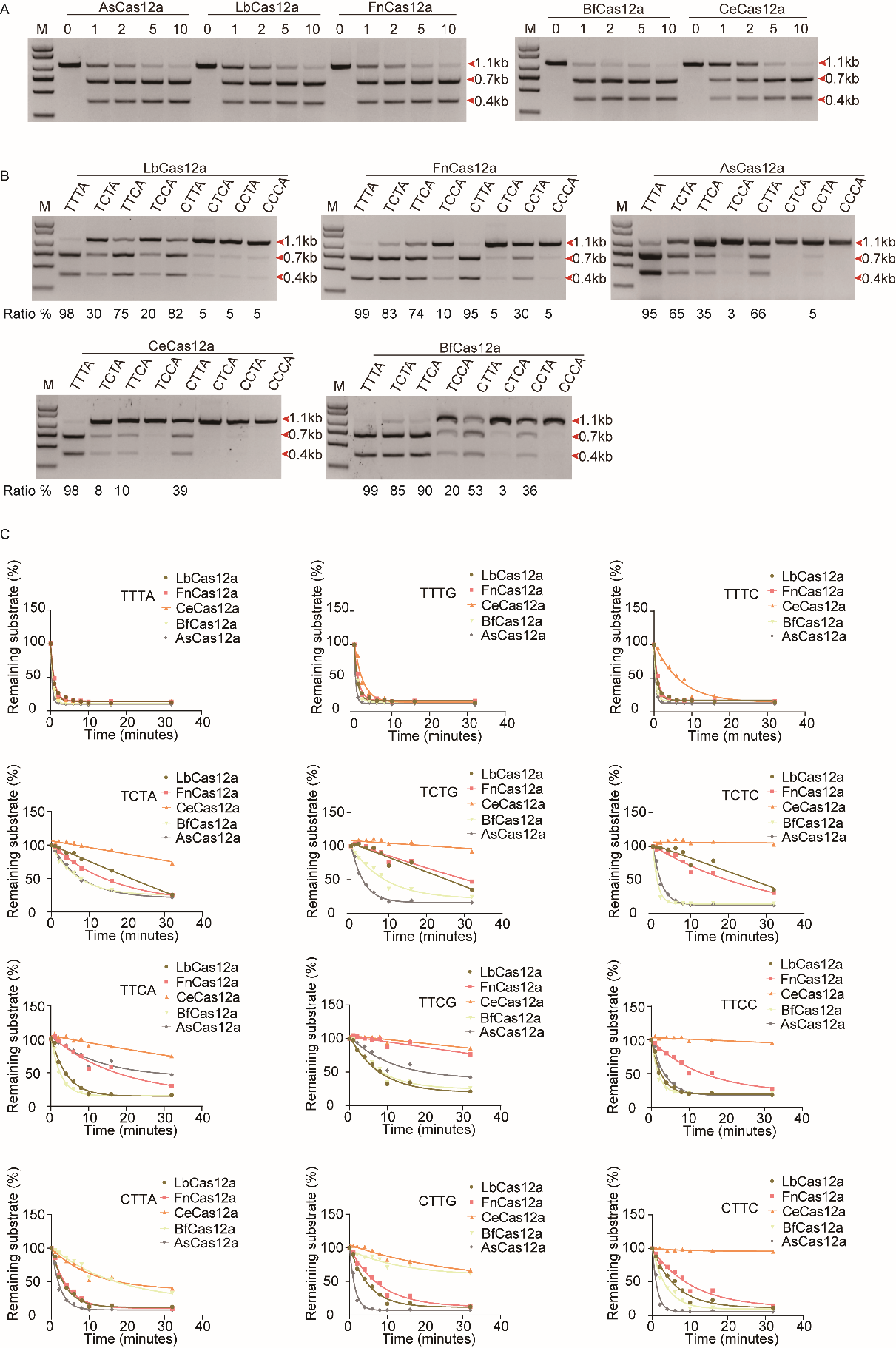


**Additional file 1: Figure S9.** Multiple sequence alignment of Cas12a RuvC domains. **(A)** The key residues of catalytic residues are indicated by triangles, the figure was prepared using Clustal Omega (<http://www.ebi.ac.uk/Tools/msa/clustalo>). **(B)** Catalytic residues in the RuvC domains are required for DNA cleavage, mutation of the RuvC catalytic residues of CeCas12a (D880A and E975A), BfCas12a (D834A and E925A), AsCas12a (D908A and E994A), LbCas12a (D832A and E925A), and FnCas12a (D917A and E1006A) prevent DNA cleavage in vitro. **(C, D)** Abolished gene editing by mutating catalytic residues, EGFP disruption assay **(C)** and T7E1 assay for endogenous site *IL12A* of wild-type Cas12a and catalytic residues mutation variants (D).


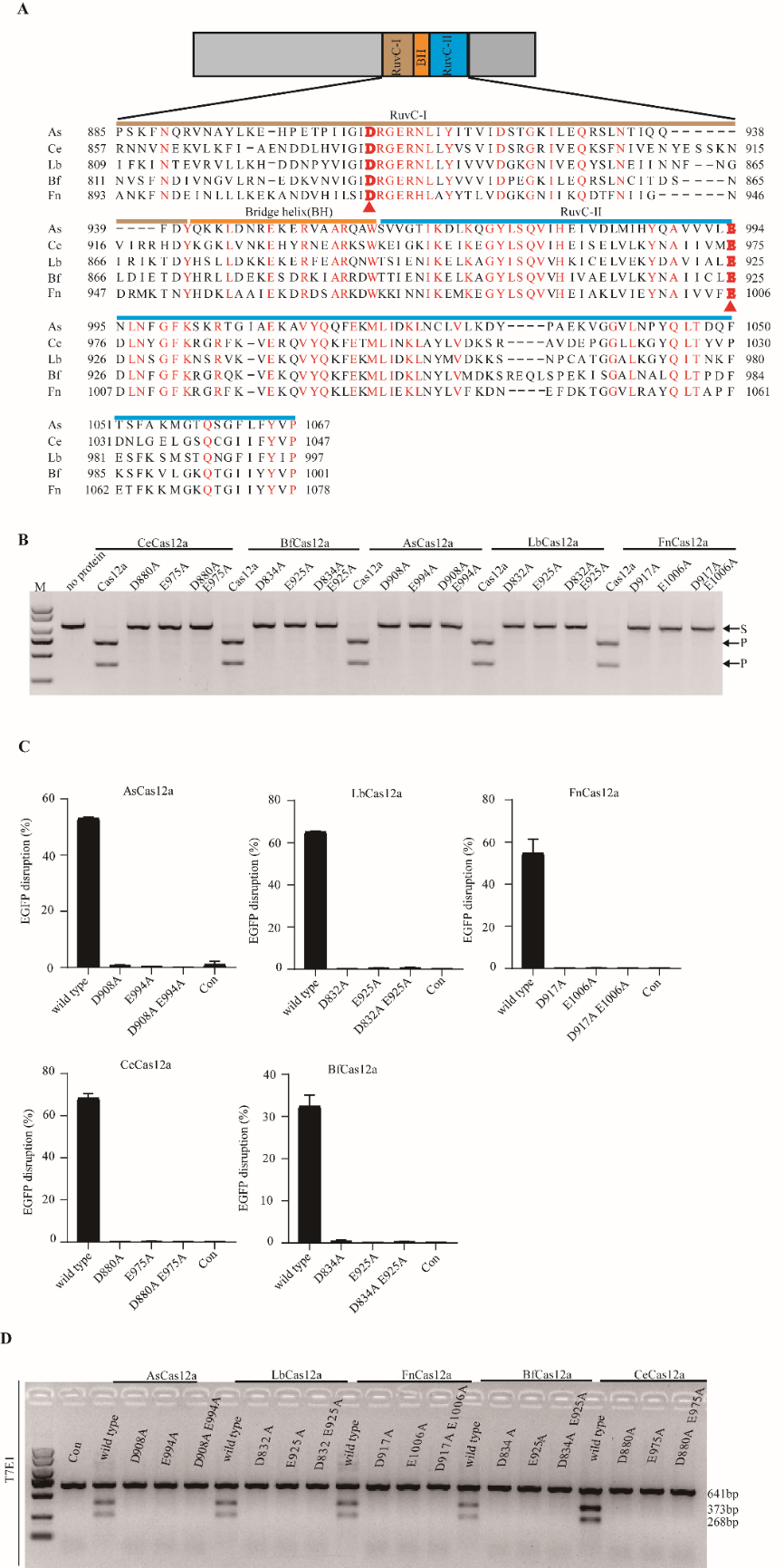


**Additional file 1: Figure S10.** Efficiencies of EGFP disruption mediated by As, Lb and Fn. Efficiencies of EGFP disruption in human cells mediated by As, Lb, Fn and crRNAs bearing variable length complementarity regions for the target site of *EGFP* in human cells. Error bars indicate standard errors of means (s.e.m.), n=3. ****P* <0.001.

**
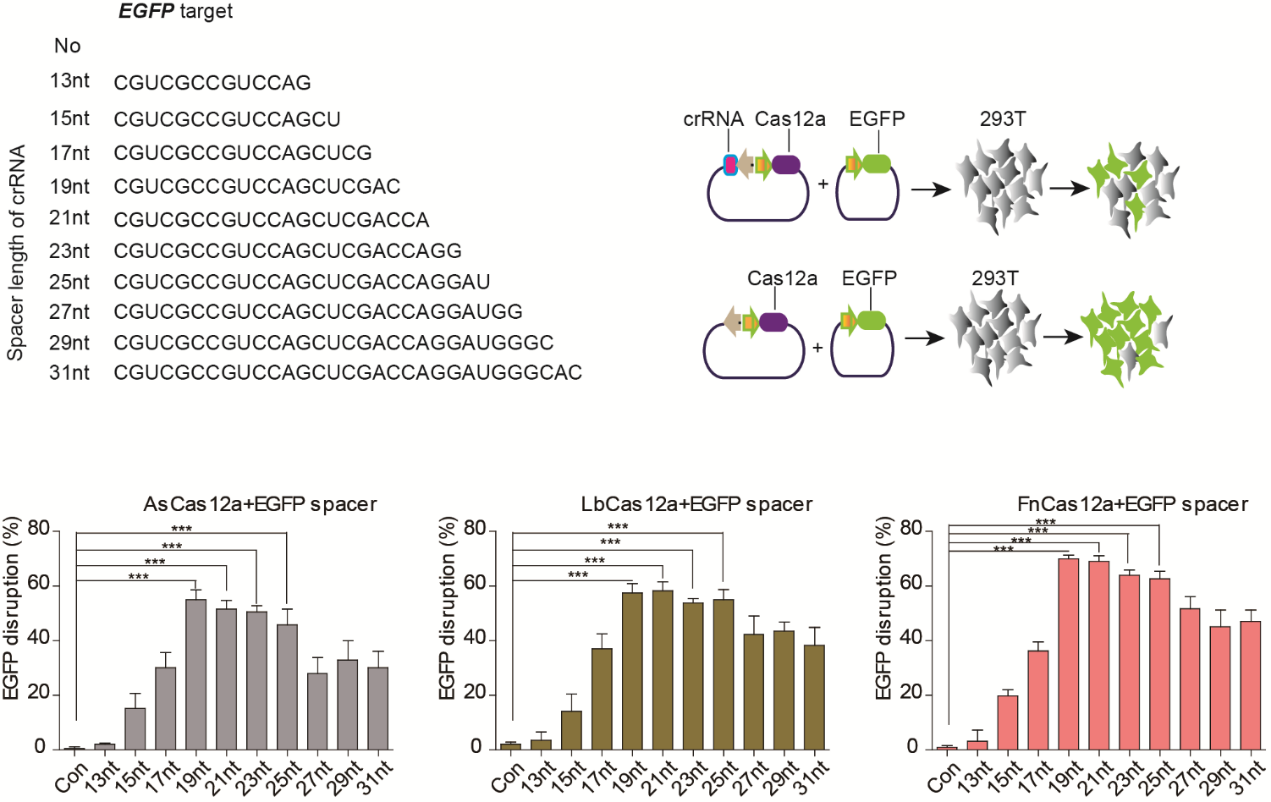
**

**Additional file1: Figure S11.** Mutations induced by Ce and Bf. Sequencing reads show representative mutations of BfCas12a and CeCas12a mediated gene editing with their own crRNA in *B2M*, *CTLA4*, *IL12A*, *HBB*, *VEGFA* site 2, *TRBC*, *DNMT1* and *POLQ* loci. Dashes represent the DNA deletions. The number at the right side of each sequence is the length of indel (−, deletion).

**
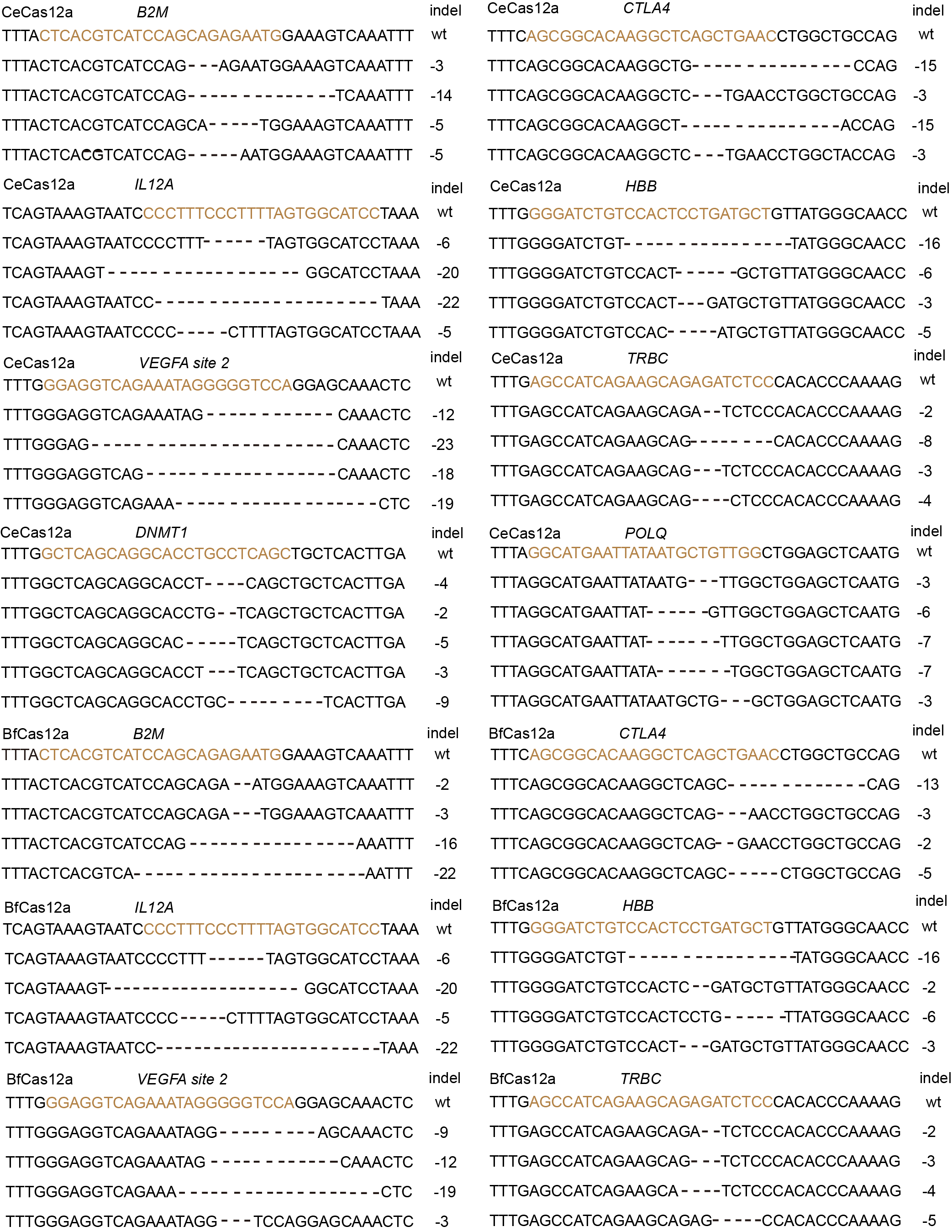
**

**Additional file 1: Figure S12.** Gel image of figure 2F. Assessment of gene editing efficiencies with CeCas12a. Activities assessed by T7E1 assay. Replicates represented transfected cell cultures times (n=3), Indel percentage was determined by the formula, 100 × (1 - sqrt(*b* + *c*)*/*(*a* + *b* + *c*)), where *a* is the integrated intensity of the undigested PCR product; *b* and *c* are the integrated intensities of the cleavage product.


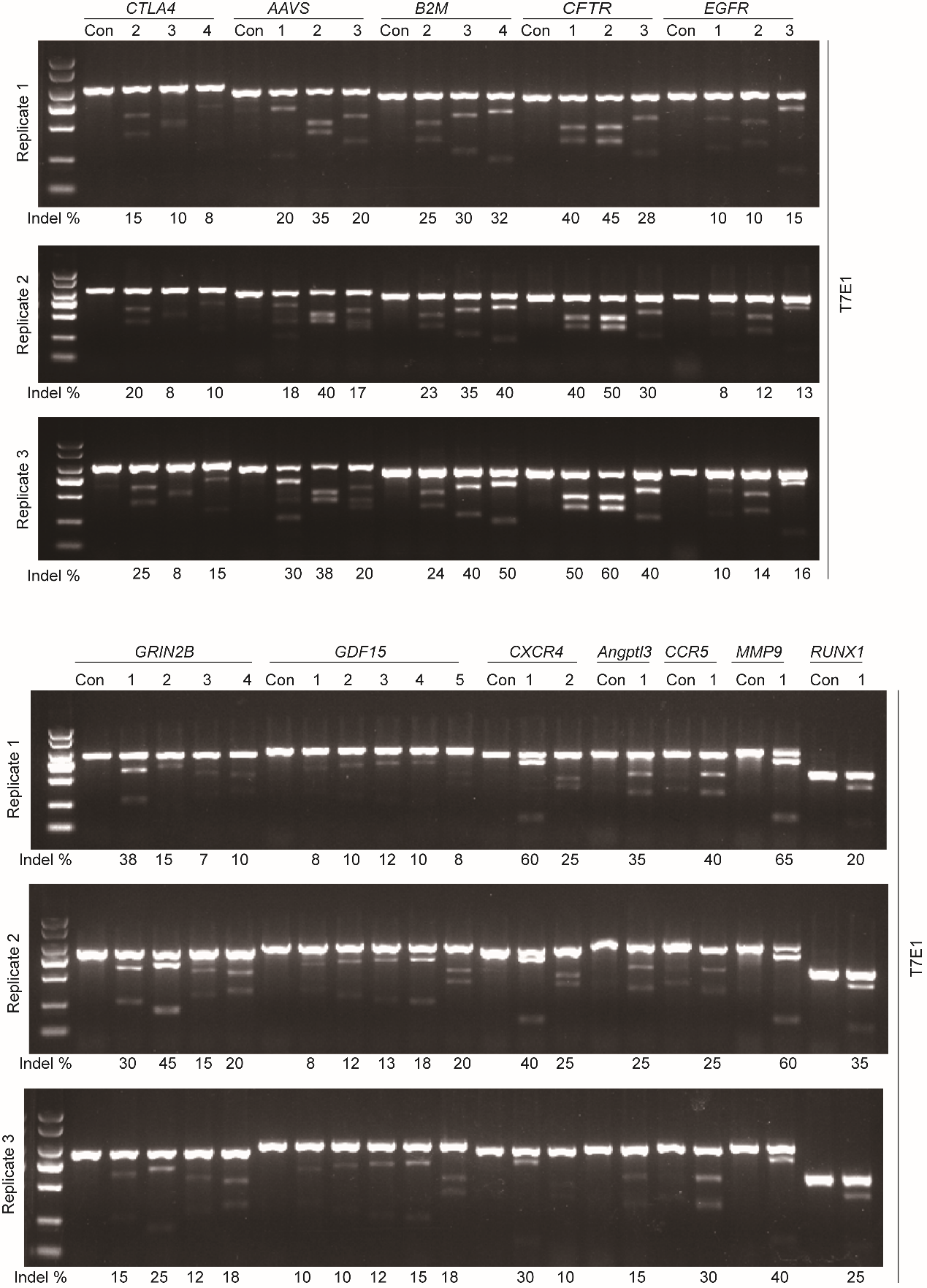


**Additional file 1: Figure S13.** Off-target effects of Cas12a-mediated gene editing in human cells. The off-target sites were predicted with online software (http://www.rgenome.net/cas-offinder/). Off-targets of *DNMT1*, *HBB* **(A)** and *IL12A*, *POLQ*, *B2M* **(B)** target sequence at different genes were investigated by deep sequencing.

**
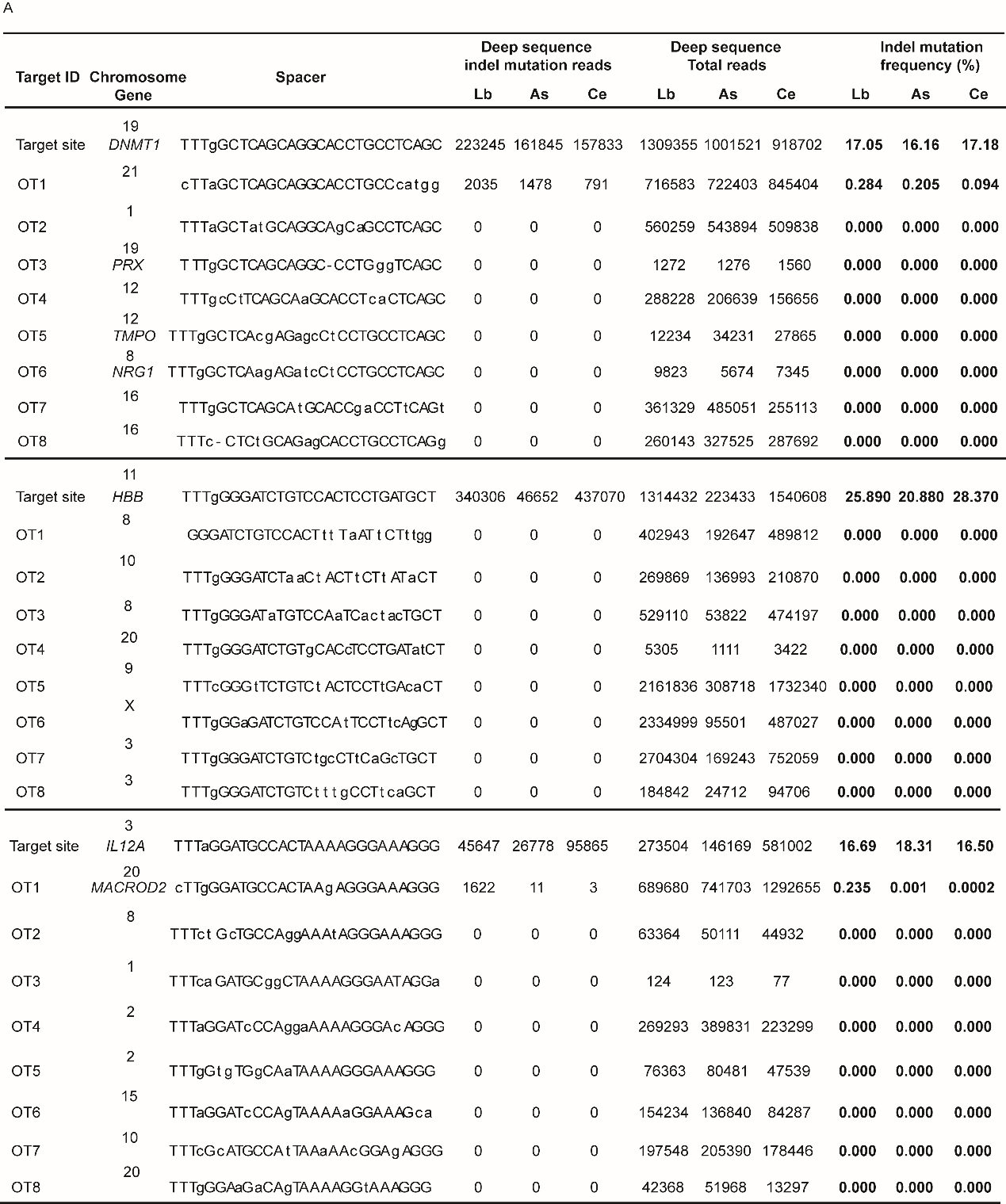
**

**
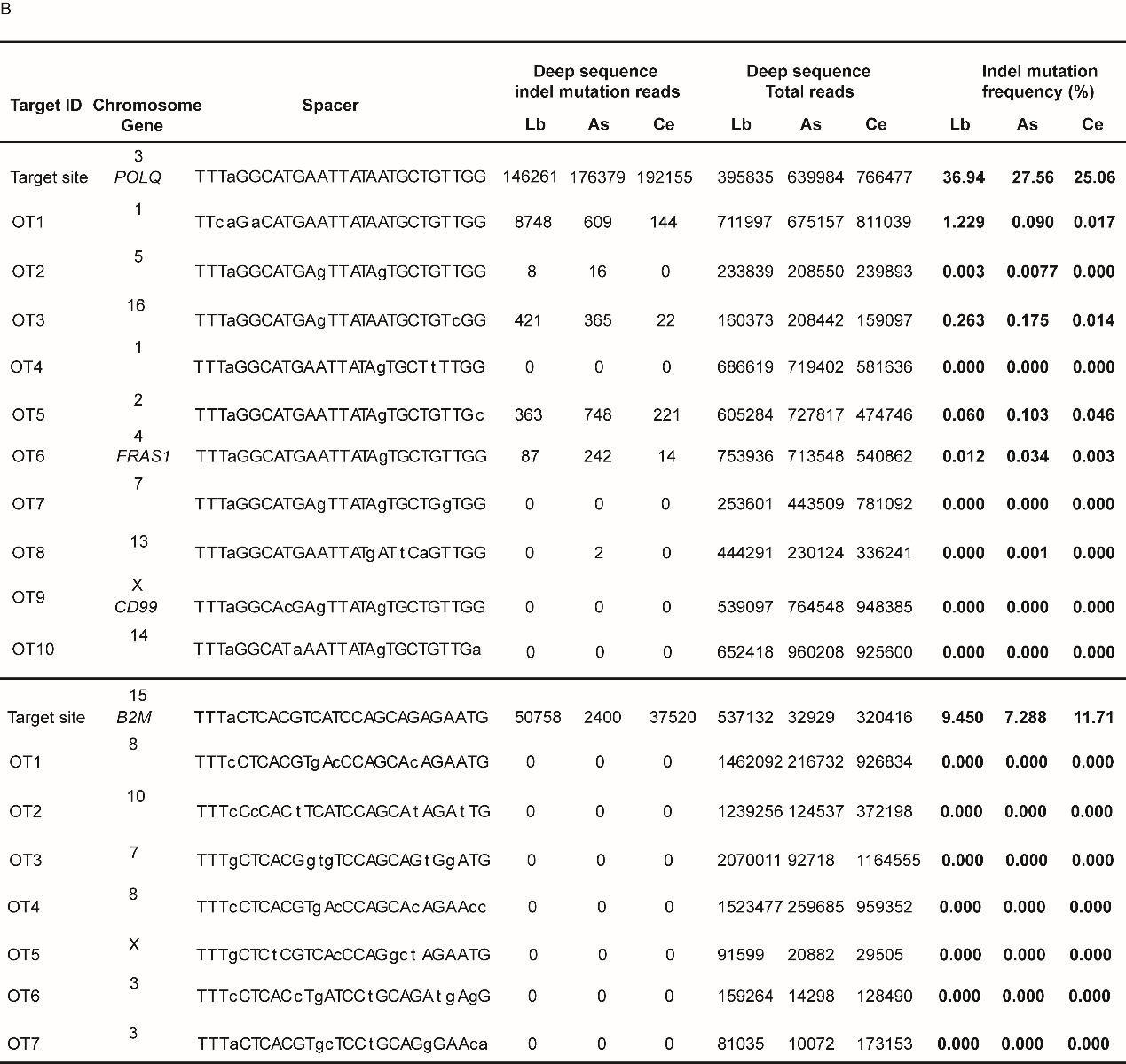
**

**Additional file 1: Figure S14.** Off-target effects of Cas12a-mediated gene editing at C-containing PAM sites in human cells. Sequencing reads show representative mutations of Cas12a mediated gene editing in *POLQ* OT1, *DNMT1* OT 1, *IL12A* OT1 loci. C-containing PAMs are shown in purple. Dashes represent the DNA deletions. Deep sequence reads and Percent modified are shown in red. Total percent modified are labeled with boxes in different color.

**
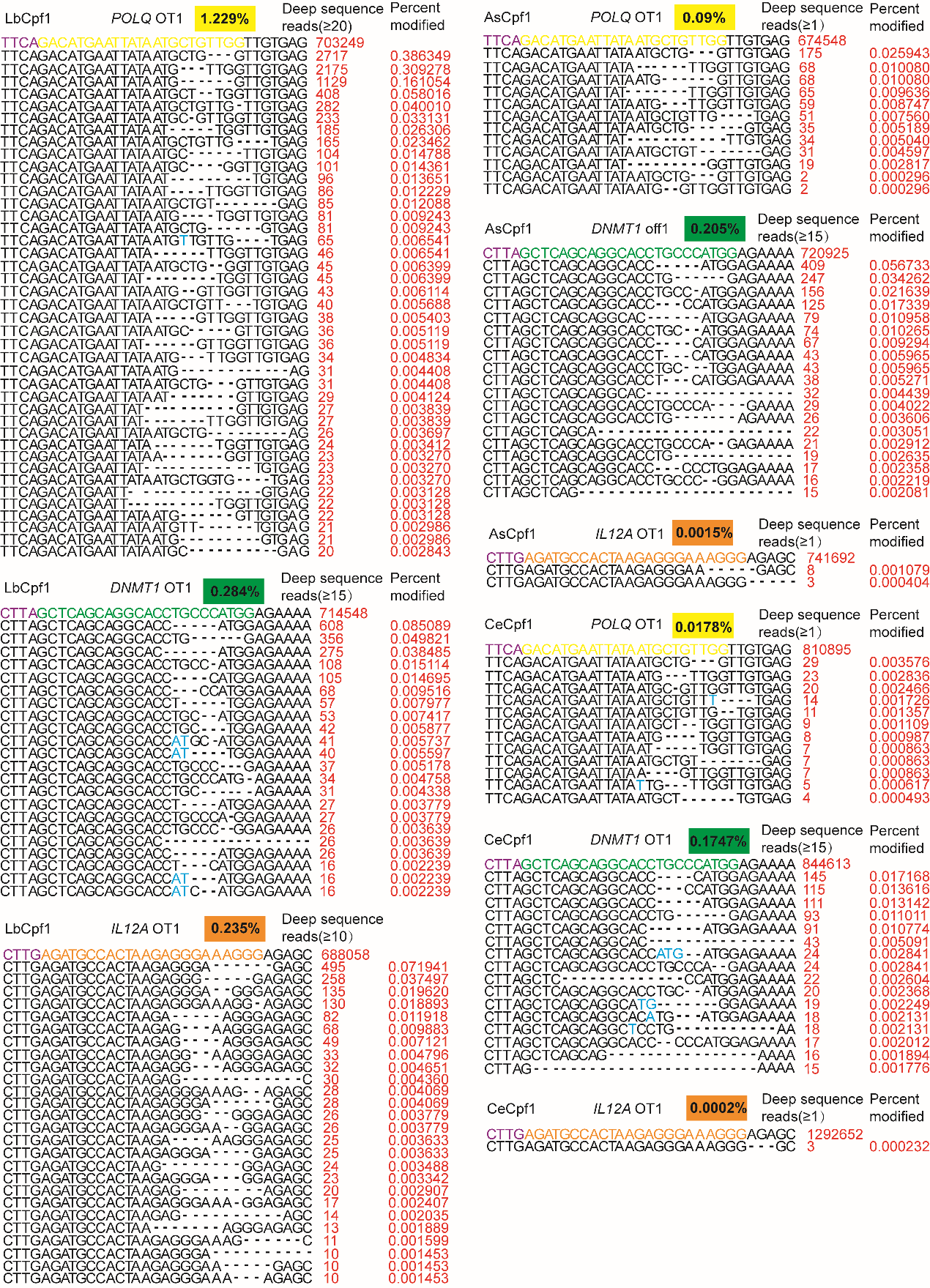
**

**Additional file 1: Figure S15.** Specificity analysis of matched CRISPR-Cas nuclease targets. GUIDE-seq analysis of detected off-targets in Fig. 4F (*CCR5*, *POLQ* target 2, *IL12A*). Matched target sites for Cas12a, Cas12b and SpCas9 that share a common protospacer sequence and mismatches to guide sequence are highlighted.


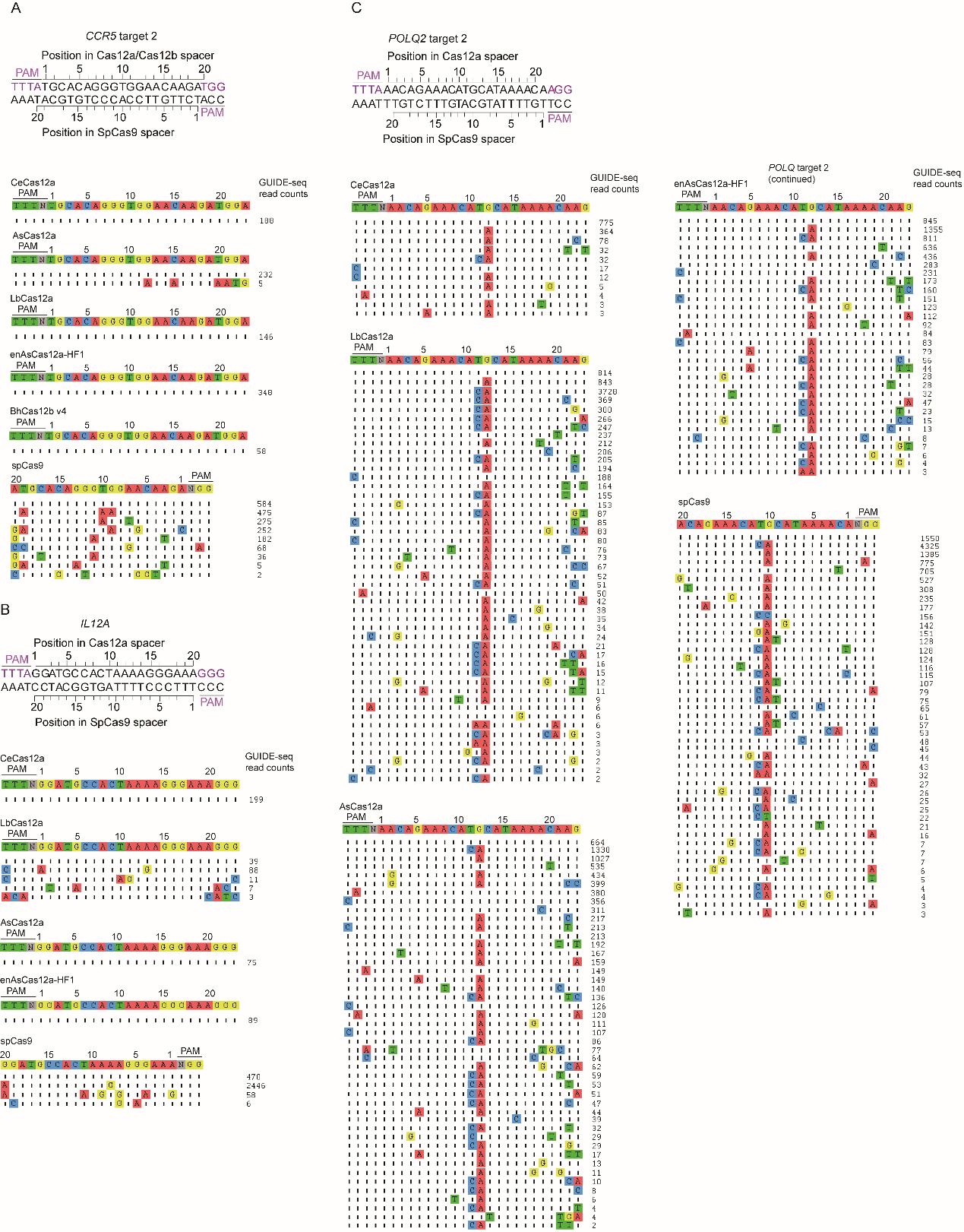

Supplement: Supplementary file 1 — Additional file 1: Figure S1. Diagram of Cas12a loci. Figure S2. Expression of Cas12a orthologs in E. coli cells. Figure S3. Substrates synthesis and cleavage assay. Figure S4. Extended gel image of Fig. 1a. Figure S5. DNA cleavage activity of BhCas12a in vitro. Figure S6. DNA cleavage activity of CsbCas12a in vitro. Figure S7. DNA cleavage activity of PrCas12a in vitro. Figure S8. Quantification of time-course in vitro cleavage activities of Cas12a orthologs. Figure S9. Multiple sequence alignment of Cas12a RuvC domains. Figure S10. Efficiencies of EGFP disruption mediated by As, Lb and Fn. Figure S11. Mutations induced by CeCas12a and BfCas12a. Figure S12. Gel images of Fig. 2f. Assessment of gene editing efficiencies with CeCas12a. Figure S13. Off-target effects of Cas12a-mediated gene editing in human cells. Figure S14. Off-target effects of Cas12a-mediated gene editing at C-containing PAM sites in human cells. Figure S15. Specificity analysis of matched CRISPR-Cas nuclease targets. [file 13059_2020_1989_MOESM1_ESM.docx]
