# Supplementary material for: A Cas12a ortholog with stringent PAM recognition followed by low off-target editing rates for genome editing
Source: Genome Biol. 2020 Mar 25;21:78. doi: 10.1186/s13059-020-01989-2 (PMC7093978; doi:10.1186/s13059-020-01989-2)
Supplement: Supplementary file 2 — Additional file 2: Table S1. crRNA array locus of different Cas12a orthologs. Table S2: TTTN PAM distribution. Table S3. List of sequences used in study. Table S4. Oligonucleotides (oligos) for Cas12a gene synthesis. [file 13059_2020_1989_MOESM2_ESM.docx]

**Additional file 2:** **Table S1.** crRNA array locus of different Cas12a orthologs

| **Cas12a strain** | **crRNA array locus of different Cas12a orthologs** |
| --- | --- |
| Coprococcus eutactus strain 2789STDY5608843 | AATTTGGAGTTGAGTAACCTTAAATAATTTCTACTGTTGTAGATCCCAACTTTGAATTGCACAATTTCATAGTTGAGTAACCTTAAATAATTTCTACTGTTGTAGATATGAACACCTCTTTTGTAACCATGATAGTTGAGTAACCTTAAATAATTTCTACTGTTGTAGATTTGCATTCATGCCCTGTGCAAGCTGGGTTGAGTAACCTTAAATAATTTCTACTGTTGTAGATAAAGCACTGGAAAAGCCCGGTATGAAGTTGAGTAACCTTAAATAATTTCTACTGTTGTAGATAACGTAGGTCACAAGGTGTTCCGTTAGGTTGAGTAACCTTAAATAATTTCTACTGTTGTAGATTGCCAAGATTGAAGCCCGGTCGAGAAAGTTGAGTAACCTTAAATAATTTCTACTGTTGTAGATGATCATCTGTTGACATTCTGTTAATCTCGGGTTGAGTAACCTTAAATAATTTCTACTGTTGTAGATTCTAAGGATATAGATAAGCTACCAACGTTGAGTAACCTTAAATAATTTCTACTGTTGTAGATA |
| Butyrivibrio hungatei strain MB2003 plasmid pNP144 | GTCTAAGACTTAAAGATAATTTCTATACTATTAGATCCGCTTCTTGTGTATGCTTCAATCACTTGTCTAAGACTTAAAGATAATTTCTATACTATTAGATGATATTGTGGTGACTTTGCAGGAATGAGTCTAAGACTTAAAGATAATTTCTATACTATTAGATATAGAATTTAAGGGTGTCAATTTTTTTTGTCTAAGACTTAAAGATAATTTCTATACTATTAGATGTGTTTCACCTCCCTTCCATATGGTTGTCTAAGACTTAAAGATAATTTCTATACTATTAGATAGCAATCGCTTAATGGTCGCATGGGTCGTCTAAGACTTAAAGATAATTTCTATACTATTAGATTTATATCGGGGCGATATGAATTCAGTAAGTCTAAGACTTAAAGATAATTTCTATACTATTAGATATGTACCTATGTATAAACCTGGTGAAAGTCTAAGACTTAAAGATAATTTCTATACTATTAGAT |
| Butyrivibrio fibrisolvens MD2001 G635DRAFT_scaffold00009.9_C | CCAAGAACCTATAGATAATTTCTACTGTTGTAGATTTCGATGATCTTAACTACCTGGTCGCGAGCCAAGAACCTATAGATAATTTCTACTGTTGTAGATGGCTGGCCCATCATATCAAGAACGGGATGCCAAAAACCTATAGATAATTTCTACTGTTGTAGATTATAGGAGATAAAATCATGAGCTTACTTGCCAAGAACCTATAGATAATTTCTACTGTTGTAGATGAAGTGATAGACTCTCCTCAAAATGAAGCCAAGAACCTATAGATAATTTCTACTGTTGTAGATATACTTTGAGCCTCTCCTGTCCGGGCTGCCAAGAACCTATAGATAATTTCTACTGTTGTAGATATTATAGAGATATTCAAACATCCAGACCAAGAACCTTTAGATAATTTCTACTGTTGTAGATGGATATCTGGCAATCCAAAAGTCATAAGCCAAGAACCTATAGATAATTTCTACTGTTGTAGATCTTTGTGCCGTTGTCGTACATAGTGCGCCAAGAACCTATAGATAATTTCTACTGTTGTAGATGAGAGGCATGGAACTATAACCAGAATGCCAAGAACCTATAGATAATTTCTATTGTTGTAGATGGGAGAGAAGCAGCTTCCGAGTCAAT |
| Prevotella ruminicola strain BPI-34 | GGTATAAACCATAGTAAAATTTCTGCTATTGCAGATCCGCAAGCAGAAGCGACTATTGCACCGCCGGTATAAACCATAGTAAAATTTCTGCTATTGCAGATTATGACGCATGGAGTGCTTATGAATTGGACGGTATAAACCATAGTAAAATTCTGCTATCGCAGATCGGATTCGACTTTAAAATCAAGCCTTACATGGTATAAACCATAGTAAAATTTCTGCTATTGTAGATATCGGGCCGTAGGCATCAGCGACTGGATCTGGTATAAACCATAGCAAAATTTCTGCTATTGCAGATTCCTGGAACTCATCCTCTTCAAAAAAT |
| Candidatus Saccharibacteria bacterium QS_5_54_17 qs_5_scaffold_44 | GTCTAATACCTACATCCAATTTCTACTTCGGTAGATCTCTGCCGGGATGGGTACATTCTCGAATGTCTAATACCTACATCCAATTTCTACTTCGGTAGATTATCATCCTTAGGCACCCTTACAGTAGTGTCTAATACCTACATCCAATTTCTACTTCGGTAGATGCGGGGCGGGCGAAGACGTTCATAGGTCTAATACCTACATCCAATTTCTACTTCGGTAGATACTTTGCGCCTTGGCGAGCGTGCGGGGTCTAATACCTACATCCAATTTCTACTTCGGTAGATTACTTCGGTGGATACCTACGTCGATGTCTAATACCTACATCCAATTTCTACTTCGGTAGATGATTGTATTTTTACCTCGCCTCCTCGTCTAATACCTACATCCAATTTCTACTTCGGTAGATTATGACACATCCGGCACTGACGGGGTGTCTAATACCTACATCCAATTTCTACTTCGGTAGATAGGTCAAAGAGGTTGCGGCTTTCATCCGGAGTCTAATACCTACATCCAATTTCTACTTCGGTAGATAGGCGGATTTGGCTTTTATTTGTCTAATACCTATATCCAATTTCTACTTCGGTAGATTTTACTCAGGACCTCGACAAATTGTAGCA |

crRNA consists of a direct repeat and a spacer. List of partial crRNA array sequences are above. Direct repeats are in blue, spacers are in purple.

**Additional file 2: Table S2.** TTTN PAM distribution.

| chr | chr_length | TTTa\|g\|c_count | average(bp) |
| --- | --- | --- | --- |
| 1 | 248956422 | 9858072 | 25.254068138272878 |
| 10 | 133797422 | 5788102 | 23.115940596762115 |
| 11 | 135086622 | 5813375 | 23.237211086503105 |
| 12 | 133275309 | 5952895 | 22.38831845681807 |
| 13 | 114364328 | 4744164 | 24.106318415636558 |
| 14 | 107043718 | 4036369 | 26.519804804763886 |
| 15 | 101991189 | 3622250 | 28.156860790944855 |
| 16 | 90338345 | 3129579 | 28.865973666106527 |
| 17 | 83257441 | 3110798 | 26.764013928258922 |
| 18 | 80373285 | 3760717 | 21.371798250174102 |
| 19 | 58617616 | 1941475 | 30.192310485584414 |
| 2 | 242193529 | 10934710 | 22.14905827406488 |
| 20 | 64444167 | 2555661 | 25.216242295046175 |
| 21 | 46709983 | 1813030 | 25.763491503174244 |
| 22 | 50818468 | 1356403 | 37.46561162132493 |
| 3 | 198295559 | 9195111 | 21.5653252037958 |
| 4 | 190214555 | 9249595 | 20.564636073255098 |
| 5 | 181538259 | 8473005 | 21.425487061556083 |
| 6 | 170805979 | 7923890 | 21.555824096498057 |
| 7 | 159345973 | 7162125 | 22.248421104071767 |
| 8 | 145138636 | 6596480 | 22.002437057339673 |
| 9 | 138394717 | 5349022 | 25.872901064904948 |
| Mitochondria | 16569 | 523 | 31.680688336520078 |
| X | 156040895 | 7247878 | 21.5291834382422 |
| Y | 54106423 | 1099594 | 49.20581869308126 |

**Additional file 2: Table S3.** List of sequences used in study.

| **Target ID** | **PAM** | **Guide sequence (5' to 3')** | **Assay** |
| --- | --- | --- | --- |
| EGFP | TTTA | CGTCGCCGTCCAG (13nt) | EGFP disruption |
| EGFP | TTTA | CGTCGCCGTCCAGCT (15nt) | EGFP disruption |
| EGFP | TTTA | CGTCGCCGTCCAGCTCG (17nt) | EGFP disruption |
| EGFP | TTTA | CGTCGCCGTCCAGCTCGAC (19nt) | EGFP disruption |
| EGFP | TTTA | CGTCGCCGTCCAGCTCGACCA (21nt) | EGFP disruption |
| EGFP | TTTA | CGTCGCCGTCCAGCTCGACCAGG (23nt) | EGFP disruption |
| EGFP | TTTA | CGTCGCCGTCCAGCTCGACCAGGAT (25nt) | EGFP disruption |
| EGFP | TTTA | CGTCGCCGTCCAGCTCGACCAGGATGG (27nt) | EGFP disruption |
| EGFP | TTTA | CGTCGCCGTCCAGCTCGACCAGGATGGGC(29nt) | EGFP disruption |
| EGFP | TTTA | CGTCGCCGTCCAGCTCGACCAGGATGGGCAC(31nt) | EGFP disruption |
| EGFP | NNNN | CGTCGCCGTCCAGCTCGACCAGG | PAM identification |
| PD1 | TTTA | GCACGAAGCTCTCCGATGTGTTG | Surveyor |
| HBB | TTTG | GGGATCTGTCCACTCCTGATGCT | Surveyor |
| IL12A | TTTA | GGATGCCACTAAAAGGGAAAGGG | Surveyor |
| DNMT1 | TTTG | GCTCAGCAGGCACCTGCCTCAGC | Surveyor |
| TRAC | TTTG | TTGCTCCAGGCCACAGCACTGTT | Surveyor |
| TRBC | TTTG | AGCCATCAGAAGCAGAGATCTCC | Surveyor |
| CTLA4 target1 | TTTC | AGCGGCACAAGGCTCAGCTGAAC | Surveyor |
| CTLA4 target2 | TTTA | TAGCAAAGCCAGAAGTTAAAGGT | Surveyor |
| CTLA4 target3 | TTTG | GAGCATGAAGATGGAGGAGGTGT | Surveyor |
| CTLA4 target4 | TTTC | AAAGCTTCAGGATCCTGAAAGGT | Surveyor |
| POLQ target1 | TTTA | GGCATGAATTATAATGCTGTTGG | Surveyor |
| AAVS target1 | TTTG | CTTACGATGGAGCCAGAGAGGAT | Surveyor |
| AAVS target2 | TTTC | ACTGATCCTGGTGCTGCAGCTTC | Surveyor |
| AAVS target3 | TTTA | CCTGTGAGATAAGGCCAGTAGCC | Surveyor |
| B2M target1 | TTTA | CTCACGTCATCCAGCAGAGAATG | Surveyor |
| B2M target2 | TTTC | TATCTCTTGTACTACACTGAATT | Surveyor |
| B2M target3 | TTTG | TCACAGCCCAAGATAGTTAAGTG | Surveyor |
| B2M target4 | TTTG | TAAGCTGCTGAAAGTTGTGTATG | Surveyor |
| CFTR target1 | TTTC | GTATAGAGTTGATTGGATTGAGA | Surveyor |
| CFTR target2 | TTTA | GAGAGAAGGCTGTCCTTAGTACC | Surveyor |
| CFTR target3 | TTTC | TCATTAGAAGGAGATGCTCCTGT | Surveyor |
| EGFR target1 | TTTA | TAGAGAGGTAGACTGAGGCTTCC | Surveyor |
| EGFR target2 | TTTG | GATCCAAGCCATATGACGGAATC | Surveyor |
| EGFR target3 | TTTA | CCACTTATCAGTCACTTACTACT | Surveyor |
| GRIN2B target1 | TTTG | TCTCTGCCTGTAGCTGCCAATGA | Surveyor |
| GRIN2B target2 | TTTG | TGTATGCATACTCGCATGGCTAC | Surveyor |
| GRIN2B target3 | TTTA | TTGCCTTGTTCAAGGATTTCTGA | Surveyor |
| GRIN2B target4 | TTTG | CAGGGAGTCGACGAGTTGAAGAT | Surveyor |
| GDF15 target1 | TTTG | GCACTCTTCATTTCTCGGGATTA | Surveyor |
| GDF15 target2 | TTTA | GGAGGCCAAGGTGGGAGCATCGC | Surveyor |
| GDF15 target3 | TTTA | GGCCAGAACATTGCAGCCTGGGC | Surveyor |
| GDF15 target4 | TTTA | GCAGTGCCTTGACAAGCGATTTC | Surveyor |
| GDF15 target5 | TTTA | GGGCGGGCTAAGAGTGTGCCCCT | Surveyor |
| CXCR4 target1 | TTTG | CAGATATACACTTCAGATAACTA | Surveyor |
| CXCR4 target2 | TTTG | TCATCACGCTTCCCTTCTGGGCA | Surveyor |
| Angptl3 target1 | TTTA | CTGAGAACCTCTTATGGACCAGG | Surveyor |
| CCR5 target1 | TTTG | TTTGGCCTGAATAATTGCAGTAGCTCT | Surveyor |
| MMP9 | TTTG | AGCCCAGCCTAGGCAACATAGTA | Surveyor |
| RUNX1 | TTTC | AGCCTCACCCCTCTAGCCCTACA | Surveyor |
| VEGFA 1 | TTTG | CTAGGAATATTGAAGGGGGCAGG | Surveyor |
| VEGFA 2 | TTTG | GGAGGTCAGAAATAGGGGGTCCA | Surveyor |
| VEGFA 3 | TTTG | GGACTGGAGTTGCTTCATGTACA | Surveyor |
| TCTV1 | TCTG | GCAAAACATGATCGAAAGCAGAA | Deep sequencing |
| TCTV2 | TCTG | AAGGTCTATTTTCCTCCTCTGAC | Deep sequencing |
| TCTV3 | TCTA | GGGTAAGATTCTTACTCTTACCC | Deep sequencing |
| TCTV4 | TCTA | TGAGACTGGAAGTGATTTAAGAG | Deep sequencing |
| TCTV5 | TCTA | TCTGGCCTTATTAAACTCCTGAC | Deep sequencing |
| TCTV6 | TCTA | GATTAGACATCTCCCACTCAGTG | Deep sequencing |
| TCTV7 | TCTA | AATGGAAGGGATTGGGGAATATT | Deep sequencing |
| TCTV8 | TCTA | CAATTATCTCAATTGTAAATGAT | Deep sequencing |
| TCTV9 | TCTA | CCCTGCTCCCCGGACTCCGCCTG | Deep sequencing |
| TCTV10 | TCTA | TGAAAGCTAGAGAGATGGCTGAA | Deep sequencing |
| TCTV11 | TCTA | GATTGTCACCTCCTTACACCCAG | Deep sequencing |
| TTCV1 | TTCA | TTAGATTCCCCAATCCACCTCTT | Deep sequencing |
| TTCV2 | TTCA | GATGCAGATCCTCAGTTTTCAGC | Deep sequencing |
| TTCV3 | TTCA | GCATAAGCTCACCACAAAGGAGA | Deep sequencing |
| TTCV4 | TTCA | TGGCACACAGTACACACATTTGT | Deep sequencing |
| TTCV5 | TTCA | CACTTGGTTGACACAACTGTCCT | Deep sequencing |
| TTCV6 | TTCA | CATATGCTATCTTGTTTGCCCTA | Deep sequencing |
| TTCV7 | TTCA | TCCTAGGTCTGCCAGCCTCAGAG | Deep sequencing |
| TTCV8 | TTCA | GAGTCAATGGCTATCTAGCTCTT | Deep sequencing |
| TTCV9 | TTCA | GCCTACTAGTCCAGGCCCAATGC | Deep sequencing |
| TTCV10 | TTCA | GGCACGCCCTCGGGACGCCACCC | Deep sequencing |
| TTCV11 | TTCA | GGCTTCAGTGAGTTATGATCATG | Deep sequencing |
| TTCV12 | TTCA | GGGGCCTTCTGACCACTCTGAAC | Deep sequencing |
| TCCV1 | TTCA | TTTGATCATAATGGAAAGTATGT | Deep sequencing |
| TCCV2 | TCCA | CTCTACAACAATGCCAGGGAAGG | Deep sequencing |
| TCCV3 | TCCA | GAGCACAAGTTGTATGAGATTCA | Deep sequencing |
| TCCV4 | TCCA | GGTGGCTGTGTCTGCAGGGACAG | Deep sequencing |
| TCCV5 | TCCA | CCTCCTTAAATAGGCCTGGTTCT | Deep sequencing |
| TCCV6 | TCCA | TGTGTCCTTAACCTCATGGCCTT | Deep sequencing |
| TCCV7 | TCCA | CATCTTCATCCTCTTCTTCCTCT | Deep sequencing |
| TCCV8 | TCCA | GGACATGTGTCCAAGCCCTGGCA | Deep sequencing |
| TCCV9 | TCCA | GTCAGCGCTGCCACTGCTGAGCT | Deep sequencing |
| TCCV10 | TCCA | CATGGCTAAGGAAGCCTCATGAT | Deep sequencing |
| CTTV1 | CTTA | CATTCTTTTGTAAGCTGCTGAAA | Deep sequencing |
| CTTV2 | CTTG | AGAATTAGATGCCATTATCTGGA | Deep sequencing |
| CTTV3 | CTTA | ATTTCAGGAGGTTTACTTTTAGG | Deep sequencing |
| CTTV4 | CTTA | TTGGTGGCTAATTGGTCATGGCC | Deep sequencing |
| CTTV5 | CTTA | TCTGAAAAGCATCCCCCATCCTC | Deep sequencing |
| CTTV6 | CTTA | AATGGTACAAGATGAACTTCTCC | Deep sequencing |
| CTTV7 | CTTA | AGTAAGAATTGTTGTGCATTTTG | Deep sequencing |
| CTTV8 | CTTA | ACCTCATGGTTTTACCAACGTCA | Deep sequencing |
| CTTV9 | CTTA | AAAAGAAACGTTTGGTGGTTCG | Deep sequencing |
| CTTV10 | CTTA | CCTAGTCCCAGCTGCCGTCACCC | Deep sequencing |
| CTTV11 | CTTA | CTTATAAGCAGATGGTTGGCCGT | Deep sequencing |
| CTCV1 | CTCA | CTGATTGTGGACTAGTCCCATAG | Deep sequencing |
| CTCV2 | CTCA | CATGACACCTATCCCTGTTATCC | Deep sequencing |
| CTCV3 | CTCA | GGCTGCTGACCCTGGGCCTGGGT | Deep sequencing |
| CTCV4 | CTCA | CGTCACAGTATGTCTCTGGCGTT | Deep sequencing |
| CTCV5 | CTCA | CGTCACAGTATGTCTCTGGCGTT | Deep sequencing |
| CTCV6 | CTCA | ACAGACATGATTAACCTCATTGT | Deep sequencing |
| CTCV7 | CTCA | TCACTAATATCAGCAGTTTCATT | Deep sequencing |
| CTCV8 | CTCA | TCACTAATATCAGCAGTTTCATT | Deep sequencing |
| CTCV9 | CTCA | GTGCTGACCTGTCTCCAGTGATA | Deep sequencing |
| CCTV1 | CCTA | CAGGGTCATGTTCCCTTCTCCTG | Deep sequencing |
| CCTV2 | CCTG | CCACTGCTGTGTGTTCCTCTTGA | Deep sequencing |
| CCTV3 | CCTA | TCTTCCATTCAAGGCAAATGATT | Deep sequencing |
| CCTV4 | CCTA | CTCCAAGTTCAAAAACTCTCTGG | Deep sequencing |
| CCTV5 | CCTA | GTAGATAGCATGTGCAGAGCGGC | Deep sequencing |
| CCTV6 | CCTA | GTATTCTAACCAGTTAACCAGCC | Deep sequencing |
| CCTV7 | CCTA | GTTGTGTTAGTTTACCTGGGTGT | Deep sequencing |
| CCCV1 | CCCA | GGGTACATTGATGCTGAAACCCC | Deep sequencing |
| CCCV2 | CCCA | CTATTACCCCTTTATTTTCAAAC | Deep sequencing |
| CCCV3 | CCCA | AAGAAGGAAAAGGCAGGGAGCGA | Deep sequencing |
| CCCV4 | CCCA | TCCTCAGGGACCACATCTGTAAG | Deep sequencing |
| CCCV5 | CCCA | GCCTGGAGCTGTCTACTACATAC | Deep sequencing |
| CCCV6 | CCCA | GCACCTTAGAAGGCCCATGTAGG | Deep sequencing |
| CCCV7 | CCCA | GGAGAATTTCTTATTAGAGGGAA | Deep sequencing |
| CCCV8 | CCCA | GCCAGCCTGCACCTGGGGATGCC | Deep sequencing |
| CCCV9 | CCCA | CAGCCCACCCCAGCCCCTCACCC | Deep sequencing |
| CCCV10 | CCCA | TTCAGGAAAAGGGCAGTTCATTT | Deep sequencing |
| CCCV11 | CCCA | AGAATGTGGCTGGGAGGAATCTC | Deep sequencing |
| CCR5 target2 | TTTA | TGCACAGGGTGGAACAAGATGG | GUIDE seq |
| POLQ target2 | TTTA | AACAGAAACATGCATAAAACAAGG | GUIDE seq |
| HEK site1 | TTTC | TGATGGTCCATACCTGTTACACT | GUIDE seq |
| DNMT1 | TTTG | GCTCAGCAGGCACCTGCCTCAGC | GUIDE seq |
| IL12A | TTTA | GGATGCCACTAAAAGGGAAAGGG | GUIDE seq |
| **Primer Name** | | **Sequence** | **Assay** |
| PD1-F | | TCTCCATCTCTCAGACTCCCCAGACAG | Surveyor |
| PD1-R | | TAGAGGGGCTGGGGTGCTTCCAGAG | Surveyor |
| HBB-F | | TAAGCCAGTGCCAGAAGAGC | Surveyor |
| HBB-R | | CGTCCCATAGACTCACCCTGA | Surveyor |
| IL12A-F | | ATGCTTGCTGTATACACAAGGC | Surveyor |
| IL12A-R | | CTACCGTTTACCTCCTGGAAC | Surveyor |
| DNMT1-F | | GGGTACATGTGGGGGCAGTT | Surveyor |
| DNMT1-R | | AGAATGCACAAAGTACTGCACAAT | Surveyor |
| TRAC-F | | TCATGTCCTAACCCTGATCCTCTT | Surveyor |
| TRAC-R | | TTGGACTTTTCCCAGCTGACAGA | Surveyor |
| TRBC-F | | AATGAGGAGACATCACCTGGAATG | Surveyor |
| TRBC-R | | GATGCACACCACTCAGATGCTG | Surveyor |
| CTLA4-F | | CCCTTGTACTCCAGGAAATTCTCCA | Surveyor |
| CTLA4-R | | GGCATTCTTCCCACAATTTCCCTAC | Surveyor |
| POLQ-F | | GCCAAAGCTCTGCGTTCTTGTTTC | Surveyor |
| POLQ-R | | CTGATGGCTGAAATGCAGATGTG | Surveyor |
| VEGFA-F | | CTCAGCTCCACAAACTTGGTGCC | Surveyor |
| VEGFA-R | | AGCCCGCCGCAATGAAGG | Surveyor |
| GDF15-F | | CCTGATTTGCACCCACAACG | Surveyor |
| GDF15-R | | AGGCAGCCTGAGATTCCAAC | Surveyor |
| RUNX1-F | | CCAGCACAACTTACTCGCACTTGAC | Surveyor |
| RUNX1-R | | CATCACCAACCCACAGCCAAGG | Surveyor |
| MMP9-F | | TGATGGCCAGAAATGGGCAA | Surveyor |
| MMP9-R | | GACAGGACATGTTCACCGCT | Surveyor |
| GRIN2B-F | | GGCAAACATAAGGTGAAGGCAG | Surveyor |
| GRIN2B-R | | AATCGAGGATCTGGGCGATG | Surveyor |
| CFTR-F | | GCTGTGTCTGTAAACTGATGGCTAACA | Surveyor |
| CFTR-R | | TTGCATTCTACTCAATTGCATTCTGTGGG | Surveyor |
| Angptl3-F | | CAGGTAAAACCTGTCTAAGGAGA | Surveyor |
| Angptl3-F | | TGTCTGAGTTTCCTGGTCCTT | Surveyor |
| EGFR-F | | AAATGTGCAGTTTACAGCCCT | Surveyor |
| EGFR-R | | ATTTGCTTGCAAACATGGGCA | Surveyor |
| TCTV1-F | | TCCAAGATGGTTACCAAGACTG | Deep sequencing |
| TCTV1-R | | TAACCAGCAAATACCAGTAATG | Deep sequencing |
| TCTV2-F | | GAAAATTTCCAAAGTAATACATGCCATG | Deep sequencing |
| TCTV2-R | | CTGTAATCTTTTCTAAGAAGAGGAC | Deep sequencing |
| TCTV3-F | | ACCCAGACACTAGCAACAGT | Deep sequencing |
| TCTV3-R | | AGTCCAGGTCTTTCTTCCCT | Deep sequencing |
| TCTV4-F | | CGGCAGCAAATAAGGCAAAGAG | Deep sequencing |
| TCTV4-R | | CTCTTCCCAGGATTTGCTTAAGTC | Deep sequencing |
| TCTV5-F | | GCTCATTGCCTTCATCACCTTAT | Deep sequencing |
| TCTV5-R | | GATAGGGGAGTAAGGCCCCGTGG | Deep sequencing |
| TCTV6-F | | GCTCTTGAATTTACGGAAACGGC | Deep sequencing |
| TCTV6-R | | TATCTTTTCCTGGAACATGGGAC | Deep sequencing |
| TCTV7-F | | AGTTTAGGAGATAAGACCTATCTG | Deep sequencing |
| TCTV7-R | | GAGTTCTGTGGGACAGACTATGATG | Deep sequencing |
| TCTV8-F | | CGGGGACTCAGGATGGGATCGAGG | Deep sequencing |
| TCTV8-R | | CAGGCGTGAGCCACCACACCTGGC | Deep sequencing |
| TCTV9-F | | GGTTCCCTGGGAAGGAAGGCTGA | Deep sequencing |
| TCTV9-R | | AATCCCCAACACACGATTTCCAA | Deep sequencing |
| TCTV10-F | | GGTGGGAGAGATGGTCAAGGTCTC | Deep sequencing |
| TCTV10-R | | GGGAAGGGGTAGGGGAGTGGAGG | Deep sequencing |
| TCTV11-F | | CAGAGGAGGGAGGAGGCATCCCC | Deep sequencing |
| TCTV11-R | | GTTGAGCACCAACCTGTCTAAGGC | Deep sequencing |
| TTCV1-F | | TCCAGGCAGGATGAATCTGT | Deep sequencing |
| TTCV1-R | | GGCAGCTACTCCTCCTTGTC | Deep sequencing |
| TTCV2-F | | TTAATTCAAGGTTTTAAGGTTC | Deep sequencing |
| TTCV2-R | | GGATATACTCATCATTTTAAGCC | Deep sequencing |
| TTCV3-F | | CCCTGCAGTTCCCTAACTGAGGGGG | Deep sequencing |
| TTCV3-R | | CCACCACGGCCTCCTCGTCGCTCC | Deep sequencing |
| TTCV4-F | | CCCAATTTACTGTATCCCAGGAATTTG | Deep sequencing |
| TTCV4-R | | TAAGAATTTTGCTATGAGAGACTAG | Deep sequencing |
| TTCV5-F | | CACCTGGCCTTACTCACCTCTTA | Deep sequencing |
| TTCV5-R | | GGCAGGGGAGTAAACCTAGGGCT | Deep sequencing |
| TTCV6-F | | CCATTTATGGAGTCTCTACCATA | Deep sequencing |
| TTCV6-R | | GCGATGCCCGTCTCCAATCCGTC | Deep sequencing |
| TTCV7-F | | GCCTAAGCCCCAGCTGCCCTGTG | Deep sequencing |
| TTCV7-R | | GAATGATAGGTTACATTGAAAGAAGG | Deep sequencing |
| TTCV8-F | | ATTCTCTTCCTCTGAGTCCAAGAG | Deep sequencing |
| TTCV8-R | | GCTGTAAGAGTAAAACTCTCTTTCC | Deep sequencing |
| TTCV9-F | | GCGGGGGAACCAGAGGGCAGAGG | Deep sequencing |
| TTCV9-R | | TGTTACCAGTGGGGAAACCGAGG | Deep sequencing |
| TTCV10-F | | GGGCCAGCTGCTATGCTCAGTGG | Deep sequencing |
| TTCV10-R | | GCCCACTCCAAGTCCCCACCTAGGC | Deep sequencing |
| TTCV11-F | | GCCATCCCCACTAGATGGCAGAG | Deep sequencing |
| TTCV11-R | | GGGGGCTGCTAGTGACACCCTCTC | Deep sequencing |
| TTCV12-F | | GGTCTGGAGTGTGATATTGGGGT | Deep sequencing |
| TTCV12-R | | TCCACATTGCTACTCGCTGGAGC | Deep sequencing |
| TCCV1-F | | GTACTGTTTCTGAAACATTAGGC | Deep sequencing |
| TCCV1-R | | AACCTAAATGCATACAAGAGCTG | Deep sequencing |
| TCCV2-F | | GAGGCAGCGGTGGTGTCAGG | Deep sequencing |
| TCCV2-R | | GGTACAGAATCTTGCTTTATC | Deep sequencing |
| TCCV3-F | | CACAAGGCGGCTTGGCTGCA | Deep sequencing |
| TCCV3-R | | TGCCTCATGGTCCTCAACGT | Deep sequencing |
| TCCV4-F | | TAGGCGGCAGCTGTTCGATTGG | Deep sequencing |
| TCCV4-R | | GGCAGACATCCTGTGCTAGC | Deep sequencing |
| TCCV5-F | | ATACGTTACTATGCTCAAGGAAG | Deep sequencing |
| TCCV5-R | | GTTGAAAGTTCAAAGCTGAGTGTTC | Deep sequencing |
| TCCV6-F | | CCTATATGTACATGCCCTCTTCC | Deep sequencing |
| TCCV6-R | | AATAGAGCTAGGAGGCCAACTCT | Deep sequencing |
| TCCV7-F | | AGCTGTCCTGAGCGAATCCCATTTC | Deep sequencing |
| TCCV7-R | | AAGATGGGATCGCGGAACAGCAG | Deep sequencing |
| TCCV8-F | | GGGACTGAGAGGAGGAGGGAAGG | Deep sequencing |
| TCCV8-R | | TGGGCCCAGGAGGTGGGCTCTGA | Deep sequencing |
| TCCV9-F | | CCCGGGGCCACCACTCCCACCTC | Deep sequencing |
| TCCV9-R | | GCAGCCTTGCCAGGAGGCGTCCT | Deep sequencing |
| TCCV10-F | | GGGGTTCGGGGT TCAGGGGCAGCC | Deep sequencing |
| TCCV10-R | | GTAAGTCTCACGAGATCTGATGG | Deep sequencing |
| CTTV1-F | | GATGAGTATGCCTGCCGTGT | Deep sequencing |
| CTTV1-R | | CAGATGGGATGGGACTCATTC | Deep sequencing |
| CTTV2-F | | CACTTTCTCACCTGCTCTTCCTG | Deep sequencing |
| CTTV2-R | | AGCAAAGATTTTGAAGCCCAAG | Deep sequencing |
| CTTV3-F | | GGTGAGCAAAGCCATTTCAC | Deep sequencing |
| CTTV3-R | | GATGGTTAGCACTCCAGAGC | Deep sequencing |
| CTTV4-F | | CCCAGGCATGGATGAGGTAGCCC | Deep sequencing |
| CTTV4-R | | CGCACTGTCCCCTCTCTAACGCAG | Deep sequencing |
| CTTV5-F | | GGCAGAGATTGAGCCAGAAGGAC | Deep sequencing |
| CTTV5-R | | CGGGGGGAGGGGTGAAATGTGGT | Deep sequencing |
| CTTV6-F | | TGCCTCCGGCCTGAAAAGTCAGTA | Deep sequencing |
| CTTV6-R | | GCAAATGTACAGGCATCACTAATTC | Deep sequencing |
| CTTV7-F | | GCTGTGTCTTTAGGACTGGAAGG | Deep sequencing |
| CTTV7-R | | ATAGCTTAATTGACTCTTCTGTC | Deep sequencing |
| CTTV8-F | | GGAGGCCTGAACTTACTGGCCTT | Deep sequencing |
| CTTV8-R | | TTAATGTTGGGTAGCTAAGATCA | Deep sequencing |
| CTTV9-F | | CGAGGTGCCTGAGGGGAGGAGAAAG | Deep sequencing |
| CTTV9-R | | AGGAGGCCCTTGGGCACTGTCTG | Deep sequencing |
| CTTV10-F | | CCTGGCTGGGGGGTTTTGTGGCC | Deep sequencing |
| CTTV10-R | | TGTGGGCCGAACCGTGTCCCCAC | Deep sequencing |
| CTTV11-F | | GGGGTGAATTTACCTGCACGTCG | Deep sequencing |
| CTTV11-R | | GATGACACTAGTGCATTGGATAA | Deep sequencing |
| CTCV1-F | | CAAACCATAATTCTGGTGTCATTC | Deep sequencing |
| CTCV1-R | | CAAATGGGTGTGTGTGCATTCC | Deep sequencing |
| CTCV2-F | | GGACCACTTGGATGGTCTTTATTC | Deep sequencing |
| CTCV2-R | | AAGAAATCCTGACAGGAGGCCTG | Deep sequencing |
| CTCV3-F | | CTCCTCTGACCAGGAGCACG | Deep sequencing |
| CTCV3-R | | ATGGCCCGCCTCCTAGTCTG | Deep sequencing |
| CTCV4-F | | AGAATTCAGCATAGCGCCTGGCA | Deep sequencing |
| CTCV4-R | | CCGGAACCACGTGCGAGATGATG | Deep sequencing |
| CTCV5-F | | CCAGGCGGAGCTGCTGCTGGAGC | Deep sequencing |
| CTCV5-R | | TCCCCAGAAGCCAGTGGACTAGCAC | Deep sequencing |
| CTCV6-F | | CCGGGTATTAGCCTCCTGTTATT | Deep sequencing |
| CTCV6-R | | GTAAGGCCAAGAGTTTCGTAGGG | Deep sequencing |
| CTCV7-F | | CCCTTCAGAGCCGGGCCGAGTGTC | Deep sequencing |
| CTCV7-R | | GGGTGGGGTGGTTCAGGGGGTCC | Deep sequencing |
| CTCV8-F | | GCGGGAGCACCCCCACCGCCACC | Deep sequencing |
| CTCV8-R | | GCCTCCCTTGGCTGGTAGTGTCT | Deep sequencing |
| CTCV9-F | | GGTATCTGGGCTTCTAGGGGCAAC | Deep sequencing |
| CTCV9-R | | CTCTTTGTCCTCTCCCTATCTGCC | Deep sequencing |
| CCTV1-F | | GTAAGCAGCATCATGGAGGTAAG | Deep sequencing |
| CCTV1-R | | CCTTTGCCCTCTCTGTAGAGGGTCAG | Deep sequencing |
| CCTV2-F | | AGCCCTTTCTGACTTCCACAGGCTG | Deep sequencing |
| CCTV2-R | | GCCCCAGCTCAAGCGCCAACAAGC | Deep sequencing |
| CCTV3-F | | AGCTTGTTCCCTTGCAGACAAGATTG | Deep sequencing |
| CCTV3-R | | CTCAACACATATTGGGTCCCTAGC | Deep sequencing |
| CCTV4-F | | CAGAAGGGAGACGTGGAGAT | Deep sequencing |
| CCTV4-R | | GCAGAGGCCTCAGTGCAGG | Deep sequencing |
| CCTV5-F | | TACTCCCTGGTGAGGCTTGG | Deep sequencing |
| CCTV5-R | | GGGTGGCAGTGTGCCTGGAT | Deep sequencing |
| CCTV6-F | | CTAGGTTTTCTGTCCCTCCCTCAG | Deep sequencing |
| CCTV6-R | | TGCGTCAATGCTTTAAAGGGACATAC | Deep sequencing |
| CCTV7-F | | AATCACAACCTTGGGGCCACAGTTG | Deep sequencing |
| CCTV7-R | | ATATCTTGATTGCTGTAAAGGTCTATC | Deep sequencing |
| CCCV1-F | | AGGAGCTCCAGAAGCAAGAG | Deep sequencing |
| CCCV1-R | | ACAGTGTGACTGGGCAGATC | Deep sequencing |
| CCCV2-F | | TGTTCCACAAGTTAAATAAATC | Deep sequencing |
| CCCV2-R | | TAGACAGCTGTGTCACCCCAAC | Deep sequencing |
| CCCV3-F | | CTGTCACATCAGCTCCACTTTC | Deep sequencing |
| CCCV3-R | | ATGTAATCATACATTTGTTTAAG | Deep sequencing |
| CCCV4-F | | CCGACCTGCCGGAGGTGCGGAATGG | Deep sequencing |
| CCCV4-R | | GGACTCGGGAGGAGGAGCCTGGG | Deep sequencing |
| CCCV5-F | | CTTCCGCCCAGTCTCCAGTCACA | Deep sequencing |
| CCCV5-R | | AGAATTGCTTGAACCCGGGAGGT | Deep sequencing |
| CCCV6-F | | GGTACTCTGTGACCTTGGACAAG | Deep sequencing |
| CCCV6-R | | GATCCTCCCGCCTCAGCGTCTGG | Deep sequencing |
| CCCV7-F | | GGCTCACTATTGTTCATTGTTTC | Deep sequencing |
| CCCV7-R | | TGTCCGGCCTAGATTTCTGGAAG | Deep sequencing |
| CCCV8-F | | CCCGGATCGAGGAGAGAGGGAGC | Deep sequencing |
| CCCV8-R | | GGGTGCCCCTCATCTTGTCCTCC | Deep sequencing |
| CCCV9-F | | CCAGGGCTCTGTCCTGCACCTGG | Deep sequencing |
| CCCV9-R | | CCCAGCAGGCCGCCCACGACACC | Deep sequencing |
| CCCV10-F | | GGATGATGGTGCTTGGTGCCAAA | Deep sequencing |
| CCCV10-R | | GATAAACAAGCCACTTTGGGCTG | Deep sequencing |
| CCCV11-F | | CGCTATGTAGTGAGCAGAGGGGA | Deep sequencing |
| CCCV11-R | | CCCAAGGTTATATGATTACTAAG | Deep sequencing |
| DNMT1-F | | GTCAAGTGCTTAGAGCAGGCG | Deep sequencing |
| DNMT1-R | | CATCAGGAAACATTAACGTAC | Deep sequencing |
| DNMT1-OT1F | | CTGATGCTACTCTGCTACCATT | Deep sequencing |
| DNMT1-OT1R | | GAGTCTTGCTGAGAACCTCAAG | Deep sequencing |
| DNMT1-OT2F | | GTCAGTGCCACCAAGGCGGGTA | Deep sequencing |
| DNMT1-OT2R | | TAGGCTGAGACCTGAGTCTGGG | Deep sequencing |
| DNMT1-OT3F | | GCACTCCCAGCCTGGGTGACAG | Deep sequencing |
| DNMT1-OT3R | | ACTTGGAGGGAGAGCAGGTCTA | Deep sequencing |
| DNMT1-OT4F | | TGAGAGCATCTCTTAGTATTGT | Deep sequencing |
| DNMT1-OT4R | | GTCTTCATTTCTGTAAGTGATC | Deep sequencing |
| DNMT1-OT5F | | GTTGTATGTTTATAATAGAATT | Deep sequencing |
| DNMT1-OT5R | | CCTGGACAACACAGAAAGCCTG | Deep sequencing |
| DNMT1-OT6F | | CAGCACTTTGGGAAGCCGAGGT | Deep sequencing |
| DNMT1-OT6R | | GACAGAGTATTGCTCTGTCACC | Deep sequencing |
| DNMT1-OT7F | | GGAAGGGGTGCAATAGGCATGG | Deep sequencing |
| DNMT1-OT7R | | GGAAATGTAAGGTCAGGGAACA | Deep sequencing |
| DNMT1-OT8F | | TGTCCACAGTGATGTGAGATGT | Deep sequencing |
| DNMT1-OT8R | | CCCCAGTGCAATGTGCCCCTCT | Deep sequencing |
| HBB-F | | TGGGTTTCTGATAGGCACTGAC | Deep sequencing |
| HBB-R | | CTCACTCAGTGTGGCAAAGG | Deep sequencing |
| HBB-OT1F | | TACTGGCTTTAGGGTGGAGTCC | Deep sequencing |
| HBB- OT1R | | CAGAGAAGGCACCACTAATCC | Deep sequencing |
| HBB-OT2F | | GCTTCAACATTGCAAAGGAG | Deep sequencing |
| HBB-OT2R | | GCCTTTAAACGATTGTGGG | Deep sequencing |
| HBB-OT3F | | AAGTTTAGCCTCGACCACTATGTAAC | Deep sequencing |
| HBB-OT3R | | TGTAGAACAGAGGCAAACCACTGTTGC | Deep sequencing |
| HBB-OT4F | | GTTAGTCTGATGGGGATTCC | Deep sequencing |
| HBB-OT4R | | ACATAGGATACCATAAAGTGAC | Deep sequencing |
| HBB-OT5F | | CCCTTCCTTGGGTTCAGAG | Deep sequencing |
| HBB-OT5R | | CTATTCGCATATAACATCATTGC | Deep sequencing |
| HBB-OT6F | | AACTGCCTTTGCAAAACCTCA | Deep sequencing |
| HBB-OT6R | | ACAGGATACATAGGCAGACGA | Deep sequencing |
| HBB-OT7F | | GGCTTTGGATTGCCTTGCAAG | Deep sequencing |
| HBB-OT7R | | CAACTGATACTAGAATTCAATAACC | Deep sequencing |
| HBB-OT8F | | GCACTGCACTGTCTTCTCCTG | Deep sequencing |
| HBB-OT8R | | ATAAACATACAACATATAGAAATACAC | Deep sequencing |
| IL12A-F | | TTTCCCTGAAAAGGTGTTGC | Deep sequencing |
| IL12A-F | | TCCTTCCATCTGGGTTTCTG | Deep sequencing |
| IL12A-OT1-F | | GTGGAACAGAGGAGGAAAAG | Deep sequencing |
| IL12A-OT1-R | | GGGAAGCTAGCGAGAAGGTTAAGG | Deep sequencing |
| IL12A-OT2-F | | GGTCTGATCCAATGCTCCCTC | Deep sequencing |
| IL12A-OT2-F | | GTGAGTAATCACAGTACCTG | Deep sequencing |
| IL12A-OT3-F | | TGCTGGCTGCGATTGTAACTGTC | Deep sequencing |
| IL12A-OT3-R | | TTGTCACCCTGTCCTCACTG | Deep sequencing |
| IL12A-OT4-F | | TGCCTGGCAAATAATCAGTGG | Deep sequencing |
| IL12A-OT4-R | | AGACCAGGGCAGAGGAATCA | Deep sequencing |
| IL12A-OT5-F | | ACGATGTGGACTAGATTAAG | Deep sequencing |
| IL12A-OT5-R | | CTACTTTCCTTTCTTCTGTG | Deep sequencing |
| IL12A-OT6-F | | ACTGAAAACAACTTTTCTTAATTG | Deep sequencing |
| IL12A-OT6-R | | CACATTTATACATCTGTAAATTC | Deep sequencing |
| IL12A-OT7-F | | TCGCTTTTGAAAGTATCCAAAGC | Deep sequencing |
| IL12A-OT7-F | | CAATGCGGCCTAATTTACAC | Deep sequencing |
| IL12A-OT8-F | | CTGAGGTCCAGTTAAAATTG | Deep sequencing |
| IL12A-OT8-R | | CTGCCCCTCCTGGCCCTGCAG | Deep sequencing |
| POLQ-F | | GCAAAGTACTGAAATGCTATC | Deep sequencing |
| POLQ-R | | TACTGAACCCTTGCTGCCAAG | Deep sequencing |
| POLQ-OT1-F | | GTGTAGTGCTGAAGTGCGGTC | Deep sequencing |
| POLQ-OT1-R | | TCCAACCAGCAGTACATGAAG | Deep sequencing |
| POLQ-OT2-F | | CTCTATTTAGTGCCAGTATTTTG | Deep sequencing |
| POLQ-OT2-R | | GTCGTCTGACCAGTACATAAC | Deep sequencing |
| POLQ-OT3-F | | TGAGACGGAGTCTCGCTCTG | Deep sequencing |
| POLQ-OT3-R | | CATAATACTGAAGTGCTGTC | Deep sequencing |
| POLQ-OT4-F | | GGTTCCTACAAGCCCCTGGACAC | Deep sequencing |
| POLQ-OT4-R | | GCTTTCTGCTGGTTATTTCAC | Deep sequencing |
| POLQ-OT5-F | | CAAGCACAGTACTGAAGTGCTG | Deep sequencing |
| POLQ-OT5-R | | TCTAATCACAACACTTTCATC | Deep sequencing |
| POLQ-OT6-F | | ACATAGTGCTAGAGGGCTGTC | Deep sequencing |
| POLQ-OT6-R | | GAAATATAGGCTTGGGATTCCATG | Deep sequencing |
| POLQ-OT7-F | | GCACTTTGAAGAACAGCCTC | Deep sequencing |
| POLQ-OT7-R | | GCCTCCCAAGTGTAGTGCTG | Deep sequencing |
| POLQ-OT8-F | | GGAGGTTACATAGCCAAGCC | Deep sequencing |
| POLQ-OT8-R | | GCATATATAATGTACATACAAC | Deep sequencing |
| POLQ-OT9-F | | GCTTTCAAATATCCCCAAGTCG | Deep sequencing |
| POLQ-OT9-R | | CGTAAATACAGGACTACGTTC | Deep sequencing |
| POLQ-OT10-F | | TAAGATGGCCCTCAAGATAGTG | Deep sequencing |
| POLQ-OT10-R | | TGATCCTAGGGGAAATCTAGGG | Deep sequencing |
| B2M-F | | TTGACACCAAGTTAGCCCCA | Deep sequencing |
| B2M-R | | AGACCAGTCCTTGCTGAAAGA | Deep sequencing |
| B2M-OT1-F | | CCAGCCATGGGCTCTGTTATC | Deep sequencing |
| B2M-OT1-R | | ATCCTCTCTCTGCTGCTGACAC | Deep sequencing |
| B2M-OT2-F | | CTGATATATGTATCAAGTCAGG | Deep sequencing |
| B2M-OT2-R | | CAGTGAGCCGAGATCGTAC | Deep sequencing |
| B2M-OT3-F | | CTCTGGAGTTTCTCGCGCAT | Deep sequencing |
| B2M-OT3-R | | ATACCCAAATGCAGCAAGCC | Deep sequencing |
| B2M-OT4-F | | AGTCCAAACTCTTCCCACCTC | Deep sequencing |
| B2M-OT4-R | | CTGTCTGAGCTGTGTTCTCCC | Deep sequencing |
| B2M-OT5-F | | GTCTCAGATGGTCTATGTAAGTCG | Deep sequencing |
| B2M-OT5-R | | GCGGAGGCCACAGTGAGC | Deep sequencing |
| B2M-OT6-F | | GTGAGCTGAGGTCACGCCAC | Deep sequencing |
| B2M-OT6-R | | GCACTGTGTGTGACATGGGAAG | Deep sequencing |
| B2M-OT7-F | | ACGGTAGGCTATAGAAACTCTGT | Deep sequencing |
| B2M-OT7-R | | TCATCACCTTGCAGATGCTACT | Deep sequencing |
| **Gene** | | **Target sequence** | **Assay** |
| DNMT1 | | TTTGGCTCAGCAGGCACCTGCCTCAGC | Deep sequencing |
| DNMT1 OT1 | | CTTAGCTCAGCAGGCACCTGCCCATGG | Deep sequencing |
| DNMT1 OT2 | | TTTAGCTatGCAGGCAgCaGCCTCAGC | Deep sequencing |
| DNMT1 OT3 | | TTTAGCTATGCAGGCAGCAGCCTCAGC | Deep sequencing |
| DNMT1 OT4 | | TTTGcCTTCAGCAaGCACCTcaCTCAGC | Deep sequencing |
| DNMT1 OT5 | | TTTGGCTCAcgAGAGcCtCCTGCCTCAGC | Deep sequencing |
| DNMT1 OT6 | | TTTGGCTCAagAGaTCCtCCTGCCTCAGC | Deep sequencing |
| DNMT1 OT7 | | TTTGGCTCAGCAtGCACCgaCCTTCAGt | Deep sequencing |
| DNMT1 OT8 | | TTTCaCTCtGCAGAGCACCTGCCTCAGg | Deep sequencing |
| HBB | | TTTGGGGATCTGTCCACTCCTGATGCT | Deep sequencing |
| HBB OT1 | | GGGATCTGTCCACTttTaATtCTTGG | Deep sequencing |
| HBB OT2 | | TTTGGGGATCTaaCtACTtCTtATaCT | Deep sequencing |
| HBB OT3 | | TTTGGGGATaTGTCCAaTCACTacTGCT | Deep sequencing |
| HBB OT4 | | TTTGGGGATCTGTgCACCTCCTGAatCT | Deep sequencing |
| HBB OT5 | | TTTCGGGtTCTGTCtACTCCTTGAcaCT | Deep sequencing |
| HBB OT6 | | TTTGGaGATCTGTCCAtTCCTTcAgGCT | Deep sequencing |
| HBB OT7 | | TTTGGGGATCTGTCTgcCTtCaGcTGCT | Deep sequencing |
| HBB OT8 | | TTTGGGGATCTGTCtttgCCTtcaGCT | Deep sequencing |
| IL12A | | TTTAGGATGCCACTAAAAGGGAAAGGG | Deep sequencing |
| IL12A OT1 | | CTTGAGATGCCACTAAGAGGGAAAGGG | Deep sequencing |
| IL12A OT2 | | TTTCtGcTGCCAggAAATAGGGAAAGGG | Deep sequencing |
| IL12A OT3 | | TTTCaGATGCggCTAAAAGGGAATAGGa | Deep sequencing |
| IL12A OT4 | | TTTAGGATcCCAggAAAAAGGGAcAGGG | Deep sequencing |
| IL12A OT5 | | TTTGGtgTGgCAaTAAAAGGGAAAGGG | Deep sequencing |
| IL12A OT6 | | TTTAGGATcCCAgTAAAAaGGAAAGcAG | Deep sequencing |
| IL12A OT7 | | TTTCGcATGCCAtTAAAAAcGGAgAGGG | Deep sequencing |
| IL12A OT8 | | TTTGGGAaGaCAgTAAAAGGtAGAAGGG | Deep sequencing |
| POLQ | | TTTAGGCATGAATTATAATGCTGTTGG | Deep sequencing |
| POLQ OT1 | | TTCAGACATGAATTATAATGCTGTTGG | Deep sequencing |
| POLQ OT2 | | TTTAGGCATGAGTTATAGTGCTGTTGG | Deep sequencing |
| POLQ OT3 | | TTTAGGCATGAgTTATAATGCTGTcGG | Deep sequencing |
| POLQ OT4 | | TTTAGGCATGAATTATAgTGCTtTTGG | Deep sequencing |
| POLQ OT5 | | TTTAGGCATGAATTATAgTGCTGTTGc | Deep sequencing |
| POLQ OT6 | | TTTAGGCATGAATTATAgTGCTGTTGG | Deep sequencing |
| POLQ OT7 | | TTTAGGCATGAgTTATAgTGCTGgTGG | Deep sequencing |
| POLQ OT8 | | TTTAGGCATGAATTATgATtCaGTTGG | Deep sequencing |
| POLQ OT9 | | TTTAGGCAcGAgTTATAgTGCTGTTGG | Deep sequencing |
| POLQ OT10 | | TTTAGGCATaAATTATAgTGCTGTTGa | Deep sequencing |
| B2M | | TTTACTCACGTCATCCAGCAGAGAATG | Deep sequencing |
| B2M OT1 | | TTTCCTCACGTgAcCCAGCAcAGAACCTG | Deep sequencing |
| B2M OT2 | | TTTCCcCACtTCATCCAGCAtAGAtTG | Deep sequencing |
| B2M OT3 | | TTTGCTCACGgtgTCCAGCAGtGgATG | Deep sequencing |
| B2M OT4 | | TTTCCTCACGTgAcCCAGCAcAGAAcc | Deep sequencing |
| B2M OT5 | | TTTGCTCtCGTCAcCCAGgctAGAATG | Deep sequencing |
| B2M OT6 | | TTTCCTCACcTgATCCtGCAGAtgAgG | Deep sequencing |
| B2M OT7 | | TTTACTCACGTgcTCCtGCAGgGAAca | Deep sequencing |
|  | |  |  |
|  | |  |  |
|  | |  |  |
|  | |  |  |
|  | |  |  |
|  | |  |  |

**Additional file 2: Table S4.** Oligonucleotides (oligos) for Cas12a gene synthesis.

| pcDNA3.1 -Cas12a-NLS-3HA |
| --- |
| pET-28a-Cas12a-6HIS |
| NLS AAGCGCCCCGCCGCCACCAAGAAGGCCGGCCAGGCCAAGAAGAAGAAG |
|  |
| 3HA TACCCCTACGACGTGCCCGACTACGCCTACCCCTACGACGTGCCCGACTACGCCTACCCCTACGACGTGCCCGACTACGCC |
| 6HIS CACCACCACCACCACCAC |
| BfCas12a  TACTACGAGAGCCTGACCAAGCTGTACCCCATCAAGAAGACCATCCGCAACGAGCTGGTGCCCATCGGCAAGACCCTGGAGAACATCAAGAAGAACAACATCCTGGAGGCCGACGAGGACCGCAAGATCGCCTACATCCGCGTGAAGGCCATCATGGACGACTACCACAAGCGCCTGATCAACGAGGCCCTGAGCGGCTTCGCCCTGATCGACCTGGACAAGGCCGCCAACCTGTACCTGAGCCGCAGCAAGAGCGCCGACGACATCGAGAGCTTCAGCCGCTTCCAGGACAAGCTGCGCAAGGCCATCGCCAAGCGCCTGCGCGAGCACGAGAACTTCGGCAAGATCGGCAACAAGGACATCATCCCCCTGCTGCAGAAGCTGAGCGAGAACGAGGACGACTACAACGCCCTGGAGAGCTTCAAGAACTTCTACACCTACTTCGAGAGCTACAACGACGTGCGCCTGAACCTGTACAGCGACAAGGAGAAGAGCAGCACCGTGGCCTACCGCCTGATCAACGAGAACCTGCCCCGCTTCCTGGACAACATCCGCGCCTACGACGCCGTGCAGAAGGCCGGCATCACCAGCGAGGAGCTGAGCAGCGAGGCCCAGGACGGCCTGTTCCTGGTGAACACCTTCAACAACGTGCTGATCCAGGACGGCATCAACACCTACAACGAGGACATCGGCAAGCTGAACGTGGCCATCAACCTGTACAACCAGAAGAACGCCAGCGTGCAGGGCTTCCGCAAGGTGCCCAAGATGAAGGTGCTGTACAAGCAGATCCTGAGCGACCGCGAGGAGAGCTTCATCGACGAGTTCGAGAGCGACACCGAGCTGCTGGACAGCCTGGAGAGCCACTACGCCAACCTGGCCAAGTACTTCGGCAGCAACAAGGTGCAGCTGCTGTTCACCGCCCTGCGCGAGAGCAAGGGCGTGAACGTGTACGTGAAGAACGACATCGCCAAGACCAGCTTCAGCAACGTGGTGTTCGGCAGCTGGAGCCGCATCGACGAGCTGATCAACGGCGAGTACGACGACAACAACAACCGCAAGAAGGACGAGAAGTACTACGACAAGCGCCAGAAGGAGCTGAAGAAGAACAAGAGCTACACCATCGAGAAGATCATCACCCTGAGCACCGAGGACGTGGACGTGATCGGCAAGTACATCGAGAAGCTGGAGAGCGACATCGACGACATCCGCTTCAAGGGCAAGAACTTCTACGAGGCCGTGCTGTGCGGCCACGACCGCAGCAAGAAGCTGAGCAAGAACAAGGGCGCCGTGGAGGCCATCAAGGGCTACCTGGACAGCGTGAAGGACTTCGAGCGCGACCTGAAGCTGATCAACGGCAGCGGCCAGGAGCTGGAGAAGAACCTGGTGGTGTACGGCGAGCAGGAGGCCGTGCTGAGCGAGCTGAGCGGCATCGACAGCCTGTACAACATGACCCGCAACTACCTGACCAAGAAGCCCTTCAGCACCGAGAAGATCAAGCTGAACTTCAACAAGCCCACCTTCCTGGACGGCTGGGACTACGGCAACGAGGAGGCCTACCTGGGCTTCTTCATGATCAAGGAGGGCAACTACTTCCTGGCCGTGATGGACGCCAACTGGAACAAGGAGTTCCGCAACATCCCCAGCGTGGACAAGAGCGACTGCTACAAGAAGGTGATCTACAAGCAGATCAGCAGCCCCGAGAAGAGCATCCAGAACCTGATGGTGATCGACGGCAAGACCGTGAAGAAGAACGGCCGCAAGGAGAAGGAGGGCATCCACAGCGGCGAGAACCTGATCCTGGAGGAGCTGAAGAACACCTACCTGCCCAAGAAGATCAACGACATCCGCAAGCGCCGCAGCTACCTGAACGGCGACACCTTCAGCAAGAAGGACCTGACCGAGTTCATCGGCTACTACAAGCAGCGCGTGATCGAGTACTACAACGGCTACAGCTTCTACTTCAAGAGCGACGACGACTACGCCAGCTTCAAGGAGTTCCAGGAGGACGTGGGCCGCCAGGCCTACCAGATCAGCTACGTGGACGTGCCCGTGAGCTTCGTGGACGACCTGATCAACAGCGGCAAGCTGTACCTGTTCCGCGTGTACAACAAGGACTTCAGCGAGTACAGCAAGGGCCGCCTGAACCTGCACACCCTGTACTTCAAGATGCTGTTCGACGAGCGCAACCTGAAGAACGTGGTGTACAAGCTGAACGGCCAGGCCGAGGTGTTCTACCGCCCCAGCAGCATCAAGAAGGAGGAGCTGATCGTGCACCGCGCCGGCGAGGAGATCAAGAACAAGAACCCCAAGCGCGCCGCCCAGAAGCCCACCCGCCGCCTGGACTACGACATCGTGAAGGACCGCCGCTACAGCCAGGACAAGTTCATGCTGCACACCAGCATCATCATGAACTTCGGCGCCGAGGAGAACGTGAGCTTCAACGACATCGTGAACGGCGTGCTGCGCAACGAGGACAAGGTGAACGTGATCGGCATCGACCGCGGCGAGCGCAACCTGCTGTACGTGGTGGTGATCGACCCCGAGGGCAAGATCCTGGAGCAGCGCAGCCTGAACTGCATCACCGACAGCAACCTGGACATCGAGACCGACTACCACCGCCTGCTGGACGAGAAGGAGAGCGACCGCAAGATCGCCCGCCGCGACTGGACCACCATCGAGAACATCAAGGAGCTGAAGGCCGGCTACCTGAGCCAGGTGGTGCACATCGTGGCCGAGCTGGTGCTGAAGTACAACGCCATCATCTGCCTGGAGGACCTGAACTTCGGCTTCAAGCGCGGCCGCCAGAAGGTGGAGAAGCAGGTGTACCAGAAGTTCGAGAAGATGCTGATCGACAAGCTGAACTACCTGGTGATGGACAAGAGCCGCGAGCAGCTGAGCCCCGAGAAGATCAGCGGCGCCCTGAACGCCCTGCAGCTGACCCCCGACTTCAAGAGCTTCAAGGTGCTGGGCAAGCAGACCGGCATCATCTACTACGTGCCCGCCTACCTGACCAGCAAGATCGACCCCATGACCGGCTTCGCCAACCTGTTCTACGTGAAGTACGAGAACGTGGACAAGGCCAAGGAGTTCTTCAGCAAGTTCGACAGCATCAAGTACAACAAGGACGGCAAGAACTGGAACACCAAGGGCTACTTCGAGTTCGCCTTCGACTACAAGAAGTTCACCGACCGCGCCTACGGCCGCGTGAGCGAGTGGACCGTGTGCACCGTGGGCGAGCGCATCATCAAGTTCAAGAACAAGGAGAAGAACAACAGCTACGACGACAAGGTGATCGACCTGACCAACAGCCTGAAGGAGCTGTTCGACAGCTACAAGGTGACCTACGAGAGCGAGGTGGACCTGAAGGACGCCATCCTGGCCATCGACGACCCCGCCTTCTACCGCGACCTGACCCGCCGCCTGCAGCAGACCCTGCAGATGCGCAACAGCAGCTGCGACGGCAGCCGCGACTACATCATCAGCCCCGTGAAGAACAGCAAGGGCGAGTTCTTCTGCAGCGACAACAACGACGACACCACCCCCAACGACGCCGACGCCAACGGCGCCTTCAACATCGCCCGCAAGGGCCTGTGGGTGCTGAACGAGATCCGCAACAGCGAGGAGGGCAGCAAGATCAACCTGGCCATGAGCAACGCCCAGTGGCTGGAGTACGCCCAGGACAACACCATC |
| CeCas12a  AACAACAACACCAACAACAGCTTCGAGCCCTTCATCGGCGGCAACAGCGTGAGCAAGACCCTGCGCAACGAGCTGCGCGTGGGCAGCGAGTACACCGGCAAGCACATCAAGGAGTGCGCCATCATCGCCGAGGACGCCGTGAAGGCCGAGAACCAGTACATCGTGAAGGAGATGATGGACGACTTCTACCGCGACTTCATCAACCGCAAGCTGGACGCCCTGCAGGGCATCAACTGGGAGCAGCTGTTCGACATCATGAAGAAGGCCAAGCTGGACAAGAGCAACAAGGTGAGCAAGGAGCTGGACAAGATCCAGGAGAGCACCCGCAAGGAGATCGTGAAGATCTTCAGCAGCGACCCCATCTACAAGGACATGCTGAAGGCCGACATGATCAGCAAGATCCTGCCCGAGTACATCGTGGACAAGTACGGCGACGCCGCCAGCCGCATCGAGGCCGTGAAGGTGTTCTACGGCTTCAGCGGCTACTTCATCGACTTCTGGGCCAGCCGCAAGAACGTGTTCAGCGACAAGAACATCGCCAGCGCCATCCCCCACCGCATCGTGAACGTGAACGCCCGCATCCACCTGGACAACATCACCGCCTTCAACCGCATCGCCGAGATCGCCGGCGACGAGGTGGCCGGCATCGCCGAGGACGCCTGCGCCTACCTGCAGAACATGAGCCTGGAGGACGTGTTCACCGGCGCCTGCTACGGCGAGTTCATCTGCCAGAAGGACATCGACCGCTACAACAACATCTGCGGCGTGATCAACCAGCACATGAACCAGTACTGCCAGAACAAGAAGATCAGCCGCAGCAAGTTCAAGATGGAGCGCCTGCACAAGCAGATCCTGTGCCGCAGCGAGAGCGGCTTCGAGATCCCCATCGGCTTCCAGACCGACGGCGAGGTGATCGACGCCATCAACAGCTTCAGCACCATCCTGGAGGAGAAGGACATCCTGGACCGCCTGCGCACCCTGAGCCAGGAGGTGACCGGCTACGACATGGAGCGCATCTACGTGAGCAGCAAGGCCTTCGAGAGCGTGAGCAAGTACATCGACCACAAGTGGGACGTGATCGCCAGCAGCATGTACAACTACTTCAGCGGCGCCGTGCGCGGCAAGGACGACAAGAAGGACGCCAAGATCCAGACCGAGATCAAGAAGATCAAGAGCTGCAGCCTGCTGGACCTGAAGAAGCTGGTGGACATGTACTACAAGATGGACGGCATGTGCCTGGAGCACGAGGCCACCGAGTACGTGGCCGGCATCACCGAGATCCTGGTGGACTTCAACTACAAGACCTTCGACATGGACGACAGCGTGAAGATGATCCAGAACGAGCACATGATCAACGAGATCAAGGAGTACCTGGACACCTACATGAGCATCTACCACTGGGCCAAGGACTTCATGATCGACGAGCTGGTGGACCGCGACATGGAGTTCTACAGCGAGCTGGACGAGATCTACTACGACCTGAGCGACATCGTGCCCCTGTACAACAAGGTGCGCAACTACGTGACCCAGAAGCCCTACAGCCAGGACAAGATCAAGCTGAACTTCGGCAGCCCCACCCTGGCCAACGGCTGGAGCAAGAGCAAGGAGTTCGACAACAACGTGGTGGTGCTGCTGCGCGACGAGAAGATCTACCTGGCCATCCTGAACGTGGGCAACAAGCCCAGCAAGGACATCATGGCCGGCGAGGACCGCCGCCGCAGCGACACCGACTACAAGAAGATGAACTACTACCTGCTGCCCGGCGCCAGCAAGACCCTGCCCCACGTGTTCATCAGCAGCAACGCCTGGAAGAAGAGCCACGGCATCCCCGACGAGATCATGTACGGCTACAACCAGAACAAGCACCTGAAGAGCAGCCCCAACTTCGACCTGGAGTTCTGCCGCAAGCTGATCGACTACTACAAGGAGTGCATCGACAGCTACCCCAACTACCAGATCTTCAACTTCAAGTTCGCCGCCACCGAGACCTACAACGACATCAGCGAGTTCTACAAGGACGTGGAGCGCCAGGGCTACAAGATCGAGTGGAGCTACATCAGCGAGGACGACATCAACCAGATGGACCGCGACGGCCAGATCTACCTGTTCCAGATCTACAACAAGGACTTCGCCCCCAACAGCAAGGGCATGCAGAACCTGCACACCCTGTACCTGAAGAACATCTTCAGCGAGGAGAACCTGAGCGACGTGGTGATCAAGCTGAACGGCGAGGCCGAGCTGTTCTTCCGCAAGAGCAGCATCCAGCACAAGCGCGGCCACAAGAAGGGCAGCGTGCTGGTGAACAAGACCTACAAGACCACCGAGAAGACCGAGAACGGCCAGGGCGAGATCGAGGTGATCGAGAGCGTGCCCGACCAGTGCTACCTGGAGCTGGTGAAGTACTGGAGCGAGGGCGGCGTGGGCCAGCTGAGCGAGGAGGCCAGCAAGTACAAGGACAAGGTGAGCCACTACGCCGCCACCATGGACATCGTGAAGGACCGCCGCTACACCGAGGACAAGTTCTTCATCCACATGCCCATCACCATCAACTTCAAGGCCGACAACCGCAACAACGTGAACGAGAAGGTGCTGAAGTTCATCGCCGAGAACGACGACCTGCACGTGATCGGCATCGACCGCGGCGAGCGCAACCTGCTGTACGTGAGCGTGATCGACAGCCGCGGCCGCATCGTGGAGCAGAAGAGCTTCAACATCGTGGAGAACTACGAGAGCAGCAAGAACGTGATCCGCCGCCACGACTACAAGGGCAAGCTGGTGAACAAGGAGCACTACCGCAACGAGGCCCGCAAGAGCTGGAAGGAGATCGGCAAGATCAAGGAGATCAAGGAGGGCTACCTGAGCCAGGTGATCCACGAGATCAGCAAGCTGGTGCTGAAGTACAACGCCATCATCGTGATGGAGGACCTGAACTACGGCTTCAAGCGCGGCCGCTTCAAGGTGGAGCGCCAGGTGTACCAGAAGTTCGAGACCATGCTGATCAACAAGCTGGCCTACCTGGTGGACAAGAGCCGCGCCGTGGACGAGCCCGGCGGCCTGCTGAAGGGCTACCAGCTGACCTACGTGCCCGACAACCTGGGCGAGCTGGGCAGCCAGTGCGGCATCATCTTCTACGTGCCCGCCGCCTACACCAGCAAGATCGACCCCGTGACCGGCTTCGTGGACGTGTTCGACTTCAAGGCCTACAGCAACGCCGAGGCCCGCCTGGACTTCATCAACAAGCTGGACTGCATCCGCTACGACGCCAGCCGCAACAAGTTCGAGATCGCCTTCGACTACGGCAACTTCCGCACCCACCACACCACCCTGGCCAAGACCAGCTGGACCATCTTCATCCACGGCGACCGCATCAAGAAGGAGCGCGGCAGCTACGGCTGGAAGGACGAGATCATCGACATCGAGGCCCGCATCCGCAAGCTGTTCGAGGACACCGACATCGAGTACGCCGACGGCCACAACCTGATCGGCGACATCAACGAGCTGGAGAGCCCCATCCAGAAGAAGTTCGTGGGCGAGCTGTTCGACATCATCCGCTTCACCGTGCAGCTGCGCAACAGCAAGAGCGAGAAGTACGACGGCACCGAGAAGGAGTACGACAAGATCATCAGCCCCGTGATGGACGAGGAGGGCGTGTTCTTCACCACCGACAGCTACATCCGCGCCGACGGCACCGAGCTGCCCAAGGACGCCGACGCCAACGGCGCCTACTGCATCGCCCTGAAGGGCCTGTACGACGTGCTGGCCGTGAAGAAGTACTGGAAGGAGGGCGAGAAGTTCGACCGCAAGCTGCTGGCCATCACCAACTACAACTGGTTCGACTTCATCCAGAACCGCCGCTTC |
| PrCas12a  ATCGACCTGAAGCAGTTCATCGGCATCTACCCCGTGAGCAAGACCCTGCGCTTCGAGCTGCGCCCCGTGGGCAAGACCCAGGAGTGGATCGAGAAGAACCGCGTGCTGGAGGGCGACGAGCAGAAGGCCGCCGACTACCCCGTGGTGAAGAAGCTGATCGACGACTACCACAAGGTGTGCATCCACGACAGCCTGAACCACGTGCACTTCGACTGGGAGCCCCTGAAGGACGCCATCGAGATCTTCCAGAAGACCAAGAGCGACGAGGCCAAGAAGCGCCTGGAGGCCGAGCAGGCCATGATGCGCAAGAAGATCGCCGCCGCCATCAAGGACTTCAAGCACTTCAAGGAGCTGACCGCCGCCACCCCCAGCGACCTGATCACCAGCGTGCTGCCCGAGTTCAGCGACGACGGCAGCCTGAAGAGCTTCCGCGGCTTCGCCACCTACTTCAGCGGCTTCCAGGAGAACCGCAACAACATCTACAGCCAGGAGGCCATCAGCACCGGCGTGCCCTACCGCCTGGTGCACGACAACTTCCCCAAGTTCCTGAGCGACCTGGAGGTGTTCGAGCGCATCAAGAGCACCTGCCCCGAGGTGATCAACCAGGCCAGCGCCGAGCTGCAGCCCTTCCTGGAGGGCGTGATGATCGACGACATCTTCAGCCTGGACTTCTACAACAGCCTGCTGACCCAGAACGGCATCGACTTCTTCAACCAGGTGATCGGCGGCGTGAGCGAGAAGGACAAGCAGAAGTACCGCGGCATCAACGAGTTCAGCAACCTGTACCGCCAGCAGCACAAGGAGATCGCCGCCAGCAAGAAGGCCATGACCATGATCCCCCTGTTCAAGCAGATCCTGAGCGACCGCGACACCCTGAGCTACATCCCCGCCCAGATCCGCACCGAGGACGAGCTGGTGAGCAGCATCACCCAGTTCTACGACCACATCACCCACTTCGAGCACGACGGCAAGACCATCAACGTGCTGAGCGAGATCGTGGCCCTGCTGGGCAAGCTGGACACCTACGACCCCAACGGCATCTGCATCACCGCCCGCAAGCTGACCGACATCAGCCAGAAGGTGTACGGCAAGTGGAGCGTGATCGAGGAGAAGATGAAGGAGAAGGCCATCCAGCAGTACGGCGACATCAGCGTGGCCAAGAACAAGAAGAAGGTGGACGCCTTCCTGAGCCGCAAGGCCTACAGCCTGAGCGACCTGTGCTTCGACGAGGAGATCAGCTTCAGCCGCTACTACAGCGAGCTGCCCCAGACCCTGAACGCCATCAGCGGCTACTGGCTGCAGTTCAACGAGTGGTGCAAGAGCGACGAGAAGCAGAAGTTCCTGAACAACCAGACCGGCACCGAGGTGGTGAAGAGCCTGCTGGACGCCATGATGGAGCTGTTCCACAAGTGCAGCGTGCTGGTGATGCCCGAGGAGTACGAGGTGGACAAGAGCTTCTACAACGAGTTCCTGCCCCTGTACGAGGAGCTGGACACCCTGTTCCTGCTGTACAACAAGGTGCGCAACTACCTGACCCAGAAGCCCAGCGACGTGAAGAAGTTCAAGCTGAACTTCGAGAGCCCCAGCCTGGCCAGCGGCTGGGACCAGAACAAGGAGATGAAGAACAACGCCATCCTGCTGTTCAAGGACGGCAAGAGCTACCTGGGCGTGCTGAACGCCAAGAACAAGGCCAAGATCAAGGACGCCAAGGGCGACGTGAGCAGCAGCAGCTACAAGAAGATGATCTACAAGCTGCTGAGCGACCCCAGCAAGGACCTGCCCCACAAGATCTTCGCCAAGGGCAACCTGGACTTCTACAAGCCCAGCGAGTACATCCTGGAGGGCCGCGAGCTGGGCAAGTACAAGAAGGGCCCCAACTTCGACAAGAAGTTCCTGCACGACTTCATCGACTTCTACAAGGCCGCCATCAGCATCGACCCCGACTGGAGCAAGTTCAACTTCCAGTACAGCCCCACCGAGAGCTACGACGACATCGGCATGTTCTTCAGCGAGATCAAGAAGCAGGCCTACAAGATCCGCTTCACCGACATCAGCGAGGCCCAGGTGAACGAGTGGGTGGACAACGGCCAGCTGTACCTGTTCCAGCTGTACAACAAGGACTACGCCGAGGGCGCCCACGGCCGCAAGAACCTGCACACCCTGTACTGGGAGAACCTGTTCACCGACGAGAACCTGAGCAACCTGGTGCTGAAGCTGAACGGCCAGGCCGAGCTGTTCTGCCGCCCCCAGAGCATCAAGAAGCCCGTGAGCCACAAGATCGGCAGCAAGATGCTGAACCGCCGCGACAAGAGCGGCATGCCCATCCCCGAGAGCATCTACCGCAGCCTGTACCAGTACTACAACGGCAAGAAGAAGGAGAGCGAGCTGACCGTGGCCGAGAAGCAGTACATCGACCAGGTGATCGTGAAGGACGTGACCCACGAGATCATCAAGGACCGCCGCTACACCCGCCAGGAGTACTTCTTCCACGTGCCCCTGACCTTCAACGCCAACGCCGACGGCAACGAGTACATCAACGAGCACGTGCTGAACTACCTGAAGGACAACCCCGACGTGAACATCATCGGCATCGACCGCGGCGAGCGCCACCTGATCTACCTGACCCTGATCAACCAGCGCGGCGAGATCCTGAAGCAGAAGACCTTCAACGTGGTGAACAGCTACAACTACCAGGCCAAGCTGGAGCAGCGCGAGAAGGAGCGCGACGAGGCCCGCAAGAGCTGGGACAGCGTGGGCAAGATCAAGGACCTGAAGGAGGGCTTCCTGAGCGCCGTGATCCACGAGATCACCAACATGATGATCGAGAACAACGCCATCGTGGTGCTGGAGGACCTGAACTTCGGCTTCAAGCGCGGCCGCTTCAAGGTGGAGCGCCAGGTGTACCAGAAGTTCGAGAAGATGCTGATCGACAAGCTGAACTACCTGAGCTTCAAGGACCGCGAGGCCGGCGAGGAGGGCGGCATCCTGCGCGGCTACCAGATGGCCCAGAAGTTCATCAGCTTCCAGCGCCTGGGCAAGCAGAGCGGCTTCCTGTTCTACATCCCCGCCGCCTACACCAGCAAGATCGACCCCGTGAGCGGCTTCGTGAACCACTTCAACTTCAGCGACATCACCAACGCCGAGAAGCGCAAGGACTTCCTGATGAAGATGGACCGCATCGAGATGAAGAACGGCAACATCGAGTTCACCTTCGACTACCGCAAGTTCAAGACCTTCCAGACCGACTACCAGAACGTGTGGACCGTGAGCACCTTCGGCAAGCGCATCGTGATGCGCATCGACGAGAAGGGCTACAAGAAGATGGTGGACTACGAGCCCACCAACGACATCATCAAGGCCTTCAAGAACAAGGGCATCCTGCTGAGCGAGGGCAGCGACCTGAAGGCCCTGATCGCCGAGATCGAGGCCAACGCCACCAACGCCGGCTTCTACAGCACCCTGCTGTACGCCTTCCAGAAGACCCTGCAGATGCGCAACAGCAACGCCGTGACCGAGGAGGACTACATCCTGAGCCCCGTGGCCAAGGACGGCCACCAGTTCTGCAGCACCGACGAGGCCAACAAGGGCAAGGACGCCCAGGGCAACTGGGTGAGCAAGCTGCCCGTGGACGCCGACGCCAACGGCGCCTACCACATCGCCCTGAAGGGCCTGTACCTGCTGCGCAACCCCGAGACCAAGAAGATCGAGAACGAGAAGTGGCTGCAGTTCATGGTGGAGAAGCCCTACCTGGAG |
| CsbCas12a  TTCGACCAGTTCACCAACATCTACTCTCTGGACAAAACCCTGCGTTTCAAACTGAACCCGGTTGGTAACACCCCGGACCTGCTGGAACAGAACCAGGTTCTGAAAAAAGACCAGACCATCGAAAACTCTTACCAGCAGGCTAAACCGTACCTGGACGAACTGCACCGTCGTCTGATCAACGAAGCTCTGACCCCGGAAAACCTGCGTAACTTCCCGTTCAACGAATACGCTGCTGCTTACGAATACATCAAAGAAATCAACCGTAACCGTTACTCTGCTAACAACAAAAAAGAAACCGCTGCTTCTAACGAATGGGAATCTAAAAAATCTGACTTCCGTAAAGCTGTTGTTGACATGCTGAACCAGCAGGCTGACCACTGGAAACAGAAATACCAGGACCTGGAATTCAACAAAGGTGAACTGGAACGTAAAGGTACCAACTTCCTGACCTCTCCGGCTATCAACAAAATCCTGAAAACCGAATTCCCGCCGGAAAAAGAACAGGAACTGGTTGACAAAGGTTTCCCGTCTCTGCACGTTGACGAAGAAGAAAACTCTGGTGAAAAACGTTACATCTTCGAATCTTTCGACAAATTCGCTACCTACCTGTCTCGTTTCCAGCAGACCCGTCAGAACCTGTACACCTCTGAAGACAAAGCTACCGCTATCGCTACCCGTGTTGTTGCTAACTTCACCACCTTCATGCAGAACATCCACGAATTCCGTGACAAACACTCTTCTATCGAAGACGAACTGAACCTGTCTCAGGACGAAAAACGTATCTTCGAACCGGTTTCTTACCGTCACTACGTTCTGCAGGCTGACATCGAAGAATACAACGCTGTTATCGGTGACATCAACAAACGTATGAAAGAACTGCGTGACCAGAAACACAAAAACCCGAACTACAAAAAAACCGACTACCCGCTGCTGTCTACCCTGGACAAACAGATCCTGGGTCTGCAGGAAAAAGAACACCAGCTGATCGAAGACGATGAGGACGTCTGGCCGTGCGTGGAAGAACTGCTGAACATCTCTGAACAGCACTTCCCGGCTCTGCAGAAATCTGTTAACGCTCTGTCTGAAACCTTCTTCGAACCGGAACTGCCGAACATCTACCTGAAAGACAAAAACGTTAACACCATCTCTAACCGTTGGTTCGTTAACGGTGACGACTTCCTGATCCGTCTGCCGCAGAAAAACAAAAAAAAAGACGAAAAAGACACCCCGAAAATCAAAACCTTCATCTCTCTGCAGGACGTTCGTACCGCTATCGACGACATGGAAGGTGTTCTGTTCAAAGACCGTTTCTACGAAGAAGGTGCTATCTCTCCGGACCAGTCTACCTGGCAGCAGTTCCTGGGTATCTTCCAGCACGAACTGTCTAACGCTATGGAAGAATACTACCAGTCTGCTCGTTCTCTGCGTGAAGTTAAAGACGCTGATCCTGCGTTCGACAAAGAACATCACACCCCGGTTATCAAAGAATTCGCTGACGCTTGCCTCCGTGTATACAGACTGCTGGACTACTTCGCTCTGACCCACCGTCAGTCTTCTCAGATCCCGGACGTTTTCTCTACCGAATTCTACGAAGAATTCGACGCTCACTTCTACGAAGTTAACGTTCCGCGTTACTACAACGCTCTGCGTGACTACATAGCTCAGGTCTTCTACGGGGAAGACAAAATCAAACTGAACTTCGGTAAAGGTAACCTGCTGGGTGGTTGGTCTGAATCTCGTCGTAACGGTGCTCAGTACTGCGGTTACATCCTGCGTCGTCCGCAGACCAACACCTACTACCTGGCTATCACCGACAACCCGTGGATCCTGGACACCGAAAAACACTCTGAAATCAAAGACACCTCTAACGGTATGTACGAAAAAATGGAATACTCTCAGCTGAAAGCTCAGACCATCTACGGTCCGTCTTATGAGGGTGAGTTCGGGGTGAGCTACGACACCGACAAACAGAACTCTACCAACCAGAAAATCATCGAACGTGTTAAACGTCTGCTGCAGAAAAACTTCGTTGAACAGTACCCGGAACTGCAGACCATCATCGACCGTGAATACTCTGACGCTGACGCTCTGGCTCGTGAAGTTTCTAACCAGAACCTGTACCGTATCTCTTTCGTTCCGGTTTCTGCTGAATACATCGAACAGGGTCGTTACGAAGCTAAACGTGGTGGTTACAACCACCTGTACATCTTCGAAATCACCAACAAAGACCTGGCTAAACCGCACTCTGGTGGTAACCCGAACCTGCACACCTCTTACTTCCTGCACCTGTTCTCTCAGGAAAACCTGTCTAACCCGGTTCTGAAACTGTCTGGTAACGCTGAAATCTTCTTCCGTCCGGGTAACCACGACCTGCCGACCAAAACCGACTCTCTGGGTAAACAGGTTACCACCCACAAACGTTACTCTAAAGACACCCTGCTGTTCCACCTGCCGGTTTCTATCAACTTCAAAAAAGGTTCTATGAAACCGAAACAGTTCAACGACATCACCAACCAGAAAATCGCTGACCAGCCGAAAGACAACCTGAACATCATCGGTATCGACCGTGGTGAAAAGCATCTGGTATACTACAGCGTTGTTGACTGCCACGGTCGTATCCTGGACCAGGGTTCTCTGAACGAAATCAACGGTATCGACTACCACCGTCTGCTGGACGAACGTGAAAAAGAACGTATCAAAAACCGTCAGTCTTGGGAACCGATCGAAGACATCAAAAACCTGAAAAAAGGTTACATCTCTCACGTTGTTCACGCTCTGTCTCGTCTGGCTGTTGAACACAACGCTATCATCGTTATGGAAGACCTGAACATGCGTTTCAAACAGATCCGTGGTGGTATCGAAAAAGGTACCTACCAGCGTCTGGAACGTCAGCTGATCGACAAACTGAACTACCTGGTTTTCAAAGACCGTGACGCTCGTGAAACCGGTGGTATCCTGCGTGGTTACCAGCTGACCGCTCCGTTCGAATCTTTCGAAAAAATGGGTAAACAGACCGGTACCATCTTCTACACCGAAGCTGGTTACACCTCTGTTACCGACCCGCTCACTGGCTTCCGTAAAAACATATACCTGAAAAACTCTGACACCGTTGAAAACCTGAAACAGGCTATCAACACCTTCCACACCATCGAATGGGACGAACAGCGTGAATCTTACTACTTCCGTTACGACGTTGCTGACTTCTCTAAATCTAAAGACACCCCGTCTCGTATCTGGGAAGTTTACGCTAACGTTCCGCGTATCATCCGTAAAAAAGTTGACGAACGTTGGACCGCTGCTCCGGTTAACCCGAACGACCTGCTGGCTGAACTGCTGCAGACCTACGACTTCGAAAACCCGTACGGTGACGTTCTGGAACAGATCCGTATCAAAGAAGAAGCTGGTGAACTGAAAGGTGAACGTGAATTCGACGGTGAACAGCGTAACTTCTACAAATCTCTGGTTTACATCTTCAACCTGATCCTGCAGCTGCGTAACTCTATGTCTCACAAATACCGTGTTGAAAACGACTCTGTTATCACCGAAGGTGAAGACCTGGACTTCATCGCTTCTCCGGTTCCGCCGTTCTTCACCACCGAATCTTCTTACTCTACCGCTAACTTTGGCGGCTTCGAACAGCGTTTTATCGGTAACCCGCAGGCTCGTGAAAAATTCATGGAAGAATTCAACGGTGACGCTAACGGTGCTTACAACATCGCTCGTAAAGGTGTTCTGCTGCTGGACCGTGTTCGTGAAAACCCGTCTAACCCGGACATCTTCATCACCAACCAGGAATGGGACCGTGCTGCTACCGAATGGGACACCTCT |
| BhCas12a  CTGTACGACGAATACTCTACCCAGTACTACAAAGGTGACGAAAAACGTCCGATGCTGAACTACGAAGAATTCACCAAACGTAACCACATCACCAAAGCTCTGCGTATGGAACTGATCCCGCAGGGTAAAACCCAGAACGTTATCGACGAAAAAGGTGACCGTAAATACGACGCTGCTCTGTACTCTTCTCTGGAACGTCTGAAACCGGTTATCGACTCTTTCATCCGTTCTACCGCTTCTCGTGCTCTGTCTGACGTTGACTACGACTTCAACGCTATGCACGACGCTTACATCAACAAAGACAAAAAATCTTGGGCTAAAGAAGAAAAAGCTCTGAAAAAAGTTCTGATGAAAGCTGTTGACGAAGCTCTGCCGAAAGGTCTGAAATGCTCTCAGATCAATTCGGCTGCGTTCCTGCAGGAAGTTCTGCGTGAATACGTTCTGCACGCTACCGACACCGAACTGCGTAAAGACGTTGCTCTGAAAGACATCGAAGAAACCAAAGGTTGCCTGGCTCTGTTCTCTAAATTCCTGACCACCCGTATCACCGCTCTGACCGTTTGGATGCCGGAACGTGTTATCGAAAACTTCAAAATCTACTGCTCTAACATCCCGCGTATCGAAGCTATCTTCAACGAAGCTAAAGACATCGCTAACAACTACTCTGACGAACTGGAACTGATGAAAACCGCTCAGTACTACACCAAAATCCTGTCTCAGGACGCTATCGACGGTTACAACCTGGTTATCGCTGGTAAAATCACCGAAAACGGTATCGAAACCAAAGGTCTGAACGTTCTGATCAACGAATACAACATCGACGTTAAAAACCAGAAACTGGACAAACCGTACCTGCGTAAAATCAACCAGCTGTACAAACAGACCCTGTTCTCTTCTGAAAAACAGTTCGTTATCACCGCTATCAAAACCGACGACGAAGTTCGTCGTGTTATCAAATCTGCTTGGGAATCTTTCGACGGTGCTGCTACCAAAATGCTGGGTCTGTTCAAAGAAACCCTGGAAGCTACCAATGGCAACGGTGTATGCGTGAAAGGTAACCGTCTGCACATCCTGTCTCACGCTCTGCTGGGTGAACACAAAGCTATCACCGACAACCTGGTTAAAGCTGAACTGGTTGAAATCCACGAAATGCTGAAAAACGAAGCTCTGAAACCGTCTATGCGTGCTGAACTGGAAAAACGTGTTGACATCGCTCAGTCTCTGGTTGTTAAAAAAGACTACTCTTTCACCGCTCTGGACGAAGCTGTTACCTCTATCGACGAAAACGTTATCGGTCTGTCTAAAGGTGCTTTCAACCTGTACGTTGCTAAAACCGAAGAACTGATCAAAGAAGCTAAAATGTACTACAAAGTTCTGGAAGGTGGTGACATCTTCAAAAAACGTCACATCAAAGGTGACAAACACGTTCAGGAAATGCTGGTTGACTTCTTCGACGCTCTGACCGAAGTTCGTAACATCATCTCTGTTATCTCTATGCCGGACGAAAACGAAGACGCTGACGTTTCTTTCTACAACCGTTTCGACGAAATCTACGAAAACATCCGTCTGACCTACAAAGCTGAAAACCTGGTTCGTAACTACATCACCAAATCTGTTAAAGACACCGCTGAAGAAAAACAGACCTGCTTCGGTACCCCGGCTCGTCTGCGTACCCAGTGGTGGAACGGTGAACAGAAATTCGCTAAAAACCACGCTGCTATCATCAAACACGACGGTAAATACTACTACTTCATCCTGGCTGGTGACTCTAAACCGATCGAAATCAAAGAAGACGGTAACTCTGCTACCGGTCTGCTGACCCTGAAAAAAGGTCAGAAATCTTTCATGATGCTGCCGAAAATCCTGTTCACCGACCACGCTGTTCCGTTCTTCGAAGGTAACAAAGACGCTATGGAATACACCCTGGACGACGAATCTGTTATCCGTCCGGTTAAAGTTGGTCGTATGCTGTACGAAATCTACAAAAAAGGTCTGTTCAAACGTGAAGCTGTTACCTCTGGTGCTATCACCGAAGAAGAATACGCTAAAAACATCCAGGCTCTGATCGAAAAATACACCGAATTCGCTAACGCTTACGTTCAGTACCAGAAATTCAACCTGGACGACATCAACGACCCGACCCGTTACTCTGACATCGGTGAATTCTTCTCTGAAGTTGACACCTGCACCTCTCGTCTGTCTTGGACCTACATCGACTACGCTCAGATCGCTAACCTGGTTGACTCTGGTTCTGCTTACCTGTTCCTGATCTCTACCAAATTCCTGTACACCGAATCTGAAGACAAAAACGCTTACACCAAAACCTTCCGTTCTATCCTGTCTGACGCTAACATGGACAAAACCACCATCCTGCTGAACTCTAACCCGGCTGTTTTCTTCCGTCCGCAGTCTATCAAAAAAGAAATCACCCACAAAGCTGGTTCTATCATGGTTAACAAACTGACCGAAGACGGTGAACACATCCCGAAAAAAATCTACGAAGCTATCTACAAATCTAAAAACGAAATGTCTGGTGTTTCTGAAGAAGACATGGCTGCTGCTAACGAATACATGCGTACCCACAAAGTTCGTTCTTTCAAAGCTAAATACGACAAAACCTACCGTGGTAACTACATGTCTGACAAATACACCCTGCAGCTGACCTACACCAAAAACAACGACGTTTCTGACCGTGTTAACGACATGCTGAACGACCGTGTTATCGAAGCTATGCAGGACGGTTTCAACATCGTTTCTGTTGCTCGTTCTACCAAAGACATGGTTTACGCTCTGGTTCTGGACTCTTCTCTGAAAATCATCAAAGAACTGTCTCTGAACGTTATCGACGGTGTTGACTACTACGCTCTGCTGCACGACACCTACCTGGAAAAAAAAGAAAACAAAAAACTGTGGATCTACGACACCGAAAACACCGAACTGAAATCTGCTTACATCGACCTGGCTATCTCTGAAATCCTGAAACTGGCTCGTGAATACAACGCTGTTATCGCTGTTGAATCTATCTCTGACGCTGTTAAAAACAAATACTCTTTCATCGACAACCAGGTTTTCAAAGCTTTCGAAAACCGTATCGCTCAGCGTCTGTCTGACCTGACCTACAAAGACGTTGTTGACGGTCGTCCGGGTTCTGTTTCTAACCCGCTGCAGCTGTCTAACAACAACGGTAACACCTACCAGGACGGTATCCTGTTCTTCATCAACGGTGCTTACACCCGTGGTATCGACCCGTCTTCTGGTTTCACCTCTCTGTTCGACTTCTCTCGTTACAACTCTATCGCTTCTAAACGTCAGTTCTTCTCTAAAATGGCTAAAATCTCTTACACCGGTGACTCTATCGTTTTCGACTTCGACTACGTTGACTACCCGGTTCACGTTGACACCGAAAAAACCAAATGGCAGGTTAAACTGTCTGGTGACGTTGTTGTTTACGACCGTGAAAAAAAACAGAACAAACGTATCAAAGACGTTGTTAACGAAATCATCATCCCGCTGGCTGGTAAAACCGACCTGAACGGTAACATCGCTGAAAACATCCTGAACAAAGACGTTCCAGGTGCGTTCGTTGAAGAACTCTTCCGTTGGTTCCGTTACGCTGTTACCGGTATCCACGCTCAGGTTAAAGGTAAAGACGAATTCTACAAATCTCCGGTTGACGGTAACGAATACAACATCTCTAACATGCTGGCTTTCAACCTGGCTAAAAAACTGGTTTTCCGTCTGGAATACGCTGGTGAATCTAAAGACTTCACCAAAGAATGGCTGAACTACATGCAGGCT |
