# Supplementary material for: A Cas12a ortholog with stringent PAM recognition followed by low off-target editing rates for genome editing
Source: Genome Biol. 2020 Mar 25;21:78. doi: 10.1186/s13059-020-01989-2 (PMC7093978; doi:10.1186/s13059-020-01989-2)
Supplement: Supplementary file 4 — Additional file 4. Review history. [file 13059_2020_1989_MOESM4_ESM.docx]

Review History

**First round of review**

**Reviewer 1**

**Are you able to assess all statistics in the manuscript, including the appropriateness of statistical tests used?**

Yes, and I have assessed the statistics in my report

**Comments to author:**

In this manuscript by Chen and colleagues, a type V-A Cas12a ortholog derived from C. eutactus (CeCas12a) was reported to edit the human genome with lower off-target effects for its stringent PAM recognition. The authors present a characterization of this Cas12a and its use for genome editing in human cells. Specifically, the authors claim a stringent PAM recognition by CeCas12a, which results in lower off-target effects compared with previously reported AsCas12a and LbCas12a. However, the provided data is not sufficient to support the claim about PAM recognition and accuracy of CeCas12a.
Major point
1.     Valuable features for new Cas proteins encompass size, accuracy, targeting space (related to the PAM), easy delivery and in vivo editing levels. In some cases, there are standard tests to determine the PAM (PAM assays testing libraries using deep sequencing to determine depletion), and specificity (e.g. GUIDE-Seq, CIRCLE-Seq, DISCOVER-Seq, etc.). It's unclear whether the current results meet the technical and methodological standards for recent studies on Cas characterization. The authors claim "stringent PAM recognition of CeCas12a", but the in vitro data seems to be conflict with the conclusion. The results from Figs. 1, 3 and related Supplementary Figs. show that in a large degree, the cleavage patterns of CeCas12a and Bf Cas12a are similar to reported LbCas12a, FnCas12a and AsCas12a, including canonical and non-canonical PAMs. So these results may not support the conclusion that "CeCas12a presented the highest stringency on PAM recognition". The authors should carry out experiments to actually characterize and determine the new Cas12a PAM(s), which would have major implications for both efficiency and specificity (both on- and off-target would be impacted).
2.     More endogenous loci and statistical analysis should be included for measuring the nuclease activity of CeCas12a.
3.     Targeted deep sequencing of a small number of selected sites might not be strong enough to support the specificity claim. Given that CeCas12a specificity is a major point of this paper, the author should carry out a comprehensive comparative analysis using high-throughput and genome-wide methods (eg. GUIDE-Seq, etc.), as well as the high-fidelity version of Cas12a (eg. enAsCas12a-HF1). To include other Cas proteins (eg. Cas9 and Cas12b) for comparison is not essential but will be very interesting.

Minor point
1.     The authors states that they identify 5 new Cas12a proteins which have not previously been employed for genome editing. However, BfCas12a has been characterized in a preprint paper published in bioRxiv (Zetsche et al., dio.org/10.1101/134015). Thus, the statement should be rewritten, and this reference should be cited.
2.     In this manuscript, 12 Cas12b orthologs are included, but information of several proteins is omitted. Please add the description or related citation in the right place.

**Reviewer 2**

**Are you able to assess all statistics in the manuscript, including the appropriateness of statistical tests used?**

Yes, and I have assessed the statistics in my report.

**Comments to author:**

Review of Chen et al.

Chen et al. test a variety of Cas12a nucleases for DNA cleavage in vitro and gene editing in mammalian cells. They identified a new Cas12a orthologue, CeCas12a, that shows more specific PAM recognition in vitro and fewer off-target effects in vivo when compared to established Cas12a nucleases (LbCas12a and AsCas12a). Overall, CeCas12a could be a valuable new gene editing tool—especially for situations in which specificity is more important than flexibility.

Major Comments

In vitro cleavage efficiencies should be quantified for all figures. One of the authors' key arguments for the manuscript is that CeCas12a is more specific for PAM recognition than other Cas12a orthologues tested, but the reader has to compare band intensities by eye across several gels to draw that conclusion. A bar graph of efficiencies for each PAM would be a clearer Fig. 1. I would keep the gel images but move them to the supplement.

I couldn't find what statistical test was used to calculate P-values for Figs. 2, 3, and 4. Please include that information.

Minor Comments

Italicize species names throughout.

Lines 104-105: Is the cleavage assay data for other Cas12a orthologs not shown?

Line 113: I don't understand why the cleavage activity of CeCas12a and BfCas12a is surprising.

Lines 171-174: I'm confused about the purpose of the dCas12a controls—were they just confirmed in vitro?

Lines 199-200: The authors should provide quantitative range of gene editing outcomes instead of vague descriptions like "pretty well."

Lines 250-253: I recommend showing this data as a bar graph because it's difficult to follow in written form.

Fig. 4A: I recommend summarizing the important features of this table as a bar graph and moving the rest of the table to the supplement.

Lines 359-361: I'm not sure what you were trying to say here. Off target effects for CeCas12a are still determined by a combination of PAM recognition and individual spacer sequences, it's just that the spacer plays a more significant role for CeCas12a because PAM recognition is more specific.

**Authors Response**

**Point-by-point responses to the reviewers’ comments:**

Reviewer comments:
Reviewer #1
1. Response to comment: Valuable features for new Cas proteins encompass size, accuracy, targeting space (related to the PAM), easy delivery and in vivo editing levels. In some cases, there are standard tests to determine the PAM (PAM assays testing libraries using deep sequencing to determine depletion), and specificity (e.g. GUIDE-Seq, CIRCLE-Seq, DISCOVER-Seq, etc.). It's unclear whether the current results meet the technical and methodological standards for recent studies on Cas characterization.

*Response:
Thank you very much for valuable advices. We totally agree that there were standard tests to determine the PAM. The standard PAM identification assays by deep sequencing were carried out for CeCas12a and BfCas12a. We synthesized 256 kinds of linear dsDNA substrates consisting of 4 randomized nucleotides upstream of 23nt protospacer, and dsDNA substrates were mixed with each other at the same concentration. Each in vitro cleavage reaction consisted of 100 ng mixed dsDNA substrates, 100nM Cas12a RNP complex, 1 μL 10× CutSmart buffer (NEB) and water for a total volume of 10 μL. Each cleavage reaction was incubated at 37 °C and quenched at 0 min, 1 min, 2 min, 4 min, 6 min, 8 min, 10 min, 16 min, 32 min. Reactions were purified with magnetic beads (VAHTS DNA Clean Beads, Vazyme) and 10 ng purified dsDNA was amplified with PCR over 20 total cycles using custom primers containing Illumina adaptors. The unmodified library amplicon was used as a negative control. Purified PCR products were quantified and prepared for sequencing with a 75-cycle NextSeq kit (Illumina). The reads of PAM regions were extracted, counted, and normalized to total reads for each sample. The result has been put in the revised manuscript. The GUIDE-Seq was also performed and results are shown in Response Figure 3 and 4.

Response Figure 1. PAM identification assay (revised manuscript: Fig. 1B, C, D)*

2. Response to comment: The authors claim "stringent PAM recognition of CeCas12a", but the in vitro data seems to be conflict with the conclusion. The results from Figs. 1, 3 and related Supplementary Figs. show that in a large degree, the cleavage patterns of CeCas12a and Bf Cas12a are similar to reported LbCas12a, FnCas12a and AsCas12a, including canonical and non-canonical PAMs. So these results may not support the conclusion that "CeCas12a presented the highest stringency on PAM recognition".

*Response:
We agree with the Reviewer that the cleavage patterns of CeCas12a and Bf Cas12a are similar to reported LbCas12a, FnCas12a and AsCas12a, including canonical and non-canonical PAMs. We also found some big differences in their cleavage activities towards non-canonical by in vitro cleavage assay and analysis of cleavage kinetics. We compared the cleavage activities of CeCas12a to AsCas12a, LbCas12a, FnCas12a and BfCas12a in the context of the TTTV PAM and C-containing PAMs. The results showed that the suboptimal C-containing PAMs substrates were slightly (such as CTTA and TTCA PAMs) or even barely cleaved (such as TCTG and TCTC PAMs) by CeCas12a while the optimal PAM TTTV substrates were cleaved completely. To further conform the result of in vitro cleavage assays, we carried out nine reactions in parallel for Cas12a nucleases by incubating for a different amount of time for each candidate to assess cleavage kinetics using Deep sequencing. Sequencing reads were filtered by Phred quality (≥30 for all of 256 kinds of PAM bases) and analyzed using a custom Python script to estimate cleavage rates[1]. For each cleavage reaction, the depletion ratio (normalized read count in cleavage reaction)/(normalization read count in negative control) was calculated and then divided by the median depletion ratio of all VRRT sequences, which were not cleaved by Cas12a nucleases. Each PAM (256 total) deletion ratio for each Cas12a nuclease was fit to non-linear least squares in an exponential decay model (y(t) = Ae−kt, where y(t) is the normalized PAM count, t is the time (minutes), A is a constant and k is the rate constant[1, 2]. Results showed that CeCas12a had little or no activity at those C-containing PAMs. The result has been put in the revised manuscript:

Response Figure 2. Time-course in vitro cleavage reaction profiles of Cas12a orthologs towards C-containing PAM substrates (revised manuscript: Additional file 1: Figure S8C)*
 
3. Response to comment: More endogenous loci and statistical analysis should be included for measuring the nuclease activity of CeCas12a.

*Response:
Thanks a lot for good advices. We detected another 31 endogenous target sites including CTLA4, B2M, AAVS, CFTR, EGFR, GRIN2B, GDF15, CXCR4, Angptl3, CCR5, MMP9, RUNX1. Activities assessed by T7E1 assay. Indel percentage was determined by the formula, 100 × (1 - sqrt(b + c)/(a + b + c)), where a is the integrated intensity of the undigested PCR product; b and c are the integrated intensities of the cleavage product[3]. The result has been put in the revised manuscript:

Response Figure 3. Summaries of the activities of CeCas12a at TTTV PAMs at a diverse panel of target sites in HEK293T cells. (revised manuscript: Additional file 1: Figure S12; Fig. 2F)*
 
4. Response to comment: Targeted deep sequencing of a small number of selected sites might not be strong enough to support the specificity claim. Given that CeCas12a specificity is a major point of this paper, the author should carry out a comprehensive comparative analysis using high-throughput and genome-wide methods (eg. GUIDE-Seq, etc.), as well as the high-fidelity version of Cas12a (eg. enAsCas12a-HF1). To include other Cas proteins (eg. Cas9 and Cas12b) for comparison is not essential but will be very interesting.

*Response:
The reviewer has made a good suggestion and we totally agree with the reviewer that using high-throughput and genome-wide methods to detect off-targets would be stronger to support the specificity claim. In the original manuscript, 41 predicted off-target sites were checked for CeCas12a, AsCas12a and LbCas12a and most of them do not show off-target cleavage. Three of them showing off-targeting cleavage were further compared. In the revised manuscript, in order to carry out a comprehensive comparative analysis using high-throughput and genome-wide methods for CRISPR-Cas nucleases including AsCas12a, enAsCas12a-HF1, LbCas12a, spCas9 and BhCas12b v4, the genome-wide unbiased identification of double-stranded breaks (DSBs) enabled by sequencing (GUIDE-seq) method[4-6] was used. Due to the very low off-target rates of Cas12a, five endogenous target sites showing off-targeting and containing overlapping target sites (except for HEK293 site 1 and DNMT1) for these Cas12a/b and Cas9 were identified. It shows CeCas12a has very low off-targeting in general and the result has been put in the revised manuscript:

Response Figure 4. Assessment of off-target effects for CRISPR-Cas nucleases. (revised manuscript: Fig. 4E, F, G)

Response Figure 5. GUIDE-seq analysis of detected off-targets in Fig. 4F (CCR5, POLQ target 2, IL12A). Matched target sites for Cas12a, Cas12b and SpCas9 that share a common protospacer sequence. (revised manuscript: Additional file 1: Figure S15).*

5. Minor point 1: The authors states that they identify 5 new Cas12a proteins which have not previously been employed for genome editing. However, BfCas12a has been characterized in a preprint paper published in bioRxiv (Zetsche et al., dio.org/10.1101/134015). Thus, the statement should be rewritten, and this reference should be cited.

*Response:
We are very sorry for not noticing the BfCas12a paper in bioRxiv which do not have the results of genome editing on endogenous sites in human cells. The statement has been rewritten as“another 5 reported Cas12a nucleases (SsCas12a from Smithella sp.SC_K08D17, Lb3Cas12a from Lachnospiraceae bacterium MC2017, BpCas12a from Bytyrivibrio, PdCas12a from Prevotella disens, BfCas12a from Butyrivibrio fibrisolvens MD2001) which have not been employed for genome editing on endogenous sites in human cells” in revised manuscript, and the reference has been cited. (revised manuscript Reference 22)*

6. Minor point 1: In this manuscript, 12 Cas12a orthologs are included, but information of several proteins is omitted. Please add the description or related citation in the right place.

*Response:
We are very sorry for our omission of another 4 Cas12a nucleases. We have added the description and related citation in the right place.
“SsCas12a from Smithella sp.SC_K08D17, Lb3Cas12a from Lachnospiraceae bacterium MC2017, BpCas12a from Bytyrivibrio, PdCas12a from Prevotella disens”. (revised Reference 14)

Special thanks for your good comments.*

Reviewer #2
1. Response to comment: In vitro cleavage efficiencies should be quantified for all figures. One of the authors' key arguments for the manuscript is that CeCas12a is more specific for PAM recognition than other Cas12a orthologues tested, but the reader has to compare band intensities by eye across several gels to draw that conclusion. A bar graph of efficiencies for each PAM would be a clearer Fig. 1. I would keep the gel images but move them to the supplement.

*Response:
We are very grateful to your kind advices. We adapted standard test to determine the PAM by using deep sequencing, and the specificity of PAM recognition can be identified by Heat map and time-course in vitro cleavage reactions. Normalized cleavage rates for all 4-base PAMs for CeCas12a, BfCas12a, LbCas12a and AsCas12a were shown below and in Fig. 1E of the revised manuscript. The intensity of color represented the activity of Cas12a nuclease. Quantification of time-course in vitro cleavage reactions of Cas12a orthologs on linearized dsDNA substrates was conducted at nine time points respectively. It was also shown below and in Fig. 1F of the revised manuscript. Curves were fit using one phase exponential decay equation. So we moved the gel images to the supplement (Additional file 1: Figure S4). The result has been put in the revised manuscript:

Response Figure 6. DNA cleavage of Cas12a orthologs in vitro. (revised manuscript: Fig. 1E, F)*

2. Response to comment: I couldn't find what statistical test was used to calculate P-values for Figs. 2, 3, and 4. Please include that information.

*Response:
We are very sorry for our negligence of statistical test. We have added this part in the end of revised manuscript. “Statistical significance was calculated using Mann-Whitney tests using GraphPad Prism version 8.0. The error bars in all figures show standard error of the mean (n=3). P values are reported using GraphPad style: not significant (ns), P > 0.05; *, P < 0.05; **, P < 0.01; ***, P <0.001.”*

Minor Comments

1. Response to comment: Italicize species names throughout.

*Response:
Thanks a lot for your kind advice. Species names have been italicized throughout of the revised manuscript.*

2. Response to comment: Lines 104-105: Is the cleavage assay data for other Cas12a orthologs not shown?

*Response:
We have added this data in Additional file 1: Figure S5, 6, 7 in the revised manuscript.

Response Figure 7. DNA cleavage activity of BhCas12a in vitro. (revised manuscript: Additional file 1: Figure S5)

Response Figure 8. DNA cleavage activity of CsbCas12a in vitro. (revised manuscript: Additional file 1: Figure S6)

Response Figure 9. DNA cleavage activity of PrCas12a in vitro. (revised manuscript: Additional file 1: Figure S7)*

3. Response to comment: Line 113: I don't understand why the cleavage activity of CeCas12a and BfCas12a is surprising.

*Response:
We are very sorry for our incorrect writing. “surprising” has been deleted in the revised manuscript.*

4. Response to comment: Lines 171-174: I'm confused about the purpose of the dCas12a controls—were they just confirmed in vitro?

*Response:
In EGFP disruption assay, we transfected dCas12a and crRNA plasmid as the negative control. We have assessed the gene editing activities of Cas12a variants with catalytic residues mutations in vivo.

Response Figure 10. Abolished gene editing by mutating catalytic residues. (revised manuscript: Additional file 1: Figure S9)*

4. Response to comment: Lines 199-200: The authors should provide quantitative range of gene editing outcomes instead of vague descriptions like "pretty well."

*Response:
We are sorry for our vague descriptions. We quantified 31 endogenous target sites to assess the gene editing efficiencies of CeCas12a. The mean frequencies of indels range from 8% to 65% and the result has been put in the revised manuscript too. (revised manuscript: Additional file 1: Figure S12; Fig. 2F)*

5. Response to comment: Lines 250-253: I recommend showing this data as a bar graph because it's difficult to follow in written form.

*Response:
Thanks a lot for your advice. This data has been replaced as a bar graph and Lines 250-253 was replaced with the sentence “When substituting thymidine to cytidine at PAM position -2, -3 and -4, CeCas12a was in a very low activity at those C-containing PAMs (Fig. 3A)”

Response Figure 11. In vivo cleavage activities of Lb, As and Ce toward different PAMs. Indel frequencies for 82 endogenous target sites with the different PAMs were measured in mammalian cells by Deep sequencing. (revised manuscript: Fig. 3A)*

6. Response to comment: Fig. 4A: I recommend summarizing the important features of this table as a bar graph and moving the rest of the table to the supplement.

*Response:
Thanks a lot for your kind advice. We have drawn this as bar graph in the revised manuscript Fig. 4A, B, C, D and moved the rest of the table to the supplement (Additional file 1: Figure S13).

Response Figure 12. Assessment off-target effects of CRISPR-Cas nucleases. (revised manuscript: Fig. 4A, B, C, D)*

7. Response to comment: Lines 359-361: I'm not sure what you were trying to say here. Off target effects for CeCas12a are still determined by a combination of PAM recognition and individual spacer sequences, it's just that the spacer plays a more significant role for CeCas12a because PAM recognition is more specific.

*Response:
We are sorry for our vague descriptions. We totally agree with you. From Lines 359-361 “The case of CeCas12a indicated that off-targeting happened at the PAM region in vivo might be reflected by the ability of Cas12a on the restriction of PAM sequences in vitro to some extent”, we tended to say that the restriction of PAM sequences in vitro could lead to the more specific PAM recognition in vivo and screening could be performed in vitro first for finding useful genome editing tools more conveniently in future. We have changed this sentence to “For off-targeting at PAM region, the case of CeCas12a suggested that the more specific recognition of PAM sequences in vitro could be followed by the more specific PAM recognition in vivo” in the revised manuscript.

Special thanks to you for your good comments.

References
1. BP K, I-O, AA S, RT W, YE T, JY H, K C, MM W, JE H, I-O, et al: - Engineered CRISPR-Cas12a variants with increased activities and improved targeting ranges for gene, epigenetic and base editing. Nat Biotechnol 2019, 37:276-282.
2. L G, I-O, DBT C, WX Y, JC M, MW S, I-O, T Y, H N, O N, et al: - Engineered Cpf1 variants with altered PAM specificities. Nat Biotechnol 2017, 35:789-792.
3. M T, L L, Y C, X H, H S, H X, J F, C L, J L, D C, et al: - A 'new lease of life': FnCpf1 possesses DNA cleavage activity for genome editing in human cells. Nucleic Acids Res 2017, 45:11295-11304.
4. SQ T, Z Z, NT N, M L, VV T, V T, N W, C K, AJ I, LP L, et al: - GUIDE-seq enables genome-wide profiling of off-target cleavage by CRISPR-Cas nucleases. Nat Biotechnol 2015, 33:187-197.
5. BP K, SQ T, MS P, NT N, MM W, JM L, ZR M, MJ A, Id O, JK J: - Genome-wide specificities of CRISPR-Cas Cpf1 nucleases in human cells. Nat Biotechnol 2016, 34:869-874.
6. BP K, V P, MS P, SQ T, NT N, Z Z, JK J: - High-fidelity CRISPR-Cas9 nucleases with no detectable genome-wide off-target effects. Nature 2016, 529:490-495.*

**Second round of review**

**Reviewer 1**

Overall, the authors have significantly improved the manuscript to address the comments in the revision. However, several problems remain:
1) Cleavage buffer was used for in vitro DNA cleavage assay in the original manuscript, whereas Cutsmart buffer (NEB) was used for in vitro PAM identification assay in the revised experiments. Is there any difference on the in vitro activity of Cas12a in different buffers?
2) There are still some grammatical errors (eg. Line 161, ‘there were barely cleavages…’).

**Reviewer 2**

The authors have addressed all of my previous concerns.

**Authors Response**

**Point-by-point responses to the reviewers’ comments:**

Reviewer #1:
1. Response to comment: Overall, the authors have significantly improved the manuscript to address the comments in the revision. However, several problems remain:
1) Cleavage buffer was used for in vitro DNA cleavage assay in the original manuscript, whereas Cutsmart buffer (NEB) was used for in vitro PAM identification assay in the revised experiments. Is there any difference on the in vitro activity of Cas12a in different buffers?
2) There are still some grammatical errors (eg. Line 161, 'there were barely cleavages…').

*Response:
We thank the reviewer very much for his/her appreciation of our study.
1) There is no difference on the in vitro activity of Cas12a in two similar buffers and both buffers were used by many other people [1-5]. When doing the in vitro PAM identification assay, we used the method that described in the paper titled “Engineered Cpf1 variants with altered PAM specificities increase genome targeting range” [5]. And since they used Cutsmart buffer (NEB) in this assay, we simply followed that.

2) We have corrected the grammatical error, and the sentence “There were barely cleavages at C-containing PAM sites except the CTTA PAM site” has been changed to “There were few cleavages at C-containing PAM sites except the CTTA PAM site” in revised manuscript.

References
1. S H, H N, R I, O N: - Structural Basis for the Altered PAM Specificities of Engineered CRISPR-Cas9. Mol Cell 2016, 61:886-894.
2. M Y, Y W, JS G, H H, FA R, T N, R I, F Z, H N, O N: - Crystal Structure of the Minimal Cas9 from Campylobacter jejuni Reveals the Molecular Diversity in the CRISPR-Cas9 Systems. Mol Cell 2017, 65:1109-1121.
3. T Y, H N, B Z, H H, IM S, Y L, I F, T N, KS M, EV K, et al: - Crystal Structure of Cpf1 in Complex with Guide RNA and Target DNA. Cell 2016, 165:949-962.
4. H N, T Y, L G, F Z, R I, O N: - Structural Basis for the Altered PAM Recognition by Engineered CRISPR-Cpf1. Mol Cell 2017, 67:139-147.
5. L G, DBT C, WX Y, JC M, MW S, T Y, H N, O N, et al: - Engineered Cpf1 variants with altered PAM specificities. Nat Biotechnol 2017, 35:789-792.*
